# Supplementary material for: Proteo-genomic analyses in relatively lean Chinese adults identify proteins and pathways that affect general and central adiposity levels
Source: Commun Biol. 2024 Oct 15;7:1327. doi: 10.1038/s42003-024-06984-y (PMC11480319; doi:10.1038/s42003-024-06984-y)
Supplement: Supplementary file 2 — Supplementary Information [file 42003_2024_6984_MOESM2_ESM.pdf]

Supplementary Information

Proteo-genomic analyses in relatively lean Chinese adults identify proteins and pathways that affect general and central adiposity levels

Contents

Members of the China Kadoorie Biobank collaborative group 3

Supplementary Table 1. Baseline characteristics of participants by quintiles of measured WC 4

Supplementary Table 2. Baseline characteristics of participants with proteomic assays in CKB 5

Supplementary Table 3. Number of proteins significantly associated at FDR<0.05 with general and central adiposity in conventional and genetic analyses 6

Supplementary Table 4. Number of proteins associated with general and central adiposity in conventional and genetic analyses after Bonferroni correction 7

Supplementary Table 5. Number of proteins significantly associated at FDR<0.05 with general and central adiposity in conventional analyses after adjustments 8

Supplementary Table 6. List of significantly enriched biological process terms of adiposity-related proteins after accounting for the potential pre-enrichment of proteins included in Olink 9

Supplementary Table 7. Genetic effect estimates, PheWAS results, tissue expression, and relevant drug targets of 31 proteins showing genetic effects on BMI and WC 10

Supplementary Table 8. Colocalisation analyses between adiposity traits and selected proteins 12

Supplementary Table 9. PheWAS results of 31 proteins showing genetic effects on levels of BMI and WC 13

Supplementary Table 10. Number of proteins with QC warnings 14

Supplementary Table 11. Variance explained by adiposity genetic scores in CKB and UKB 15

Supplementary Table 12. Associations of potential confounders with BMI and WC GS 16

Supplementary Figure 1. Correlation between adiposity measures and genetic scores 17

Supplementary Figure 2. Number of proteins significantly associated with adiposity in conventional and genetic analyses 18

Supplementary Figure 3. Global comparison of associations of protein biomarkers with 1-SD higher BMI, BF%, WC and WHR in conventional and genetic analyses in OLINK batch 1 (black dots) and 2 (red dots) 19

Supplementary Figure 4. Number of proteins significantly associated with adiposity in conventional and genetic analyses after mutual adjustments 20

Supplementary Figure 5. Global comparison of associations of protein biomarkers with 1-SD higher BMI, BF%, WC and WHR in conventional and genetic analyses in OLINK batch 1 (black dots) and 2 (red dots), by sex 21
Supplementary Figure 6. Volcano plot of associations of proteins with 1-SD higher BMI, BF%, WC and WHR in conventional and genetic analyses in OLINK batch 1 (solid dots) and 2 (open dots) in the subcohort........ 22
Supplementary Figure 7. Comparison of associations of protein biomarkers with 1-SD higher BMI in genetic analyses in CKB and CKB vs. UKB using different CKB BMI GS23
Supplementary Figure 8. Comparison of associations of protein biomarkers with 1-SD higher BMI, BF%, WC and WHR in conventional and genetic analyses in CKB vs. UKB, with black dots indicating OLINK batch 1 and red dots indicating OLINK batch 2 24
Supplementary Figure 9. Correlation matrix between the 31 proteins showing causal relationships with adiposity........ 25
Supplementary Figure 10. Locus-zoom plots of colocalisation analyses........ 26
Supplementary Figure 11. Human tissux10-specific gene expression for the 31 proteins showing causal relationships with adiposity 36
Supplementary Figure 12. Flow diagram of study design and participant selection in CKB........ 37
Supplementary Figure 13. Distribution of proteins 38

Members of the China Kadoorie Biobank collaborative group

International Steering Committee: Junshi Chen, Zhengming Chen (PI), Robert Clarke, Rory Collins, Liming Li (PI), Jun Lv, Richard Peto, Robin Walters.

International Co-ordinating Centre, Oxford: Daniel Avery, Maxim Barnard, Derrick Bennett, Lazaros Belbasis, Ruth Boxall, Ka Hung Chan, Yiping Chen, Zhengming Chen, Charlotte Clarke, Johnathan Clarke; Robert Clarke, Huaidong Du, Ahmed Edris Mohamed, Hannah Fry, Simon Gilbert, Pek Kei Im, Andri Iona, Maria Kakkoura, Christiana Kartsonaki, Hubert Lam, Kuang Lin, James Liu, Mohsen Mazidi, Iona Millwood, Sam Morris, Qunhua Nie, Alfred Pozarickij, Maryanm Rahmati, Paul Ryder, Saredo Said, Dan Schmidt, Becky Stevens, Iain Turnbull, Robin Walters, Baihan Wang, Lin Wang, Neil Wright, Ling Yang, Xiaoming Yang, Pang Yao.

National Co-ordinating Centre, Beijing: Xiao Han, Can Hou, Qingmei Xia, Chao Liu, Jun Lv, Pei Pei, Dianjianyi Sun, Canqing Yu

10 Regional Co-ordinating Centres:

Guangxi Provincial CDC: Naying Chen, Duo Liu, Zhenzhu Tang. Liuzhou CDC: Ningyu Chen, Qilian Jiang, Jian Lan, Mingqiang Li, Yun Liu, Fanwen Meng, Jinhuai Meng, Rong Pan, Yulu Qin, Ping Wang, Sisi Wang, Liuping Wei, Liyuan Zhou. **Gansu** Provincial CDC: Caixia Dong, Pengfei Ge, Xiaolan Ren. Maiji CDC: Zhongxiao Li, Enke Mao, Tao Wang, Hui Zhang, Xi Zhang. **Hainan** Provincial CDC: Jinyan Chen, Ximin Hu, Xiaohuan Wang. Meilan CDC: Zhendong Guo, Huimei Li, Yilei Li, Min Weng, Shukuan Wu. **Heilongjiang** Provincial CDC: Shichun Yan, Mingyuan Zou, Xue Zhou. Nangang CDC: Ziyan Guo, Quan Kang, Yanjie Li, Bo Yu, Qinai Xu. **Henan** Provincial CDC: Liang Chang, Lei Fan, Shixian Feng, Ding Zhang, Gang Zhou. Huixian CDC: Yulian Gao, Tianyou He, Pan He, Chen Hu, Huarong Sun, Xukui Zhang. **Hunan** Provincial CDC: Biyun Chen, Zhongxi Fu, Yuelong Huang, Huilin Liu, Qiaohua Xu, Li Yin. Liuyang CDC: Huajun Long, Xin Xu, Hao Zhang, Libo Zhang. **Jiangsu** Provincial CDC: Jian Su, Ran Tao, Ming Wu, Jie Yang, Jinyi Zhou, Yonglin Zhou. Suzhou CDC: Yihe Hu, Yujie Hua, Jianrong Jin, Fang Liu, Jingchao Liu, Yan Lu, Liangcai Ma, Aiyu Tang, Jun Zhang. **Qingdao** CDC: Liang Cheng, Ranran Du, Ruqin Gao, Feifei Li, Shanpeng Li, Yongmei Liu, Feng Ning, Zengchang Pang, Xiaohui Sun, Xiaocao Tian, Shaojie Wang, Yaoming Zhai, Hua Zhang, Licang CDC: Wei Hou, Silu Lv, Junzheng Wang. **Sichuan** Provincial CDC: Xiaofang Chen, Xianping Wu, Ningmei Zhang, Xiaoyu Chang. Pengzhou CDC: Xiaofang Chen, Jianguo Li, Jiaqiu Liu, Guojin Luo, Qiang Sun, Xunfu Zhong. **Zhejiang** Provincial CDC: Weiwei Gong, Ruying Hu, Hao Wang, Meng Wang, Min Yu. Tongxiang CDC: Lingli Chen, Qijun Gu, Dongxia Pan, Chunmei Wang, Kaixu Xie, Xiaoyi Zhang.

Supplementary Table 1. Baseline characteristics of participants by quintiles of measured WC

Characteristics[a]	Sex-specific quintiles of WC, kg/m^2 Q1 (n=798)	Q2 (n=788)	Q3 (n=797)	Q4 (n=807)	Q5 (n=787)	All (n=3977)
**Age and socioeconomic factors**						
Age, years (SD)	55.9 (14.5)	55.1 (12.9)	56.5 (11.8)	58.2 (12.1)	58.4 (12.6)	57.3 (11.6)
Women, %	53.4	53.6	52.1	54.8	55.9	53.7
Urban, %	29.9	38.5	52.0	56.9	61.7	48.8
≥6 years of education, %	46.4	43.8	45.2	45.7	41.7	45.1
**Anthropometry, blood pressure and fasting time, mean (SD)**						
BMI, kg/m^2	19.9 (2.1)	22.0 (1.8)	23.8 (2.0)	25.4 (2.0)	28.5 (3.0)	23.9 (3.3)
Body fat percentage	20.3 (4.2)	24.6 (4.1)	28.1 (4.9)	30.8 (4.6)	35.1 (5.9)	25.4 (6.3)
Waist circumference, cm	68.4 (3.5)	75.9 (1.8)	81.5 (1.6)	87.0 (1.9)	96.5 (6.4)	81.9 (9.1)
Waist-to-hip ratio	0.81 (0.042)	0.86 (0.038)	0.90 (0.039)	0.93 (0.044)	0.97 (0.064)	0.89 (0.001)
SBP, mmHg	129.5 (22.9)	134.8 (21.7)	139.2 (22.0)	141.1 (22.1)	147.7 (25.3)	138.3 (22.0)
Fasting time, hour	4.6 (4.3)	4.9 (4.3)	4.7 (4.3)	4.5 (3.6)	4.5 (3.5)	4.7 (4.1)
**Lifestyle factors**						
Ever regular smoker**, %**						
Men	83.2	70.1	75.7	69.7	77.9	75.0
Women	4.3	6.3	4.4	6.7	5.1	5.8
Regular alcohol drinker**, %**						
Men	31.8	31.1	40.2	36.2	28.5	34.6
Women	2.5	1.8	2.5	4.3	4.3	3.0
Physical activity, MET-h/day (SD)	17.7 (12.1)	17.7 (12.3)	17.2 (10.7)	17.0 (10.5)	15.6 (10.5)	17.3 (10.7)
**Medical history and health status,[b] %**						
Self-rated poor health	16.2	10.6	8.0	9.0	12.7	16.6
Diabetes	4.7	7.7	9.5	12.5	19.4	11.2
Chronic kidney disease	1.4	1.7	2.1	0.7	1.7	1.4
Cancer	1.1	0.3	0.5	0.4	0.5	0.6

[a] Adjusted for age, sex and study area, as appropriate.
[b] Based on self-report, while for diabetes, screen-detected diabetes at baseline was also included.
Abbreviations: SD=standard deviation; BMI= body mass index; SBP=systolic blood pressure; MET= metabolic equivalent of task.

Supplementary Table 2. Baseline characteristics of participants with proteomic assays in CKB

Characteristics [a]	All (n=3977)	Subcohort (n=2026)	IHD Cases (n=1951)
**Age and socioeconomic factors**			
Age, years (SD)	57.3 (11.6)	51.3 (10.4)	52.2 (10.6)
Women, %	53.7	62.1	45.1
Urban, %	48.8	50.6	47.0
≥6 years of education, %	45.1	52.1	37.7
**Anthropometry, blood pressure and fasting time, mean (SD)**			
BMI, kg/m^2	23.9 (3.3)	23.9 (3.2)	24.0 (3.4)
Body fat percentage	25.4 (6.3)	28.6 (6.3)	27.0 (6.7)
Waist circumference, cm	81.9 (9.1)	80.3 (8.7)	83.6 (9.4)
Waist-to-hip ratio	0.89 (0.001)	0.88 (0.062)	0.91 (0.067)
SBP, mmHg	138.3 (22.0)	130.6 (19.7)	146.3 (23.5)
Fasting time, hour	4.7 (4.1)	5.1 (4.3)	4.3 (4.0)
**Lifestyle factors**			
Ever regular smoker**, %**			
Men	75.0	74.9	75.1
Women	5.8	3.3	9.3
Regular alcohol drinker**, %**			
Men	34.6	40.1	30.6
Women	3.0	2.7	3.4
Physical activity, MET-h/day (SD)	17.3 (10.7)	21.3 (12.3)	13.0 (8.7)
**Medical history and health status,[b] %**			
Self-rated poor health	16.6	13.4	19.8
Diabetes	11.2	16.1	6.4
Chronic kidney disease	1.4	1.4	1.3
Cancer	0.6	0.6	0.6

[a] Adjusted for age, sex and study area, as appropriate.

[b] Based on self-report, while for diabetes, screen-detected diabetes at baseline was also included.

Abbreviations: SD=standard deviation; BMI= body mass index; IHD=ischaemic heart disease; SBP=systolic blood pressure; MET= metabolic equivalent of task.

Supplementary Table 3. Number of proteins significantly associated at FDR<0.05 with general and central adiposity in conventional and genetic analyses

Adiposity	Batch 1 (n=1463)	Batch 2 (n=1463)	Total (n=2926)
**Conventional analyses**			
BMI	1088 (73.9)	721 (49.0)	1809 (61.8)
BF%	1115 (75.7)	723 (49.1)	1838 (62.8)
WC	1099 (74.7)	709 (48.2)	1808 (61.8)
WHR	1055 (71.7)	681 (46.3)	1736 (59.3)
**Any**	1237 (84.0)	859 (58.4)	2096 (71.6)
**All**	937 (63.7)	576 (39.1)	1513 (51.7)
**Genetic analyses**			
BMI	304 (20.7)	95 (6.5)	399 (13.6)
BF%	212 (14.4)	27 (1.8)	239 (8.2)
WC	365 (24.8)	71 (4.8)	436 (14.9)
WHR	261 (17.7)	25 (1.7)	286 (9.8)
**Any**	542 (36.8)	120 (8.2)	662 (22.6)
**All**	75 (5.1)	5 (0.3)	80 (2.7)

Abbreviations: BF%=body fat percentage; BMI=body mass index; WC= waist circumference; WHR=waist-to-hip ratio

Supplementary Table 4. Number of proteins associated with general and central adiposity in conventional and genetic analyses after Bonferroni correction

Adiposity	Batch 1 (n=1463)	Batch 2 (n=1463)	Total (n=2926)
**Conventional analyses**			
BMI	798 (54.2)	461 (31.4)	1259 (42.8)
BF%	836 (56.8)	476 (32.4)	1312 (44.6)
WC	824 (56.0)	479 (32.7)	1303 (44.3)
WHR	782 (53.1)	460 (31.4)	1242 (42.3)
**Any**	925 (62.8)	541 (36.9)	1466 (49.9)
**All**	691 (46.9)	398 (27.1)	1089 (37.1)
**Genetic analyses**			
BMI	58 (3.8)	28 (1.8)	86 (2.8)
BF%	34 (2.3)	5 (0.3)	39 (1.3)
WC	98 (6.7)	25 (1.7)	123 (4.2)
WHR	45 (3.1)	8 (0.5)	53 (1.8)
**Any**	151 (10.3)	42 (2.9)	193 (6.6)
**All**	3 (0.2)	1 (0.1)	4 (0.1)

Abbreviations: BF%=body fat percentage; BMI=body mass index; WC= waist circumference; WHR=waist-to-hip ratio

Supplementary Table 5. Number of proteins significantly associated at FDR<0.05 with general and central adiposity in conventional analyses after adjustments

Adiposity	Batch 1 (n=1463)	Batch 2 (n=1463)	Total (n=2926)
**Usual adjustments***			
BMI	1088 (73.9)	721 (49.0)	1809 (61.8)
BF%	1115 (75.7)	723 (49.1)	1838 (62.8)
WC	1099 (74.7)	709 (48.2)	1808 (61.8)
WHR	1055 (71.7)	681 (46.3)	1736 (59.3)
**Any**	1237 (84.0)	859 (58.4)	2096 (71.6)
**All**	937 (63.7)	576 (39.1)	1513 (51.7)
**Usual adjustments* + physical activity**			
BMI	1088 (74.4)	720 (49.4)	1808 (61.8)
BF%	1116 (76.3)	721 (49.5)	1837 (62.8)
WC	1099 (75.1)	708 (48.6)	1807 (61.8)
WHR	1055 (72.1)	681 (46.7)	1736 (59.4)
**Any**	1238 (84.0)	857 (58.4)	2095 (71.6)
**All**	937 (64.0)	576 (39.5)	1513 (51.7)
**Usual adjustments* + smoking**			
BMI	1078 (73.7)	722 (49.5)	1800 (61.6)
BF%	1125 (75.7)	723 (49.6)	1848 (63.3)
WC	1097 (75.0)	707 (48.5)	1804 (61.8)
WHR	1058 (72.3)	682 (46.8)	1740 (59.6)
**Any**	1239 (84.0)	852 (58.3)	2091 (71.6)
**All**	944 (64.4)	582 (39.9)	1526 (52.2)
**Usual adjustments* + alcohol**			
BMI	1090 (74.5)	721 (49.5)	1811 (61.9)
BF%	1120 (76.6)	723 (49.6)	1843 (63.1)
WC	1104 (75.5)	708 (48.6)	1812 (62.0)
WHR	1064 (72.7)	680 (46.6)	1744 (59.7)
**Any**	1205 (82.4)	858 (58.9)	2099 (71.8)
**All**	941 (63.3)	580 (39.8)	1521 (52.1)
**Usual adjustments* + physical activity + smoking + alcohol**			
BMI	1078 (73.7)	726 (49.8)	1804 (61.7)
BF%	1116 (76.3)	722 (49.5)	1838 (62.9)
WC	1099 (75.1)	705 (48.4)	1804 (61.8)
WHR	1070 (73.1)	679 (46.6)	1749 (59.9)
**Any**	1239 (84.6)	855 (58.6)	2094 (71.6)
**All**	949 (63.7)	583 (39.9)	1532 (52.3)

*Usual adjustments: age, age squared, sex, study area, fasting time, ambient temperature, ascertainment status

Supplementary Table 6. List of significantly enriched biological process terms of adiposity-related proteins after accounting for the potential pre-enrichment of proteins included in Olink

Adiposity	ID	Description	GeneRatio	BgRatio	pvalue	p.adjust	qvalue	geneID	Count
BF%	GO:0051241	negative regulation of multicellular organismal process	15/39	328/2759	1.68E-05	0.026393	0.02491	LEP/ADM/VSIG4/LBP/RTN4R/CALCA/CST3/FSTL3/TNFRSF1A/NTRK3/LGALS9/OXT/FURIN/PLAT/ACVRL1	15
WC	GO:0009628	response to abiotic stimulus	31/121	311/2759	5.01E-06	0.011967	0.011026	PRAP1/LEP/PGF/ADM/CALCA/NOS3/DAG1/PLAT/TNFRSF10B/CTSS/OXT/PLIN1/TNFRSF1A/SCARA5/CDH2/YAP1/HNMT/FAS/TNFRSF10A/IGFBP2/STC1/LPL/TNFRSF11A/TGFBR2/ACVRL1/CXCL10/IGFBP7/VEGFB/F7/KRT8/SERPINF1	31
WHR	GO:0042632	cholesterol homeostasis	6/50	28/2759	7.25E-06	0.005313	0.005116	LDLR/LCAT/CES1/LPL/FGFR4/FABP4	6
WHR	GO:0055092	sterol homeostasis	6/50	28/2759	7.25E-06	0.005313	0.005116	LDLR/LCAT/CES1/LPL/FGFR4/FABP4	6
WHR	GO:0010876	lipid localization	9/50	103/2759	6.93E-05	0.025396	0.024452	LDLR/FURIN/ADIPOQ/LCAT/CES1/LPL/PRAP1/FABP4/OXT	9

Supplementary Table 7. Genetic effect estimates, PheWAS results, tissue expression, and relevant drug targets of 31 proteins showing genetic effects on BMI and WC

Adiposity	Protein[a]	SNP information		CKB cis-pQTL		Two-sample MR[c]		UKB replication[e]	PheWAS associations[f]	Levels of expression[g]					Drug Target		
		rsID	EA[b]	Beta (SE)	P	Beta (SE)[d]	P			Adipose	Brain	Liver	Pancreas	Intestine	Disease	Max Phase	Status
**BMI**	<u>**ADH1B**</u>	rs1229984	C	0.204 (0.024)	5.9×10^{-17}	0.127 (0.028)	9.6×10^{-06}	-	**Fat % of leg/whole body**	+++	+	+++	++	++	Alcohol dehydrogenase class I	IV	Approved
	ALDH5A1	rs2235502	C	0.314 (0.043)	2.6×10^{-13}	-0.056 (0.019)	3.8×10^{-03}	-	-	++	+++	+++	++	++	Bipolar disorder, Epilepsy, Generalised not-motor seizure,	IV	Approved
	APOBR	rs180743	C	1.199 (0.036)	1.2×10^{-246}	-0.057 (0.011)	1.4×10^{-07}	-	**BMI, Body fat%, Fat mass leg/arm**	+	+	+	+	++	-	-	-
	**APOE**	rs429358	T	1.030 (0.038)	4.9×10^{-158}	0.028 (0.006)	1.5×10^{-05}	-	**BMI, Body fat%, Fat mass leg/arm/ body, Weight change**	+++	+++	+++	++	++	Glioblastoma multiform, Brain neoplasm	II	Completed
	ASAH1	rs2299604	C	0.461 (0.023)	5.0×10^{-86}	-0.024 (0.008)	2.5×10^{-03}	X	-	++	++	+	++	++	-	-	-
	BMPER	rs6951634	C	0.357 (0.039)	2.4×10^{-20}	0.043 (0.013)	1.3×10^{-03}	-	-	+	+	+	+	+	-	-	-
	CACYBP	rs12563930	G	0.174 (0.027)	6.8×10^{-11}	-0.122 (0.031)	1.0×10^{-04}	-	**BMI, Impedance of arm/whole body, Fat mass/% of leg**	++	++	+	+	++	-	-	-
	CD164L2	rs79775449	T	0.178 (0.025)	1.1×10^{-12}	-0117 (0.030)	8.6×10^{-05}	-	BMI	+	+	+	+	+	-	-	-
	CFB	rs1265888	G	0.820 (0.048)	1.5×10^{-65}	0.036 (0.008)	2.8×10^{-06}	√	**Impedance of arm/leg/whole body, Fat-free mass of trunk/leg**	++	+	+++	+	++	Paroxysmal nocturnal hemoglobinuria, Glomerunephritis, Atypical haemolytic-uremic syndrome	III	Completed/ Recruiting
	CFHR2	rs4085749	C	1.035 (0.024)	2.1×10^{-299}	0.013 (0.004)	6.80×10^{-04}	X	-	+	+	++	+	+	-	-	-
	EFEMP1	rs58680090	A	0.166 (0.028)	4.30×10^{-09}	0.110 (0.031)	4.70x10-04	√	**Impedance of whole body, Fat-free mass arm/leg/trunk, Fat and fat-free mass of trunk, Height**	++	++	+	++	++	-	-	-
	F13B	rs12134960	G	0.505 (0.024)	1.8×10^{-97}	0.026 (0.008)	8.2×10^{-04}	X	-	+	+	++	+	+	-	-	-
	FGL1	rs3739404	A	0.546 (0.023)	1.1×10^{-124}	0.020 (0.006)	1.2×10^{-03}		-	+	+	+++	++	+	-	-	-
	**FN1**	rs1437787	C	0.297 (0.041)	3.0×10^{-13}	-0.084 (0.028)	2.5×10^{-03}	√	-	++	++	++	+	++	Eye disease	IV	Approved
	ITIH3	rs2286797	G	0.730 (0.033)	7.9×10^{-107}	-0.26 (0.006)	9.9×10^{-06}	√	BMI	+	+	+++	+	+	-	-	-
	ITIH4	rs6803519	T	0.282 (0.024)	4.5×10^{-31}	0.070 (0.014)	1.7×10^{-06}	-	**Height**	++	+	+++	++	+	-	-	-
	**LRP11**	rs9688902	T	0.662 (0.030)	4.7×10^{-113}	0.030 (0.007)	$8.\ 5\times10^{-06}$	X	BMI	++	++	+	+	+	-	-	-
	**NCR3LG1**	rs2051772	A	0.356 (0.024)	4.6×10^{-51}	-0.042 (0.011)	1.4×10^{-04}	-	**BMI, Impedance of arm/leg/whole body, Height**	+	+	+	+	+	-	-	-
	NUDT5	rs7100710	A	0.154 (0.023)	2.4×10^{-11}	-0.122 (0.029)	3.8×10^{-05}	X	**BMI**	++	+	++	+	++	-	-	-
	<u>**OGN**</u>	rs7023004	G	0.598 (0.031)	1.5×10^{-80}	0.033 (0.009)	3.2×10^{-04}	√	**Impedance of arm/leg/whole body, Height**	++	+	+	+	++	-	-	-
	PRDX6	rs61826753	A	0.370 (0.025)	1.8×10^{-51}	0.036 (0.011)	6.8×10^{-04}	-	-	+++	++	++	++	++	-	-	-
	PRSS53	rs11150606	C	0.861 (0.028)	2.2×10^{-201}	0.013 (0.004)	1.5×10^{-03}	-	-	+	+	++	+	+	-	-	-
	PTPRB	rs2465811	T	0.239 (0.024)	1.6×10^{-22}	-0.050 (0.016)	2.3×10^{-03}	X	-	++	+	+	+	++	-	-	-
	PTPRZ1	rs28379507	C	0.171 (0.025)	3.4×10^{-12}	-0.079 (0.026)	2.4×10^{-03}	X	-	+	++	+	+	+	-	-	-
	SCAMP3	rs4971072	A	0.185 (0.026)	1.0×10^{-12}	0.115 (0.027)	2.7×10^{-05}	X	-	++	++	+	+	++	-	-	-
	<u>**SERPINF2**</u>	rs8077638	C	0.240 (0.032)	3.7×10^{-14}	0.101 (0.023)	2.0×10^{-05}	√	-	++	+	+++	+	++	-	-	-
	SLURP1	rs1036385	T	0.353 (0.024)	3.0×10^{-50}	-0.043 (0.014)	2.7×10^{-03}	-	-	+	+	+	+	+	-	-	-
	SUSD5	rs6764459	T	0.567 (0.024)	1.5×10^{-127}	-0.023 (0.008)	3.7×10^{-03}	-	-	+	+	+	+	+	-	-	-
	**TSPAN8**	rs9683252	G	0.700 (0.025)	7.5×10^{-166}	-0.022 (0.005)	5.4×10^{-05}	-	-	+	+	+	++	+++	-	-	-
	TXNDC15	rs3733897	G	1.045 (0.025)	2.0×10^{-317}	0.012 (0.004)	4.5×10^{-04}	√	Height	++	+	+	+	++	-	-	-

**WC**	**ALDH2**	rs4646776	G	0.371 (0.028)	6.3×10^{-41}	0.183 (0.027)	2.0X10^{-11}	-	-	++	++	++	++	++	Alcohol dependence, parasitic infection	IV	Approved
	**ITIH4**	rs6803519	T	0.282 (0.024)	4.5×10^{-31}	0.081 (0.019)	1.7X10^{-05}	-	**Height**	++	+	+++	++	+	-	-	-

[a]Evidence of colocalization (PH4>0.6): bold; Strong evidence of colocalization (PH4>0.8): bold and underline; [b]EA referred to allele associated with increased protein levels in CKB, and EAF affect allele was obtained from Biobank Japan; [c]2SMR: cis-pQTL obtained from CKB, with lookups in Biobank Japan for GWAS and Korean Biobank Project summary statistics; [d]Beta and SE were calculated using Wald ratio method; [e]2SMR: cis-pQTL obtained from UKB, with lookups in GIANT for GWAS summary statistic; [f]Traits or diseases in bold: $P<5\times10^{-8}$, other were $P<5\times10^{-6}$; [g]Levels of expression was estimated using GTEx and categorized into three groups as denoted: +(low); ++ (moderate) and +++ (high)

Supplementary Table 8. Colocalisation analyses between adiposity traits and selected proteins

Adiposity	Protein	No. SNPs	PP0	PP1	PP2	PP3	PP4
**BMI**	ADH1B	2567	$6.3x10^{-13}$	$8.2x10^{-06}$	$2.0x10^{-10}$	$5.4x10^{-04}$	$1.0x10^{+00}$
	ALDH5A1	947	$3.0x10^{-08}$	$4.9x10^{-01}$	$3.1x10^{-08}$	$5.0x10^{-01}$	$1.3x10^{-02}$
	APOBR	-	-	-	-	-	-
	APOE	803	$1.4x10^{-132}$	$1.8x10^{-03}$	$2.6x10^{-130}$	$3.4x10^{-01}$	$6.6x10^{-01}$
	ASAH1	-	-	-	-	-	-
	BMPER	-	-	-	-	-	-
	CACYBP	3390	$2.5x10^{-10}$	$1.5x10^{-04}$	$1.4x10^{-06}$	$7.8x10^{-01}$	$2.2x10^{-01}$
	CD164L2	686	$8.2x10^{-09}$	$1.7x10^{-01}$	$3.6x10^{-08}$	$7.5x10^{-01}$	$8.4x10^{-02}$
	CFB	-	-	-	-	-	-
	CFHR2	-	-	-	-	-	-
	EFEMP1	-	-	-	-	-	-
	F13B	-	-	-	-	-	-
	FGL1	-	-	-	-	-	-
	FN1	457	$1.6x10^{-07}$	$2.3x10^{-01}$	$7.6x10^{-08}$	$1.1x10^{-01}$	$6.6x10^{-01}$
	ITIH3	7096	$2.9x10^{-61}$	$2.3x10^{-10}$	$1.3x10^{-51}$	$1.0x10^{+00}$	$9.4x10^{-04}$
	ITIH4	7568	$7.4x10^{-34}$	$1.1x10^{-10}$	$6.5x10^{-24}$	$1.0x10^{+00}$	$1.7x10^{-04}$
	LRP11	4186	$1.xX10^{-117}$	$6.1x10^{-04}$	$6.7x10^{-115}$	$3.1x10^{-01}$	$6.9x10^{-01}$
	NCR3LG1	1116	$1.4x10^{-43}$	$8.0x10^{-03}$	$4.9x10^{-42}$	$2.8x10^{-01}$	$7.1x10^{-01}$
	NUDT5	973	$6.8x10^{-08}$	$1.9X10^{-04}$	$3.6x10^{-04}$	$9.9x10^{-01}$	$8.0x10^{-0}3$
	OGN	4061	$4.4x10^{-12}$	$2.3x10^{-03}$	$3.5x10^{-10}$	$1.8x10^{-01}$	$8.2x10^{-01}$
	PRDX6	3430	$9.1x10^{-47}$	$1.8x10^{-04}$	$4.9x10^{-43}$	$1.0x10^{+00}$	$3.4x10^{-03}$
	PRSS53	3166	$7.0x10^{-317}$	$3.9x10^{-02}$	$1.7x10^{-315}$	$9.6x10^{-01}$	$3.6x10^{-03}$
	PTPRB	-	-	-	-	-	-
	PTPRZ1	-	-	-	-	-	-
	SCAMP3	998	$3.0x10^{-12}$	$1.5x10^{-06}$	$2.0x10^{-06}$	$9.7x10^{-01}$	$2.9x10^{-02}$
	SERPINF2	144	$8.0x10^{-19}$	$5.3x10^{-04}$	$7.1x10^{-17}$	$4.6x10^{-02}$	$9.5x10^{-01}$
	SLURP1	1902	$0.0x10^{+00}$	$0.0x10^{+00}$	$4.7x10^{-04}$	$1.0x10^{+00}$	$3.4x10^{-03}$
	SUSD5	-	-	-	-	-	-
	TSPAN8	1577	$4.0x10^{-84}$	$6.8x10^{-03}$	$2.1x10^{-82}$	$3.4x10^{-01}$	$6.5x10^{-01}$
	TXNDC15	2821	$0.0x10^{+00}$	$3.7x10^{-03}$	$0.0x10^{+00}$	$1.0x10^{+00}$	$2.2x10^{-04}$
**WC**	ALDH2	6258	$3.8 x10^{-63}$	$9.4 x10^{-31}$	$8.3 x10^{-35}$	$1.8 x10^{-02}$	$9.8 x10^{-01}$
	ITIH4	6640	$2.1 x10^{-26}$	$3.3 x10^{-03}$	$1.1 x10^{-24}$	$1.6 x10^{-01}$	$8.3 x10^{-01}$

Abbreviations: BMI=body mass index; PP=Posterior probabilities; WC=waist circumference

Supplementary Table 9. PheWAS results of 31 proteins showing genetic effects on levels of BMI and WC

Adiposity	Protein	rsID	Proxy	PheWAS associations[a]	
				Traits	Disease
BMI	ADH1B	rs1229984	None	**Alcohol, SBP, Whole body fat, Leg fat percentage mass**	**Esophageal, head and neck and**
WC	ALDH2	rs4646776	None	**Alcohol**	**Esophageal cancer**
BMI	ALDH5A1	rs2235502	AFR/AMR/EAS/EUR/SAS	**-**	Allergy, Cause of death: cerebrum,
BMI	APOBR	rs180743	None	**Alcohol, BMI, Body fat percentage, Weight, Whole body fat, WC, HC, Leg and arm fat mass, Education**	**Inflammatory bowel disease**
BMI	APOE	rs429358	None	**BMI, Body fat percentage, Blood pressure, Cholesterol, C-reactive protein, Leg and arm fat mass, Pulse rate, Physical activity, WC, Weight**	**Alzheimer disease, Coronary artery disease, Dementia, Diabetes**
BMI	ASAH1	rs2299604	-	-	-
BMI	BMPER	rs6951634	AMR/EAS/EUR/SAS	-	Alzheimer disease
BMI	CACYBP	rs12563930	None	**BMI, Impedance of whole body and arm, Leg fat mass and percentage**	-
BMI	CD164L2	rs79775449	None	BMI	**-**
BMI	CFB	rs1265888	None	**Impedance of whole body, arm and leg, Trunk and leg fat-free mass, Whole body water mass, Cholesterol, Blood pressure**	**Rheumatoid arthritis, Schizophrenia, Diabetes**
BMI	CFHR2	rs4085749	None	**-**	**Neovascularization, Agx10-related**
BMI	EFEMP1	rs58680090	None	**Basal metabolic rate, Height, Trunk fat-free mass, Weigh, Whole body, arm and leg fat-free mass, Whole body water mass, Trunk fat mass, Impedance of whole body, Forced vital capacity**	**Inguinal hernia**
BMI	F13B	rs12134960	None	**SBP**	**Neovascularization, Agx10-related**
BMI	FGL1	rs3739404	-	-	-
BMI	FN1	rs1437787	EAS	-	**Coronary artery disease**
BMI	ITIH3	rs2286797	None	BMI	Myocardial infraction
BMI, WC	ITIH4	rs6803519	None	**Height, Anxiety**	**Schizophrenia**
BMI	LRP11	rs9688902	None	BMI, Hand grip strength	Hip pain
BMI	NCR3LG1	rs2051772	None	**BMI, Height, Impedance of whole body, arm and leg**	**Diabetes**
BMI	NUDT5	rs7100710	None	**BMI, Peak expiratory flow, Forced expiratory volume**	**Diabetes**
BMI	OGN	rs7023004	None	**Height, Impedance of whole body, arm and leg**	-
BMI	PRDX6	rs61826753	-	-	-
BMI	PRSS53	rs11150606	-	-	-
BMI	PTPRB	rs2465811	-	-	-
BMI	PTPRZ1	rs28379507	EAS/EUR	**Heel bone mineral density**	-
BMI	SCAMP3	rs4971072	None	**Serum magnesium, Monocytes**	**Crohn’s disease**
BMI	SERPINF2	rs8077638	EAS	**Serum albumin**	-
BMI	SLURP1	rs1036385	None	**Duodenal ulcer**	-
BMI	SUSD5	rs6764459	None	**Forced vital capacity**	-
BMI	TSPAN8	rs9683252	AFR/EAS/EUR/SAS	-	**Asthma**
BMI	TXNDC15	rs3733897	None	**Height**	-

[a]Traits or diseases in bold: P < 5*10-8 , others were P < 5*10-6; BMI: body mass index; HC: hip circumference; SBP: systolic blood pressure; WC: waist circumference

Supplementary Table 10. Number of proteins with QC warnings

Number of QC warnings per protein, n (%)[1]	Number of proteins, n (%)[2]
9 (0.2%)	108 (3.7%)
10 (0.3%)	51 (1.7%)
13 (0.3%)	148 (5.1%)
15 (0.4%)	145 (5.0%)
20 (0.5%)	62 (2.1%)
22 (0.6%)	93 (3.2%)
23 (0.6%)	62 (2.1%)
25 (0.6%)	115 (3.9%)
31 (0.8%)	85 (2.9%)
32 (0.8%)	76 (2.6%)
34 (0.9%)	62 (2.1%)
36 (0.9%)	89 (3.0%)
37 (0.9%)	92 (3.1%)
45 (1.1%)	181 (6.2%)
46 (1.2%)	98 (3.4%)
53 (1.3%)	109 (3.7%)
58 (1.5%)	113 (3.9%)
79 (2.0%)	93 (3.2%)
90 (2.3%)	109 (3.7%)
105 (2.6%)	96 (3.3%)
113 (2.8%)	144 (4.9%)
115 (2.9%)	103 (3.5%)
121 (3.0%)	143 (4.9%)
123 (3.1%)	92 (3.1%)
127 (3.2%)	88 (3.0%)
139 (3.5%)	79 (2.7%)
141 (3.5%)	69 (2.4%)
148 (3.7%)	109 (3.7%)
158 (4.0%)	105 (3.6%)

[1] A total of 3977 samples
[2] A total of 2923 proteins

Supplementary Table 11. Variance explained by adiposity genetic scores in CKB and UKB

Adiposity measures	CKB			UKB		
	**Men** (n=1840)	**Women** (n=2137)	**All** (n=3977)	**Men** (n=15,353)	**Women** (n=19,094)	**All** (n=34,447)
BMI	2.4	4.8	3.7	8.5	8.0	8.1
BF%*	2.7	3.9	1.9	7.3	6.4	3.5
WC	1.6	3.6	2.5	5.9	5.6	4.7
WHR	1.0	2.4	1.5	2.7	6.9	3.0

* In CKB, for BF% the analyses were performed for 3973 participants (1838 men and 2135 women)

Abbreviations: BF%=body fat percentage; BMI=body mass index; CKB=China Kadoorie Biobank; UKB=UK Biobank; WC=waist circumference; WHR=waist-to-hip ratio

Supplementary Table 12. Associations of potential confounders with BMI and WC GS

Confounder	OR or β (95% CI) per 1-SD higher genetically-instrumented	
	BMI	WC
Ever-regular smoker	1.08 (0.97, 1.20)	1.09 (0.98,1.22)
Ever-regular alcohol drinker	0.96 (0.86, 1.06)	0.99 (0.90, 1.10)
Physical activity	0.94 (0.85, 1.03)	0.97 (0.88, 1.06)
Education	1.01 (0.92, 1.10)	1.02 (0.94, 1.10)
Income	1.02 (0.95, 1.10)	1.01 (0.94, 1.08)
Standing height, cm	0.02 (-0.17, 0.17)	0.22 (0.04, 0.39)
RPG, mmol/L	0.21 (0.11, 0.31)	0.23 (0.14, 0.33)
SBP, mmHg	1.23 (0.55, 1.92)	0.42 (-0.27, 1.12)
BMI, kg/m^2	-	0.57 (0.46, 0.67)
Waist circumference, cm	1.57 (1.28, 1.86)	-

The model was adjusted for age at baseline, age squared, sex, 10 regions, and 11 PCs. Potential confounders were dichotomised: ever-regular smoker (yes vs no), ever-regular alcohol drinker (yes vs no), total PA (≥30 vs <30 MET-h/day), education (≥9 vs <9 years), and household income (≥10,000 vs <10,000 RMB/year). The analyses of smoking and alcohol were conducted in men due to the small number of women who smoked (4%) or drank (2%).
Abbreviations: BMI=body mass index; OR=odds ratio; RPG=random plasma glucose; SBP=systolic blood pressure; WC=waist circumference

Supplementary Figure 1. Correlation between adiposity measures and genetic scores

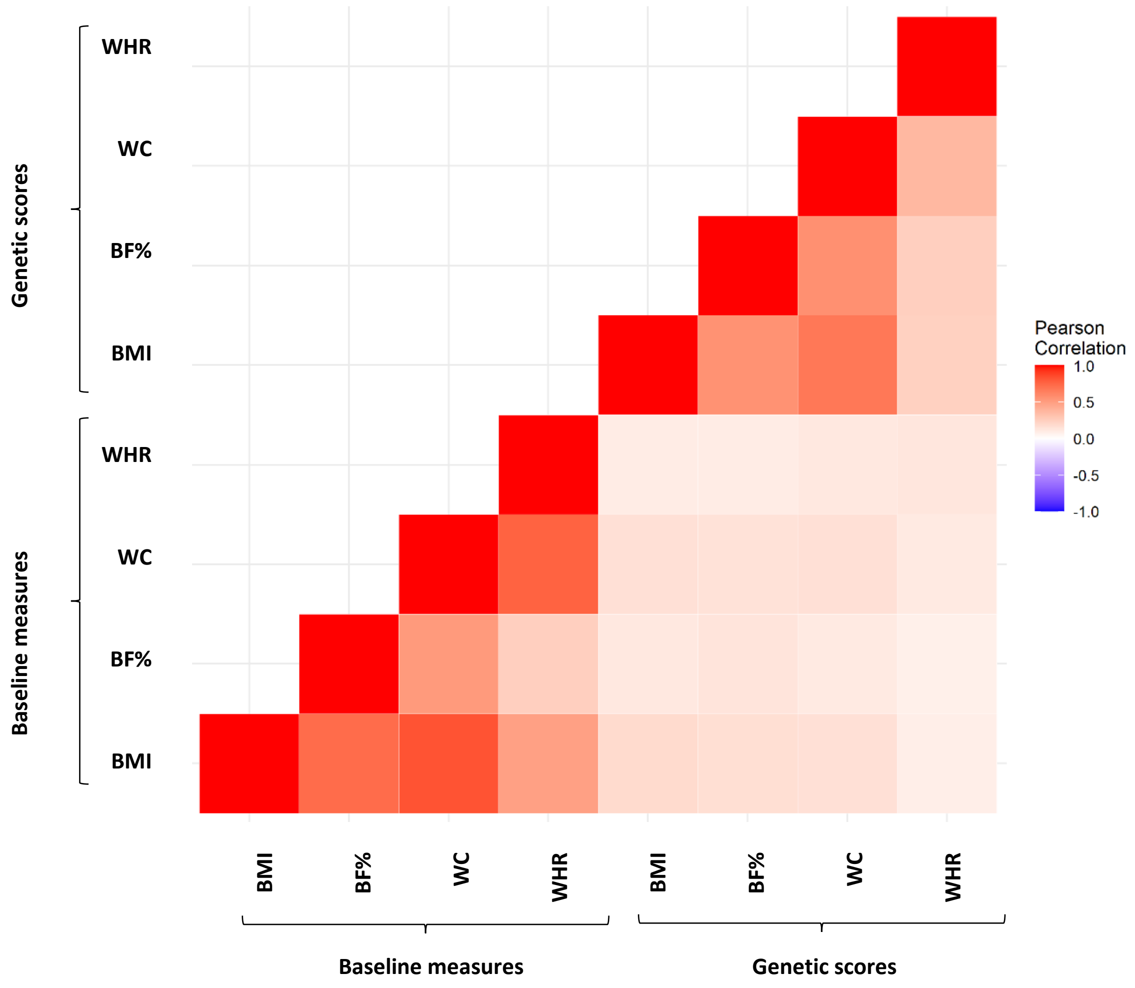

Supplementary Figure 2. Number of proteins significantly associated with adiposity in conventional and genetic analyses

a) FDR<0.05

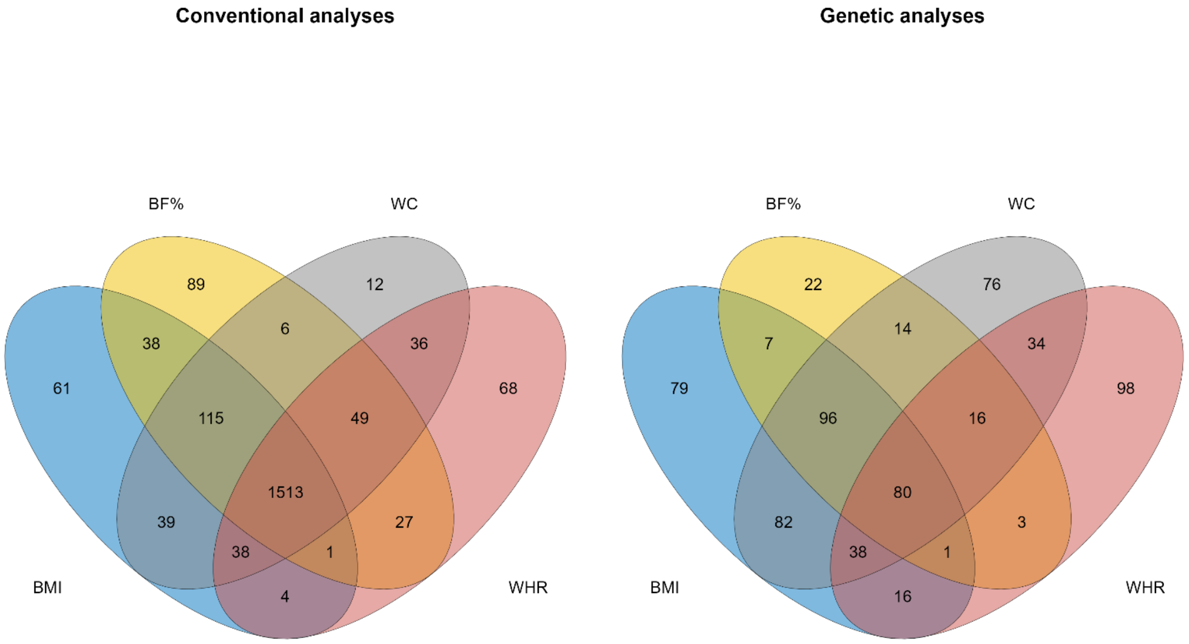

b) Bonferroni<0.05

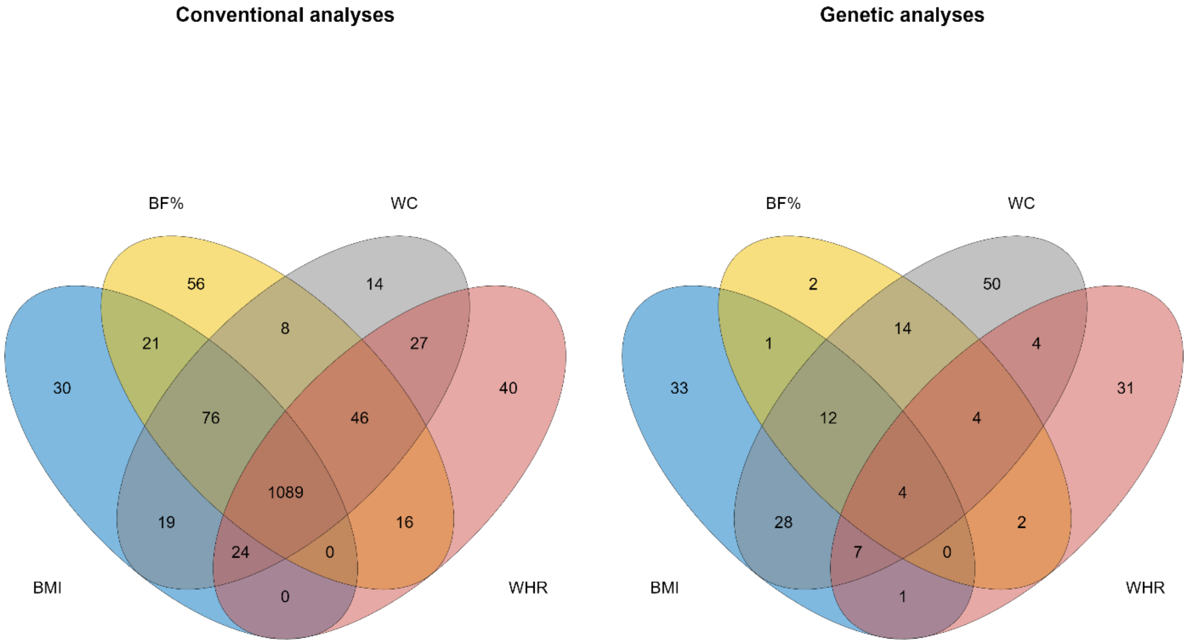

Supplementary Figure 3. Global comparison of associations of protein biomarkers with 1-SD higher BMI, BF%, WC and WHR in conventional and genetic analyses in OLINK batch 1 and 2

Black dots denote Olink batch 1, while red dots denote batch 2 and grey lines denote the 95% confidence intervals. Analyses were adjusted for age, age^2, sex, study area, fasting time, ambient temperature, ascertainment status, and the first 11 PCs (for genetic analyses only).

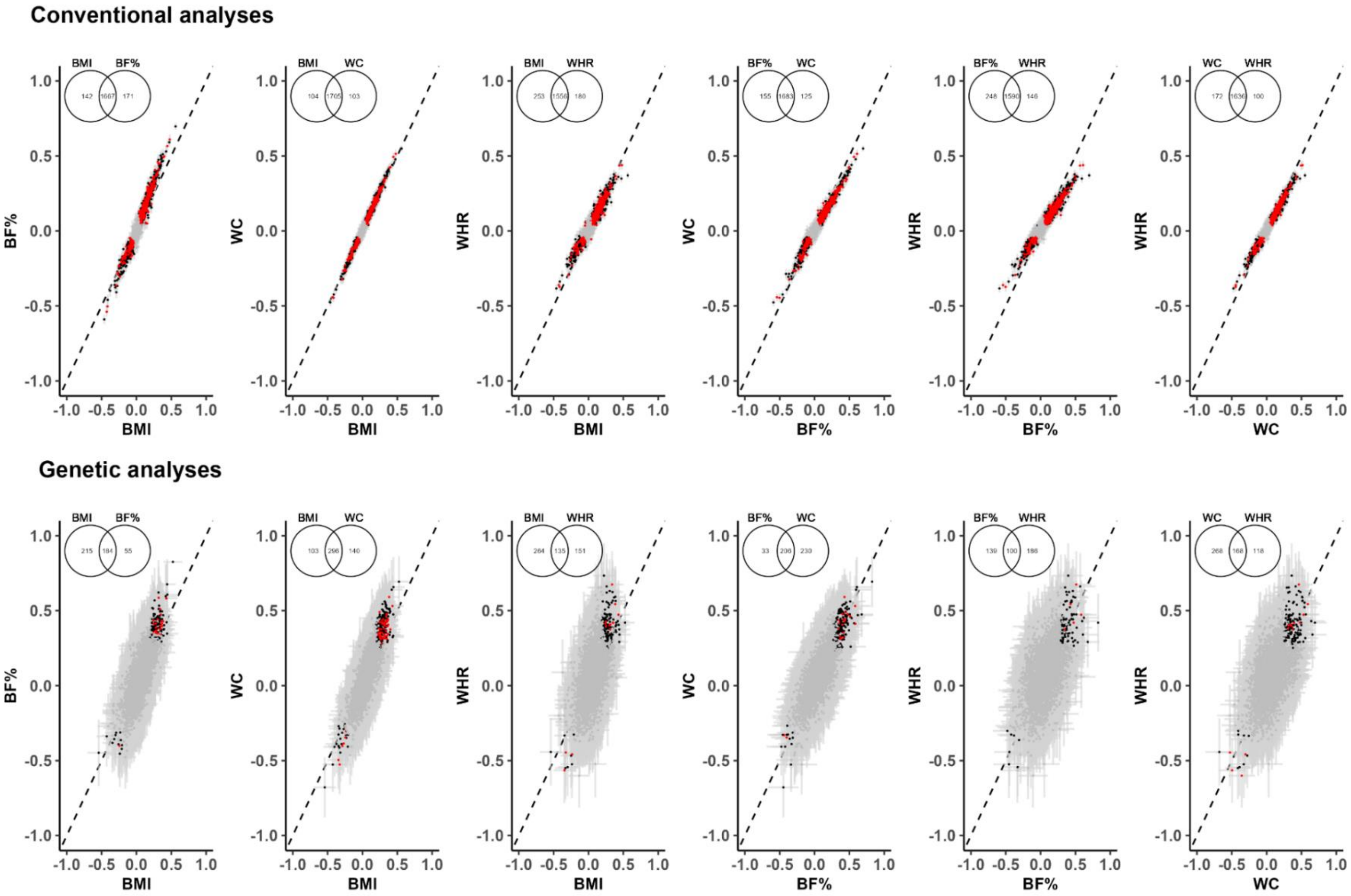

Supplementary Figure 4. Number of proteins significantly associated with adiposity in conventional and genetic analyses after mutual adjustments

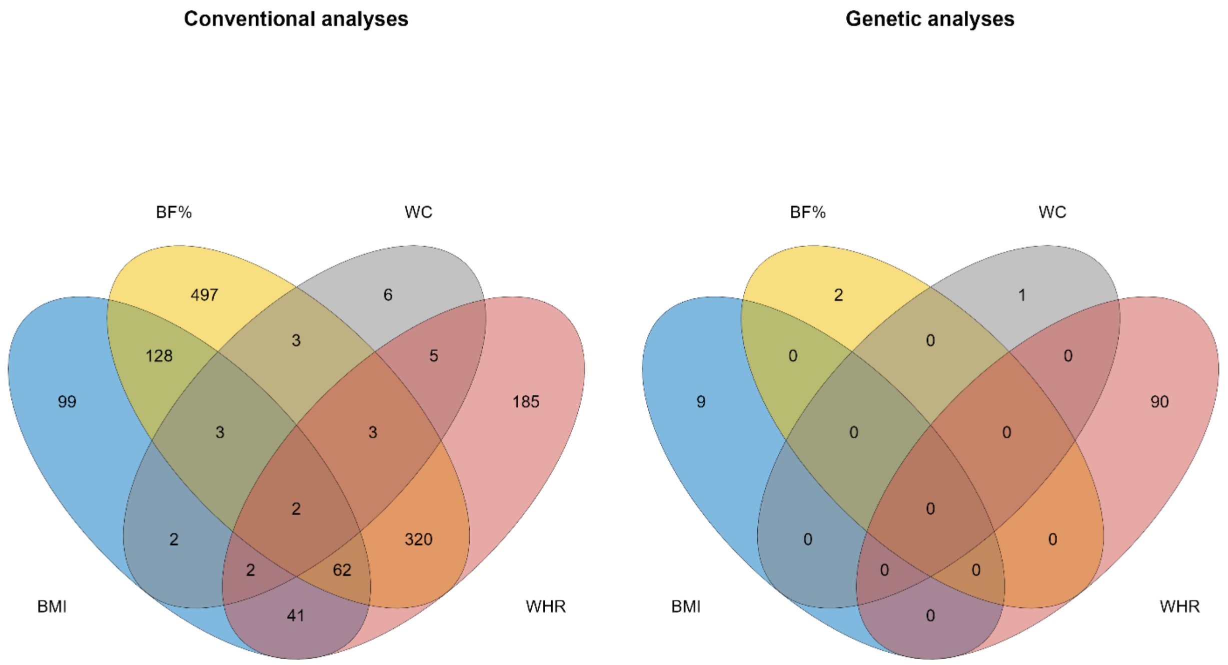

Supplementary Figure 5. Global comparison of associations of protein biomarkers with 1-SD higher BMI, BF%, WC and WHR in conventional and genetic analyses in OLINK batch 1 and 2, by sex

Black dots denote Olink batch 1, while red dots denote batch 2 and grey lines denote the 95% confidence intervals. Analyses were adjusted for age, age^2, study area, fasting time, ambient temperature, ascertainment status, and the first 11 PCs (for genetic analyses only).

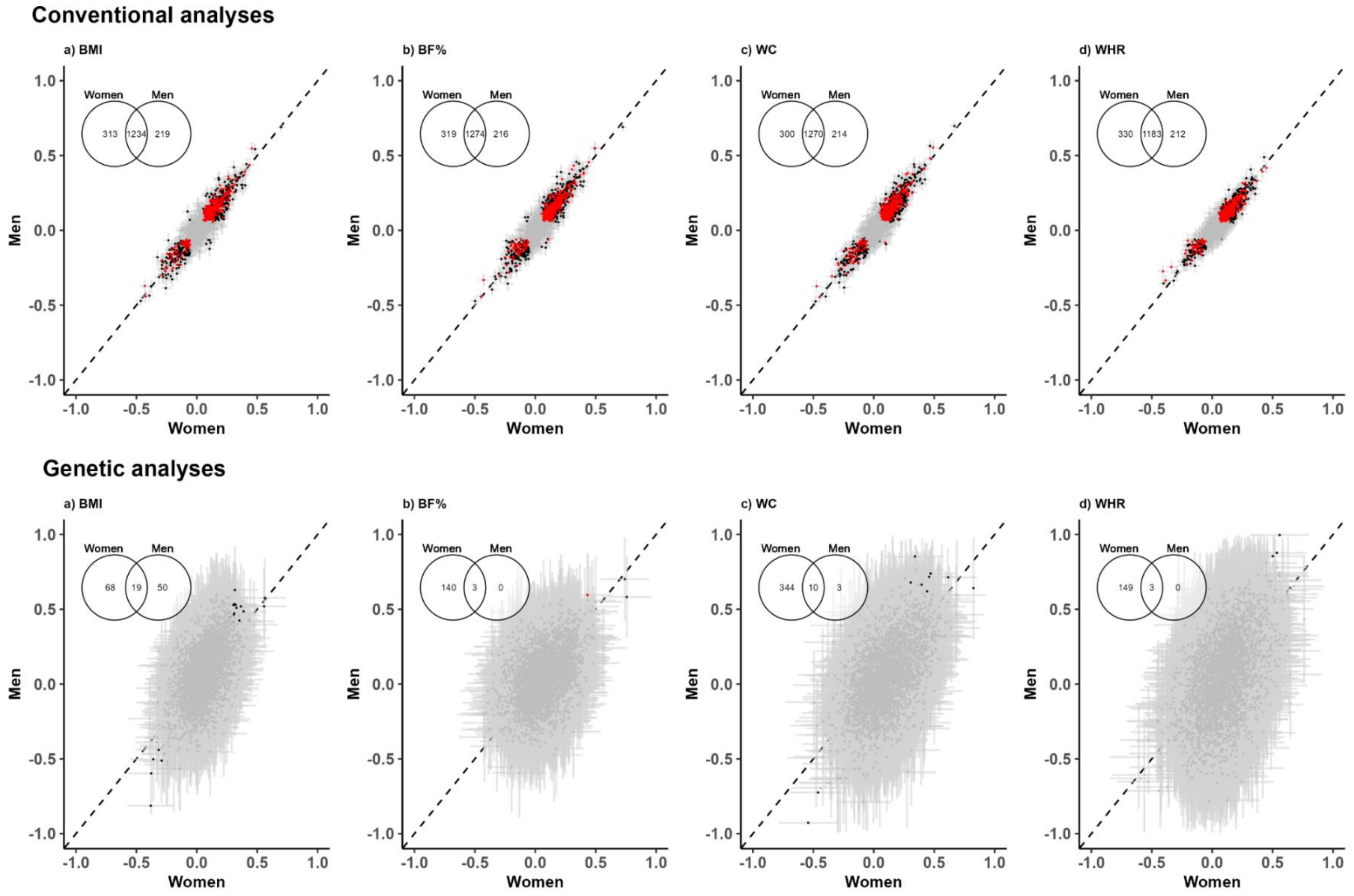

Supplementary Figure 6. Volcano plot of associations of proteins with 1-SD higher BMI, BF%, WC and WHR in conventional and genetic analyses in OLINK batch 1 and 2 in the subcohort

Solid dots denote Olink batch 1, while open dots denote batch 2. Analyses were adjusted for age, age^2, sex, study area, fasting time, ambient temperature, plate ID, ascertainment status, and the first 11 PCs (for genetic analyses only).

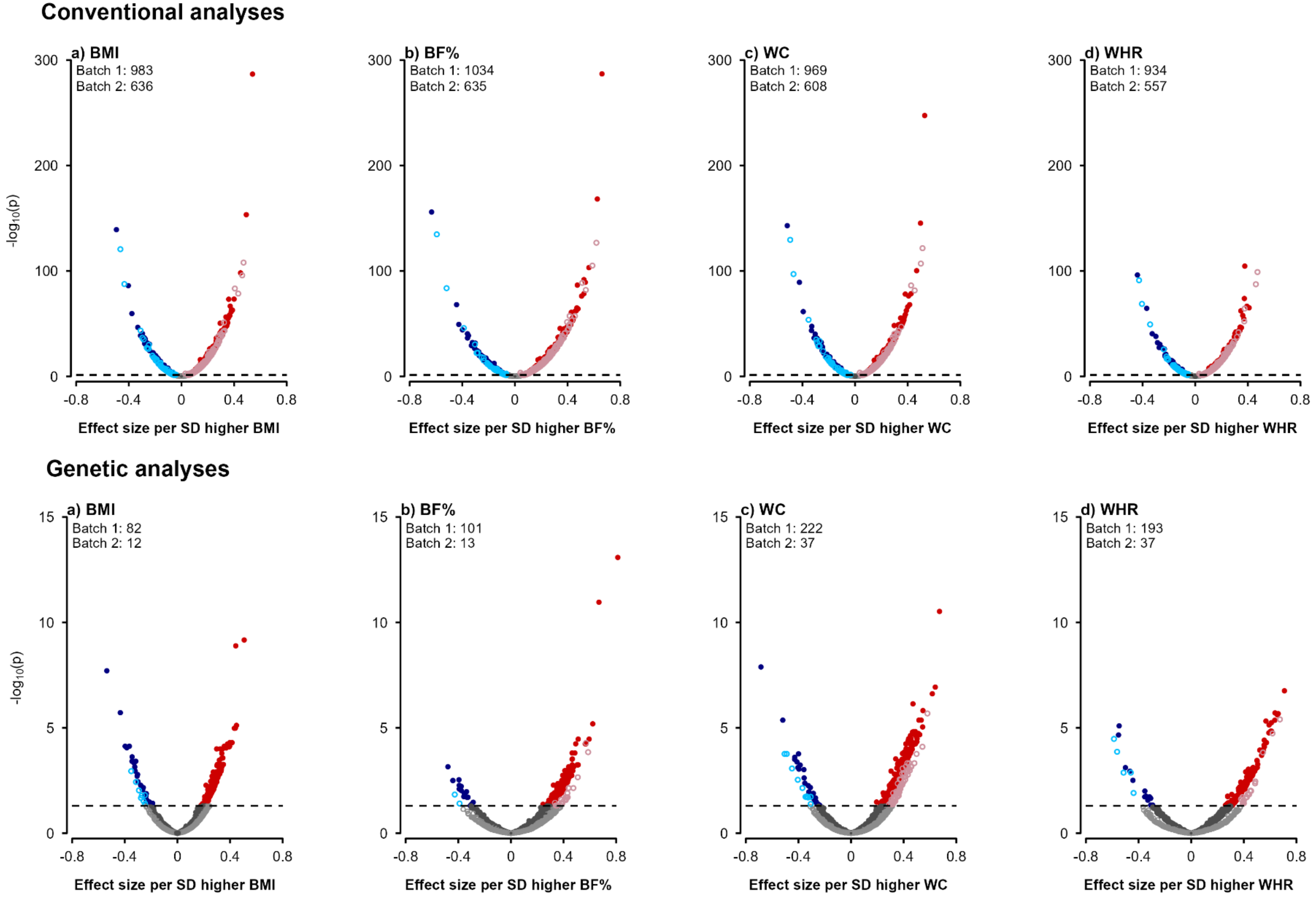

Supplementary Figure 7. Comparison of associations of protein biomarkers with 1-SD higher BMI in genetic analyses in CKB and CKB vs. UKB using different CKB BMI GS

Black dots denote Olink batch 1, while red dots denote batch 2 and grey lines denote the 95% confidence intervals. In CKB, analyses were adjusted for age, age^2, sex, study area, fasting time, ambient temperature, ascertainment status, and the first 11 PCs. In UKB, analyses were adjusted for age, age^2, sex, assessment centre, fasting time, plate ID, and the first 40 PCs.

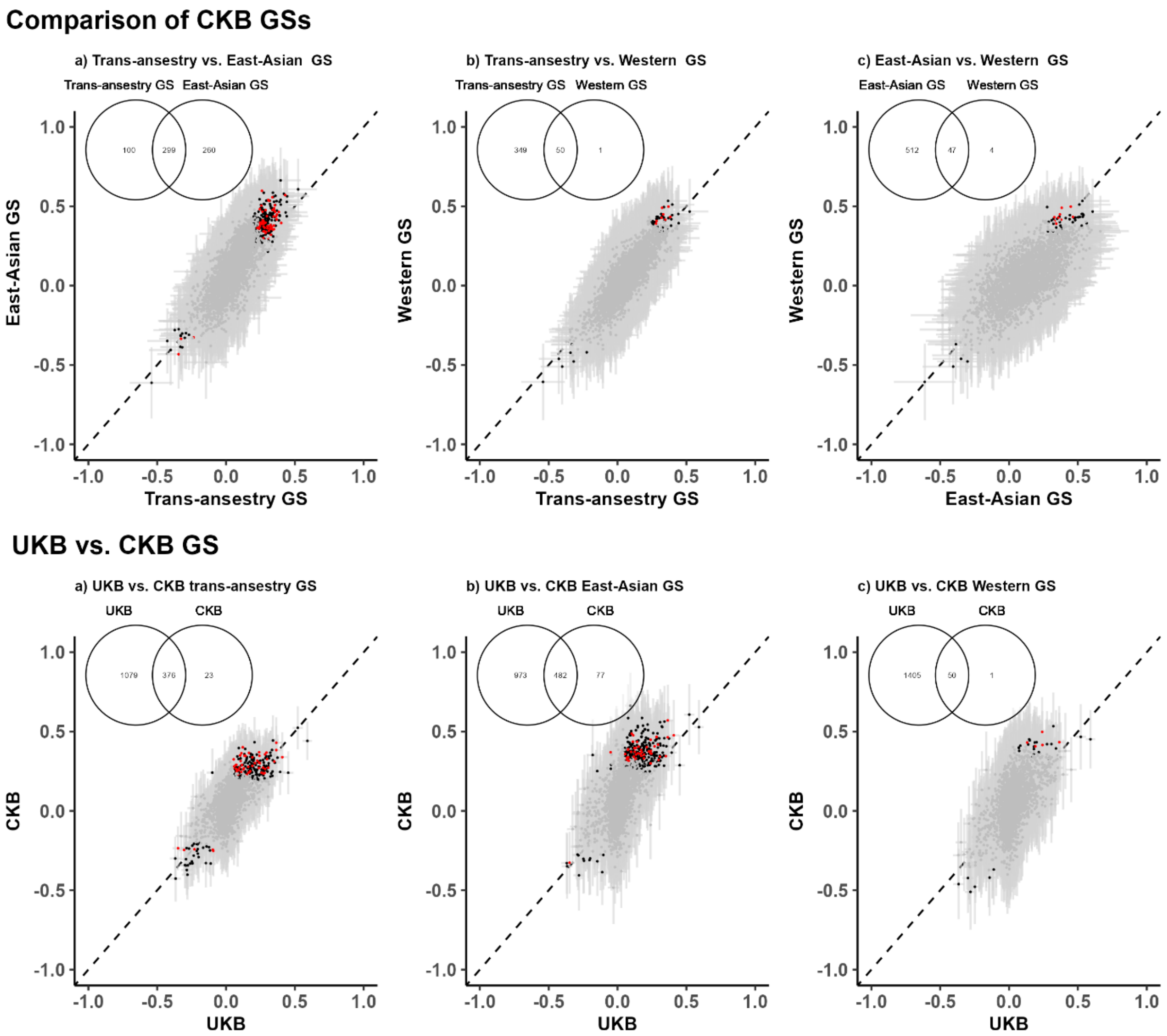

Supplementary Figure 8. Comparison of associations of protein biomarkers with 1-SD higher BMI, BF%, WC and WHR in conventional and genetic analyses in CKB vs. UKB, in OLINK batch 1 and 2

Black dots denote Olink batch 1, while red dots denote batch 2 and grey lines denote the 95% confidence intervals. In CKB, analyses were adjusted for age, age^2, sex, study area, fasting time, ambient temperature, ascertainment status, and the first 11 PCs (for genetic analyses only). In UKB, analyses were adjusted for age, age^2, sex, assessment centre, fasting time, plate ID, and the first 40 PCs (for genetic analyses only).

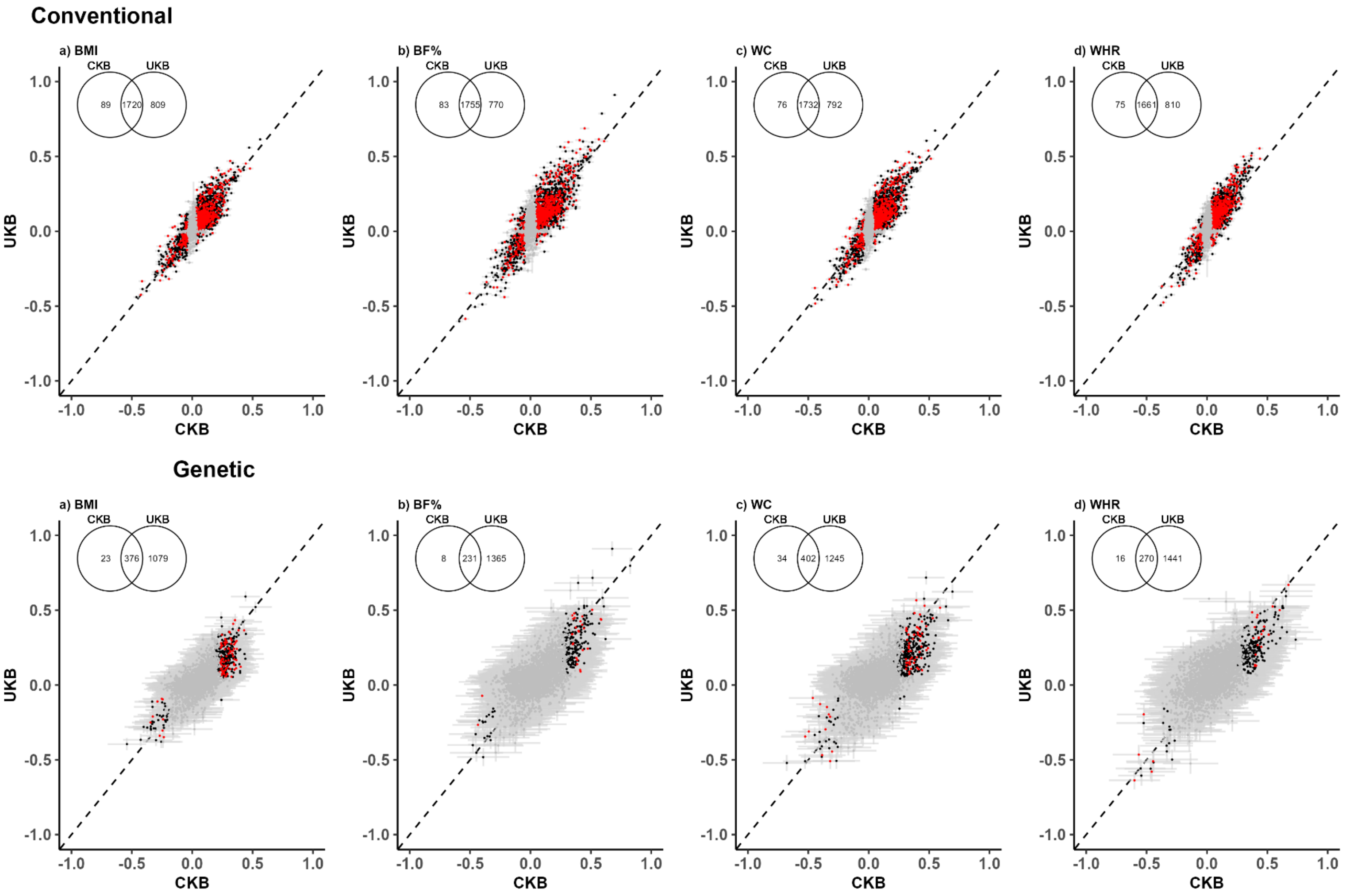

Supplementary Figure 9. Correlation matrix between the 31 proteins showing causal relationships with adiposity

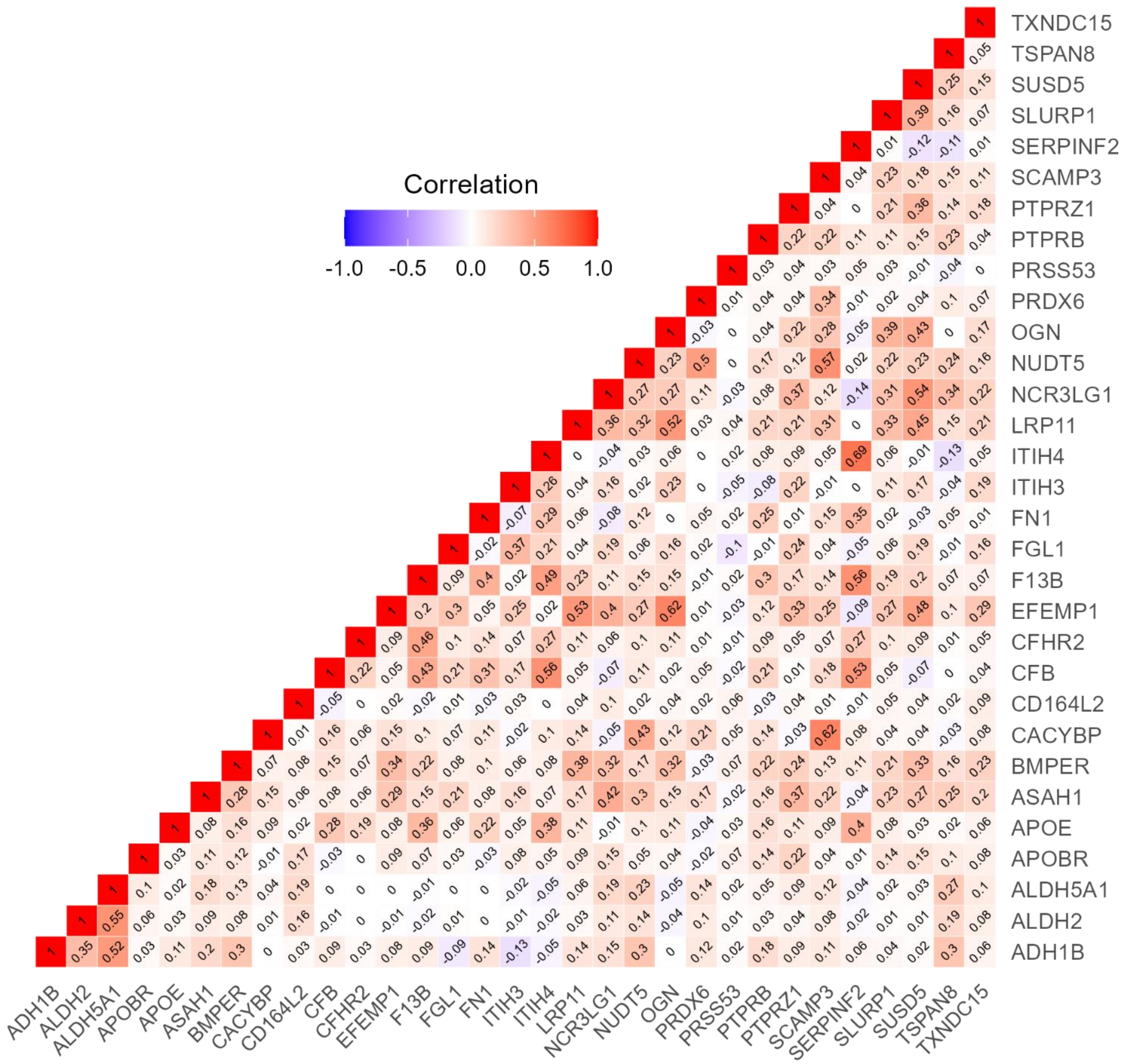

Supplementary Figure 10. Locus-zoom plots of colocalisation analyses

a) **BMI and ADH1B**

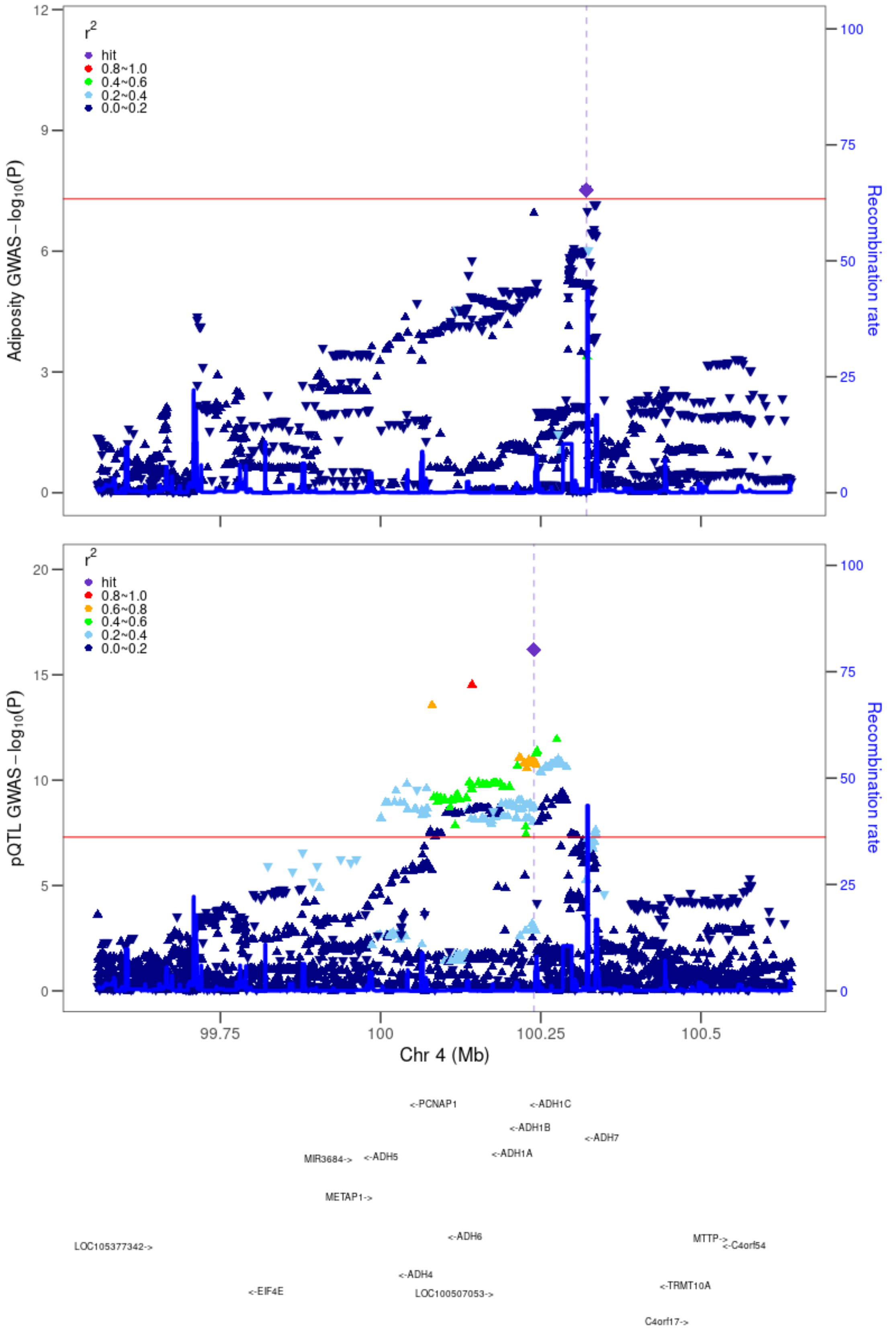

b) BMI and APOE

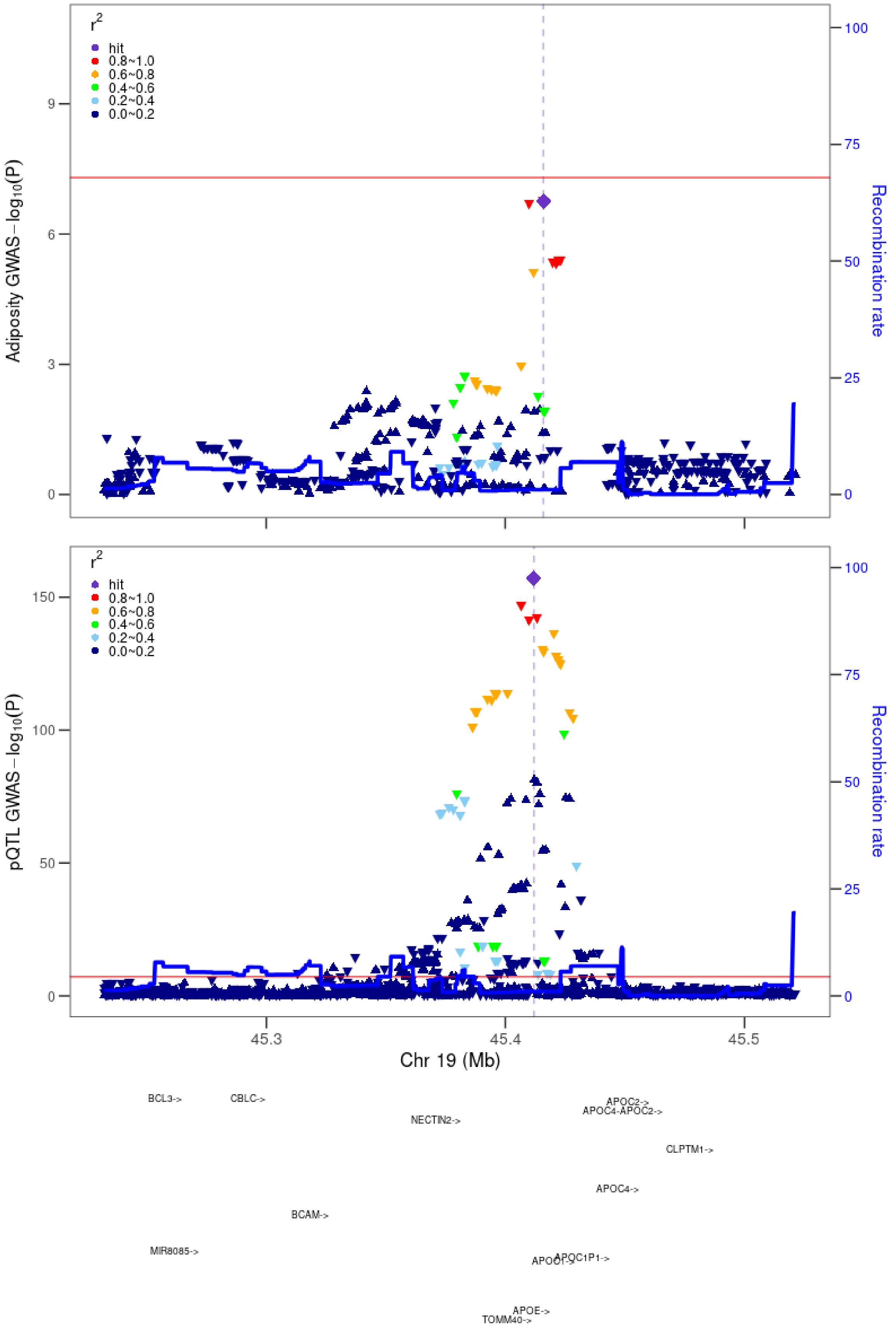

c) BMI and FN1

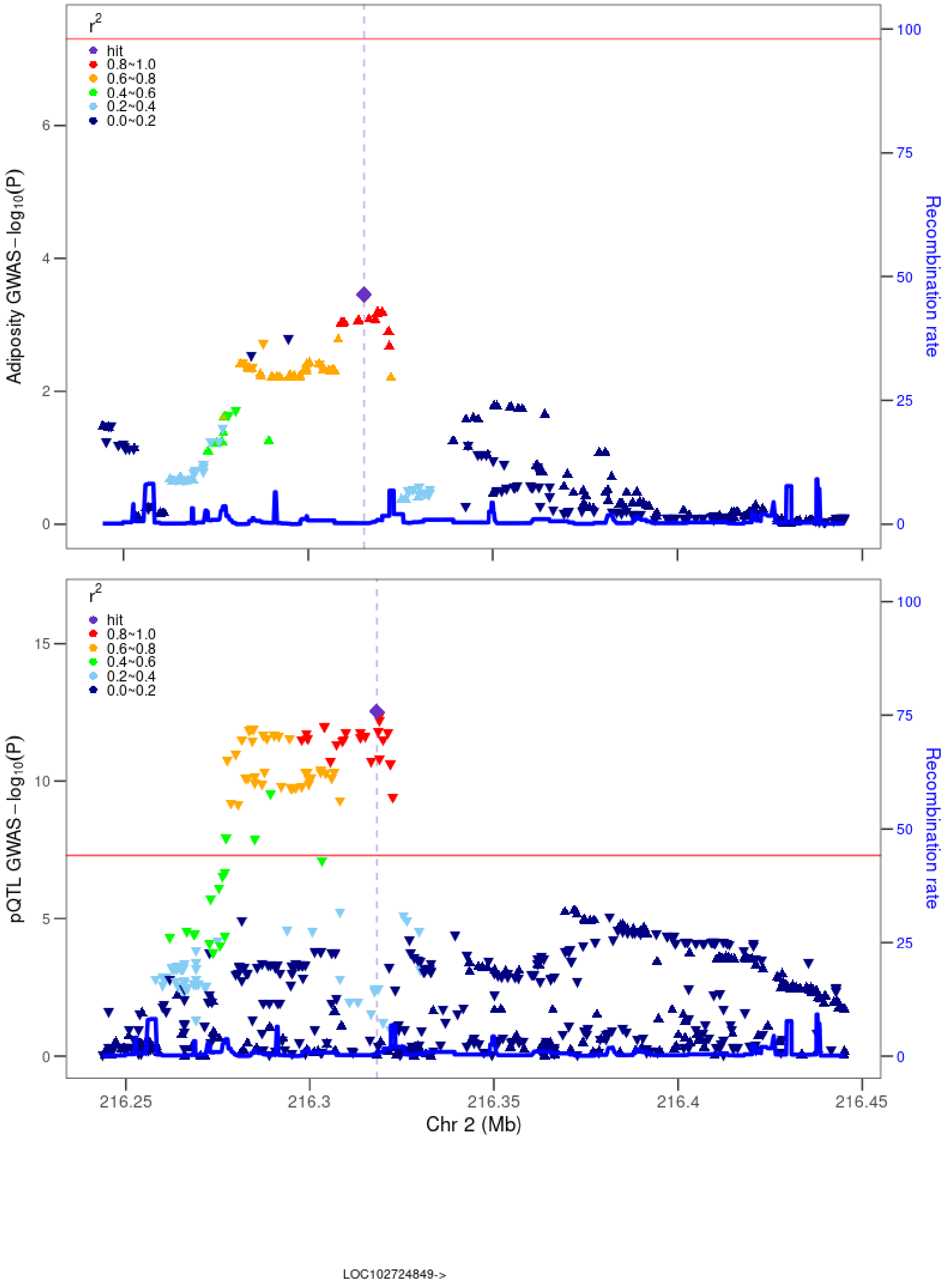

r2
hit
0.8~1.0
0.6~0.8
0.4~0.6
0.2~0.4
0.0~0.2
Adiposity GWAS − log10(P)
pQTL GWAS − log10(P)
Recombination rate
216.25
216.3
216.35
216.4
216.45
Chr 2 (Mb)
LOC102724849->

d) BMI and LRP11

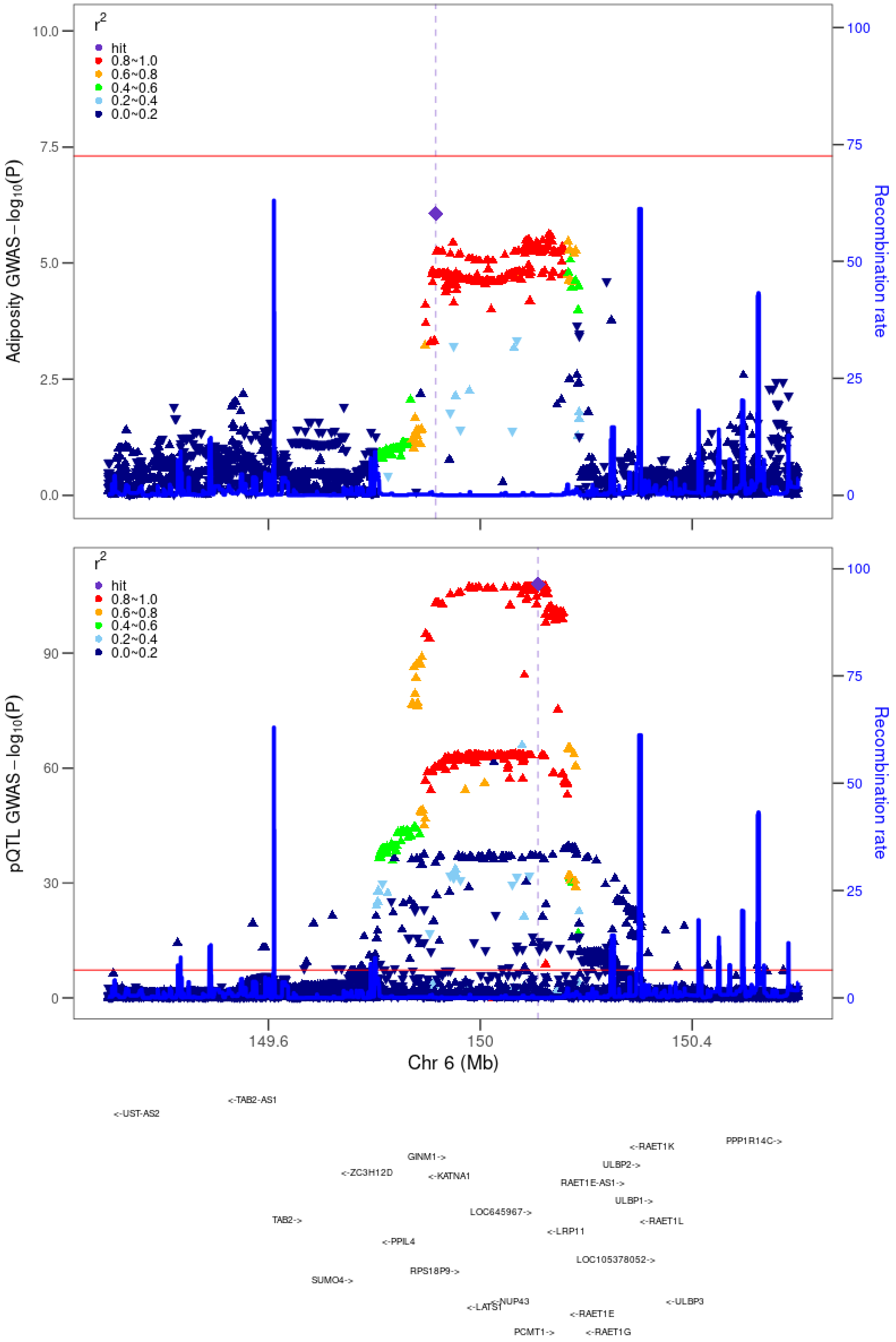

e) BMI and NCR3LG1

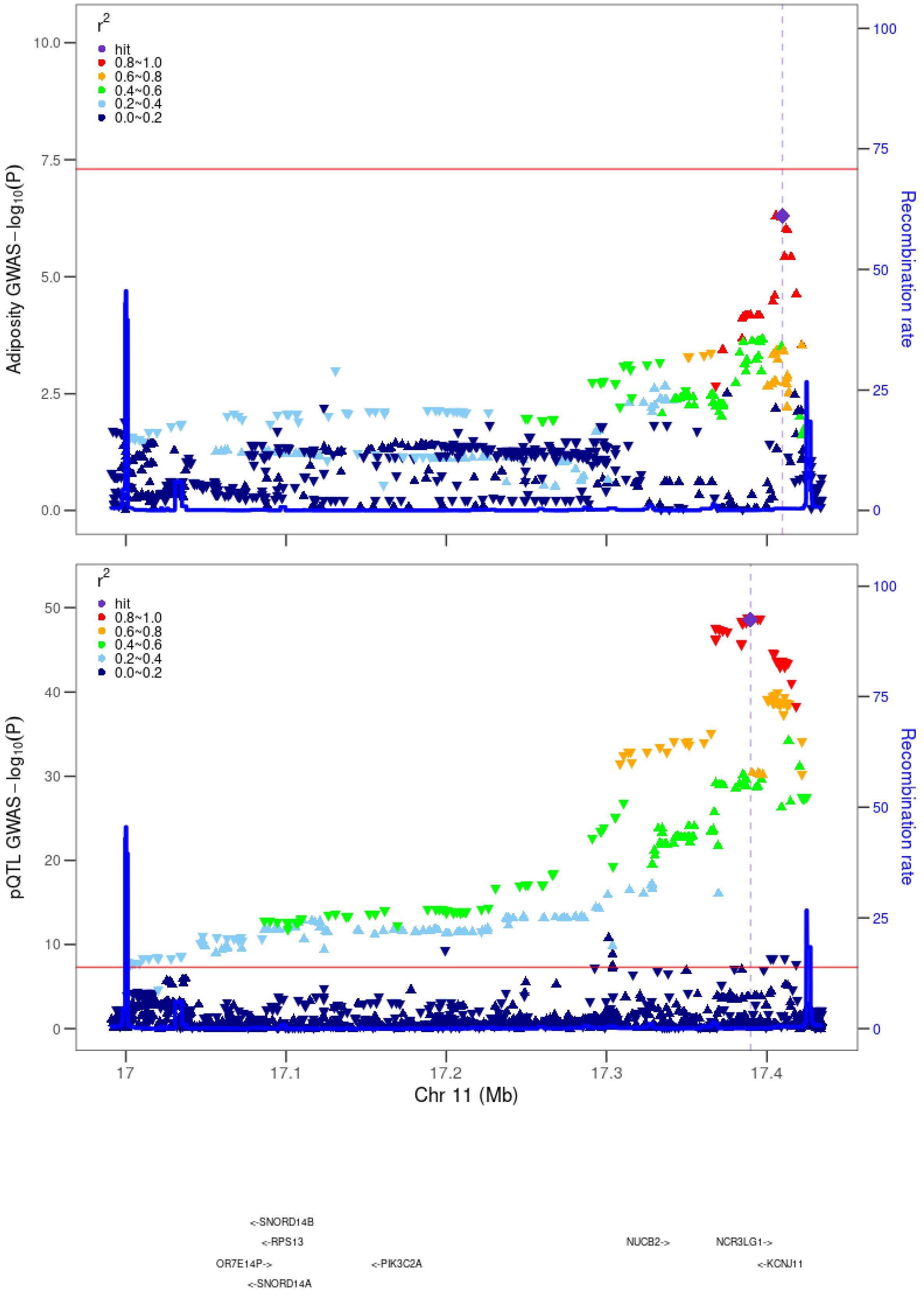

f) BMI and OGN

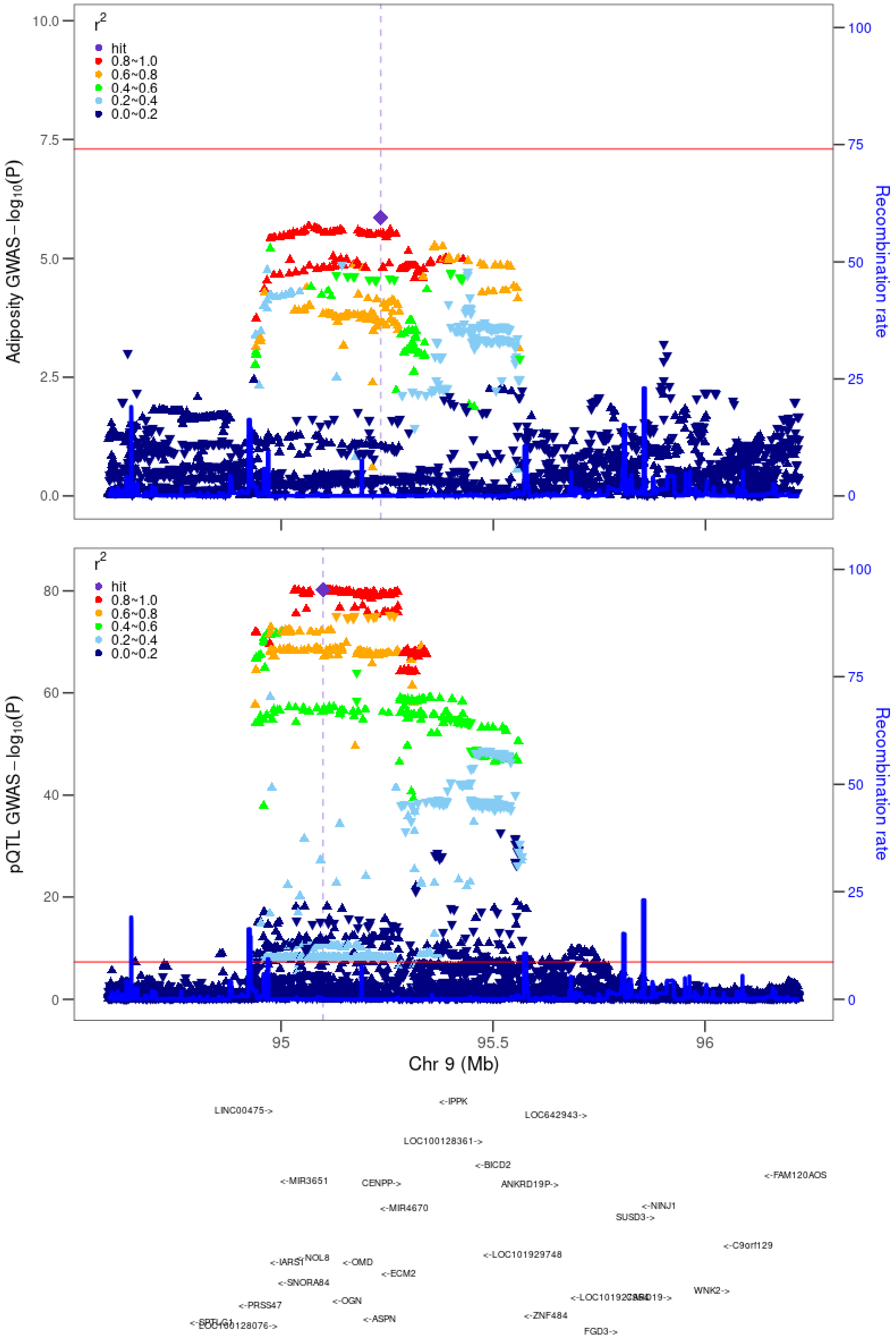

Adiposity GWAS − log10(P)
Recombination rate
r2
hit
0.8~1.0
0.6~0.8
0.4~0.6
0.2~0.4
0.0~0.2
pQTL GWAS − log10(P)
95
95.5
96
Chr 9 (Mb)
LINC00475->
<-IPPK
LOC642943->
LOC100128361->
<-BICD2
<-MIR3651
CENPP->
ANKRD19P->
<-FAM120AOS
<-MIR4670
<-NINJ1
SUSD3->
<-C9orf129
<-IARS
<-NOL8
<-OMD
<-LOC101929748
<-ECM2
<-SNORA84
<-PRSS47
<-OGN
WNK2->
<-ZNF484
<-ASPN
FGD3->

g) BMI and SERPINF2

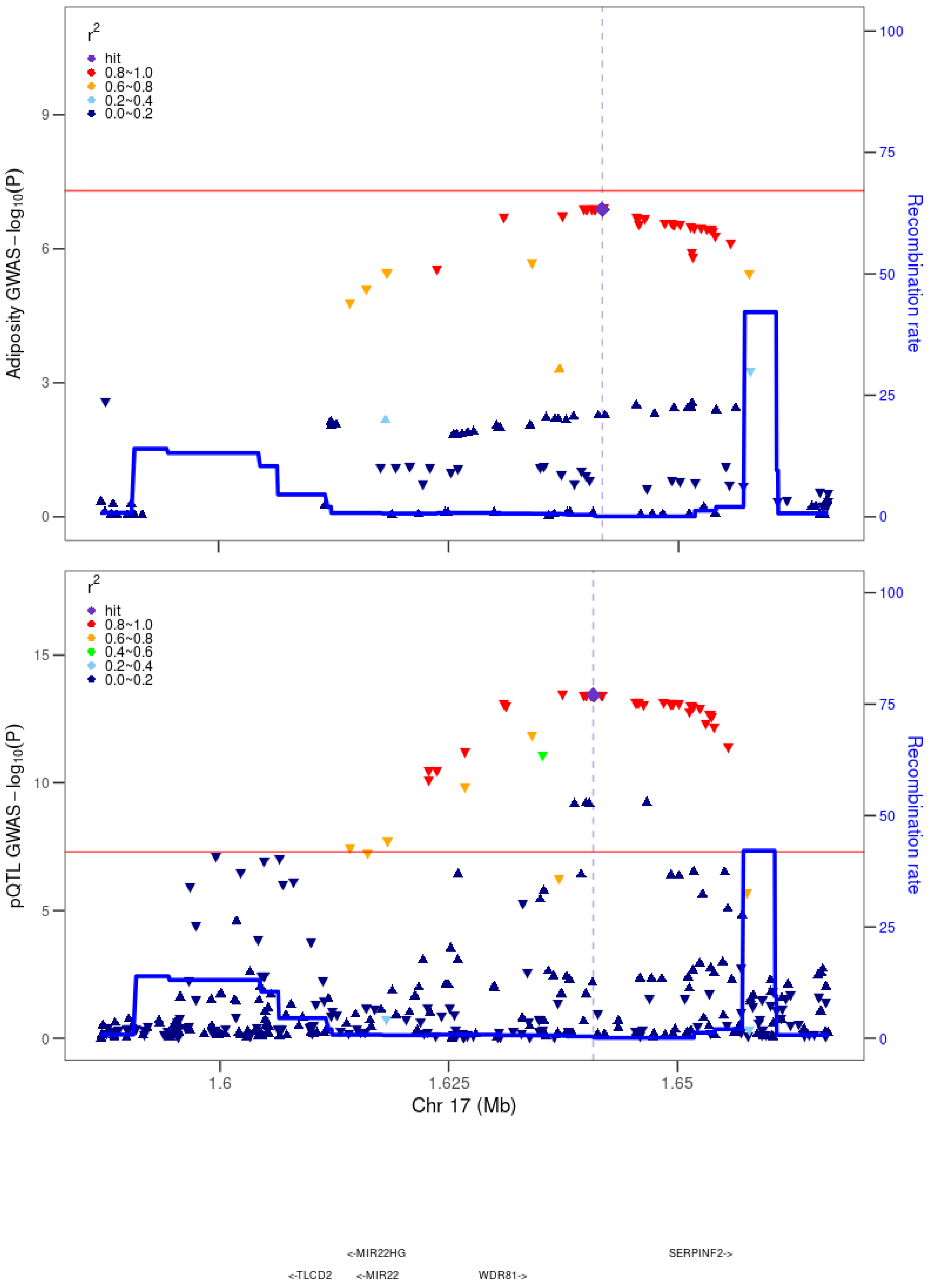

h) BMI and TSPAN8

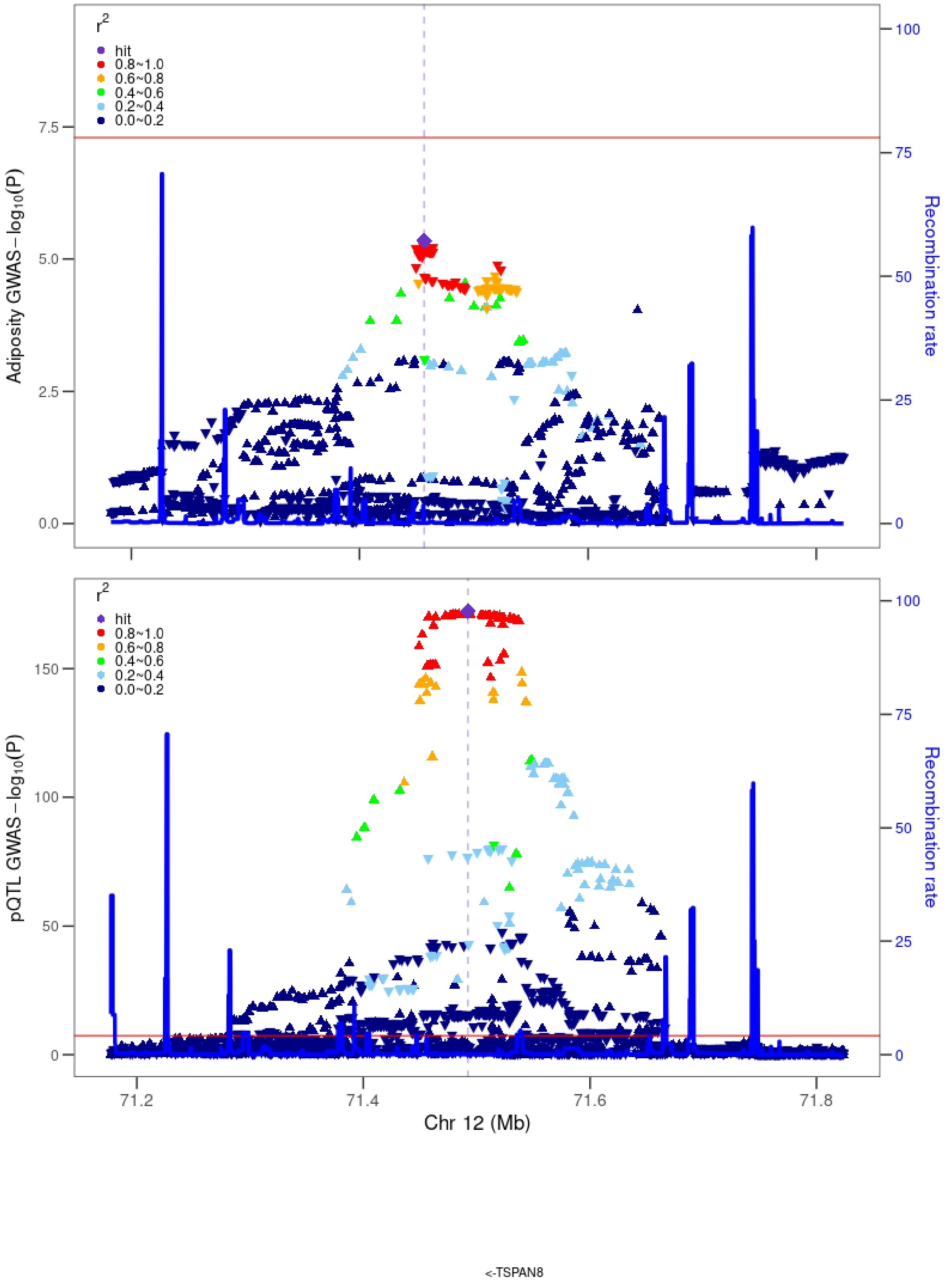

i) WC and ALDH2

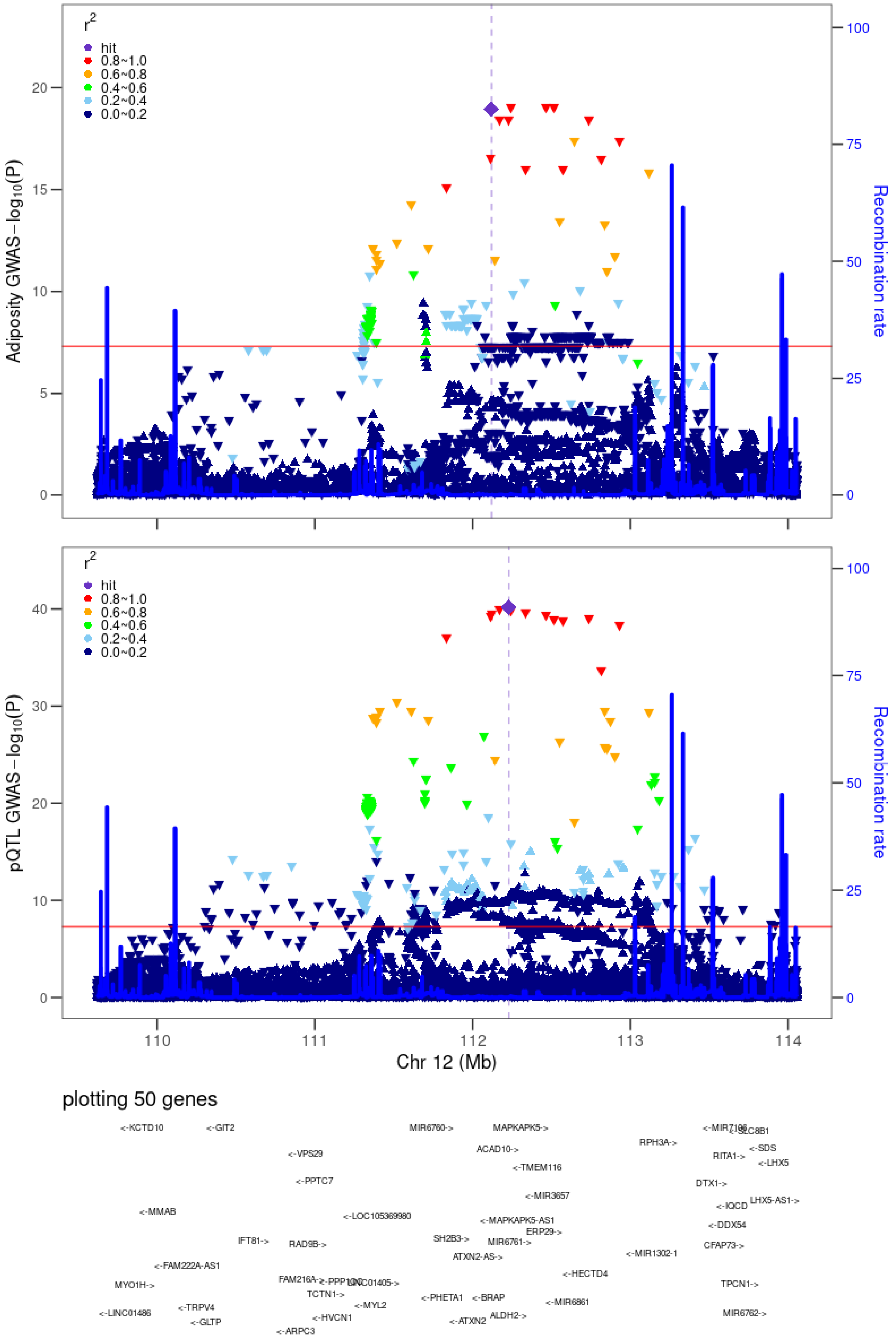

j) WC and ITIH4

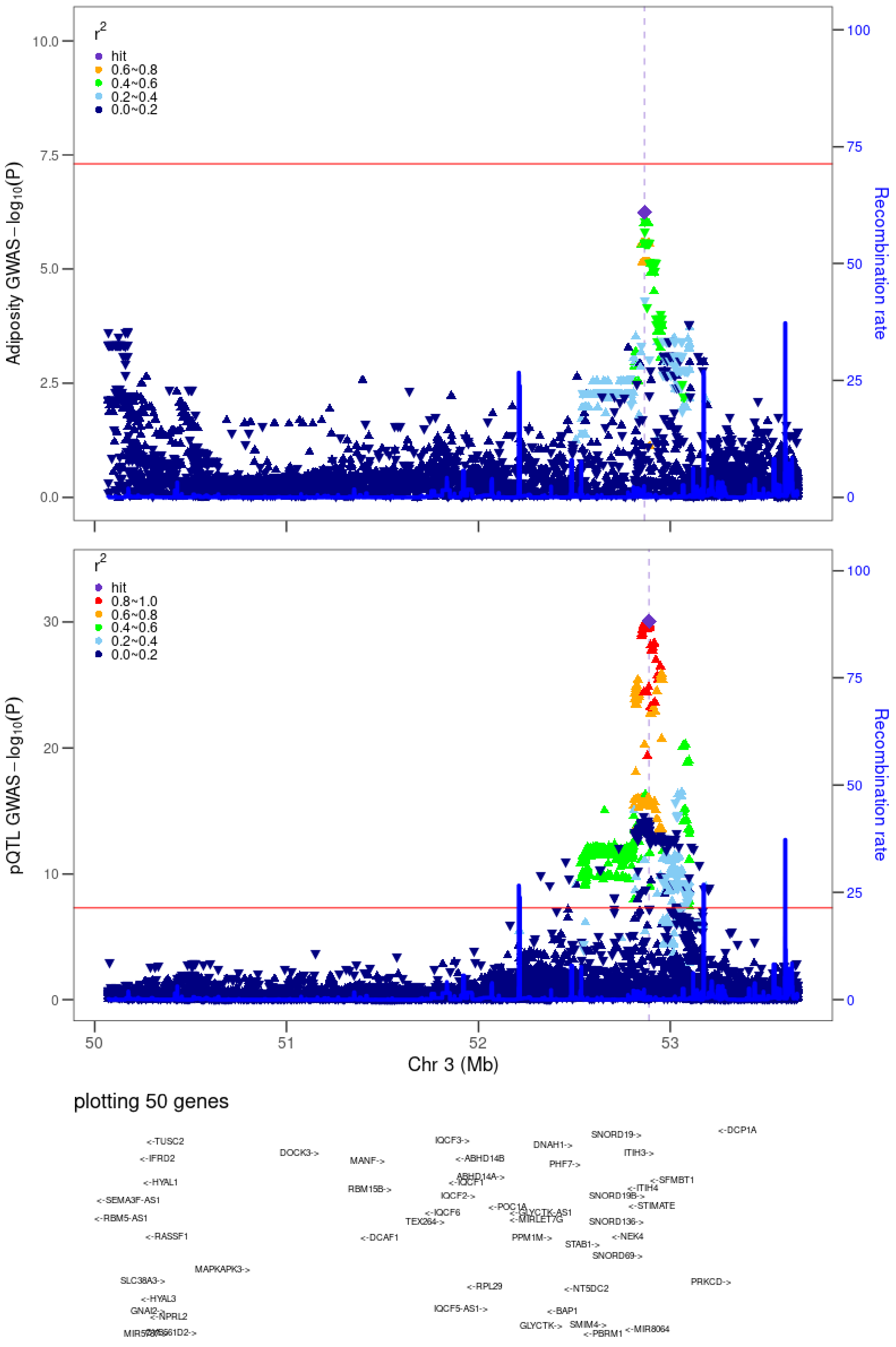

Supplementary Figure 11. Human tissux10-specific gene expression for the 31 proteins showing causal relationships with adiposity
Human RNA-seq data from GTEx showing the transcript per million (TPM) expression values for the genes encoding the proteins

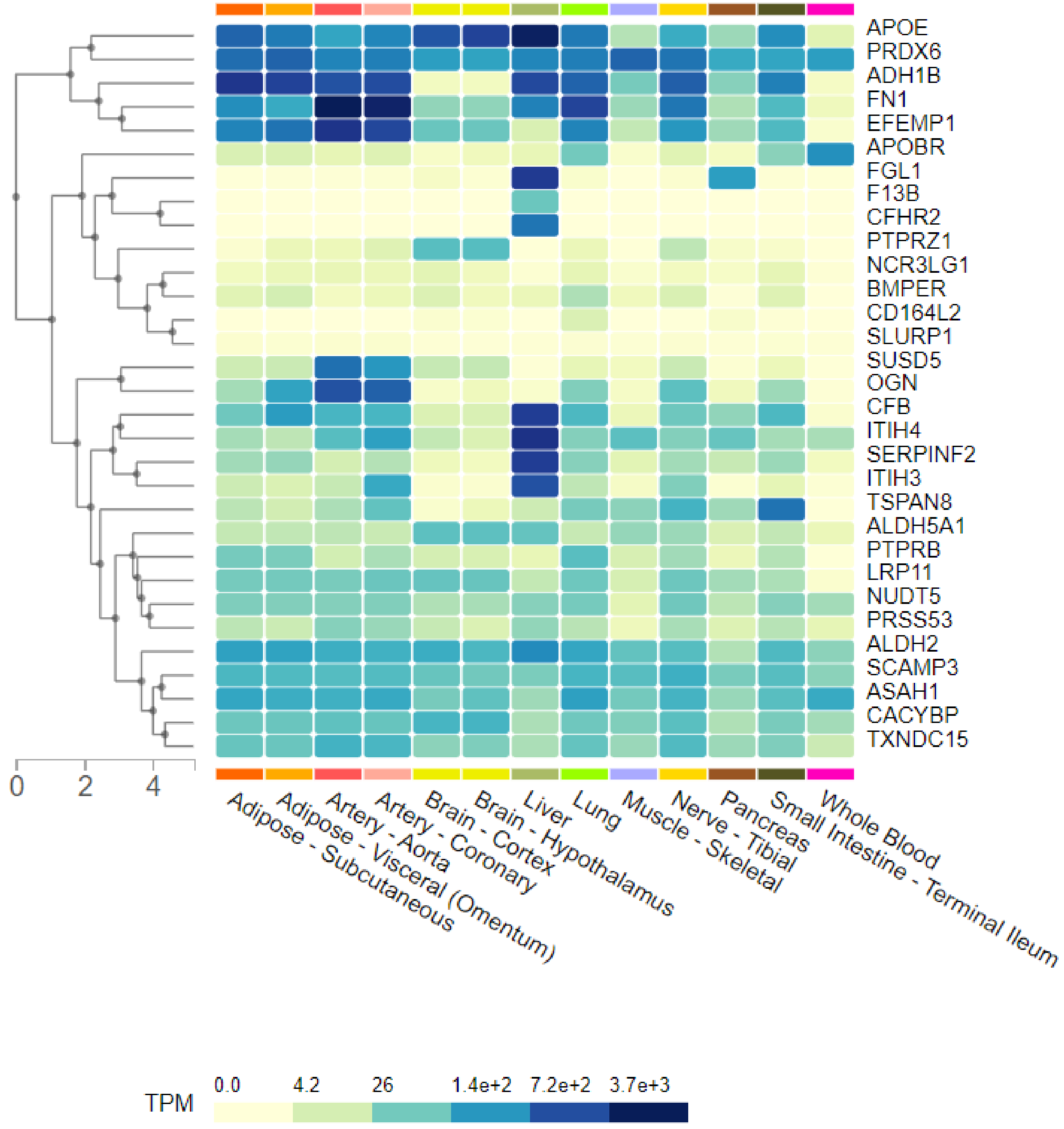

Supplementary Figure 12. Flow diagram of study design and participant selection in CKB

*Selection for the subcohort used simple random sampling; †Individuals may be included in more than one study arm

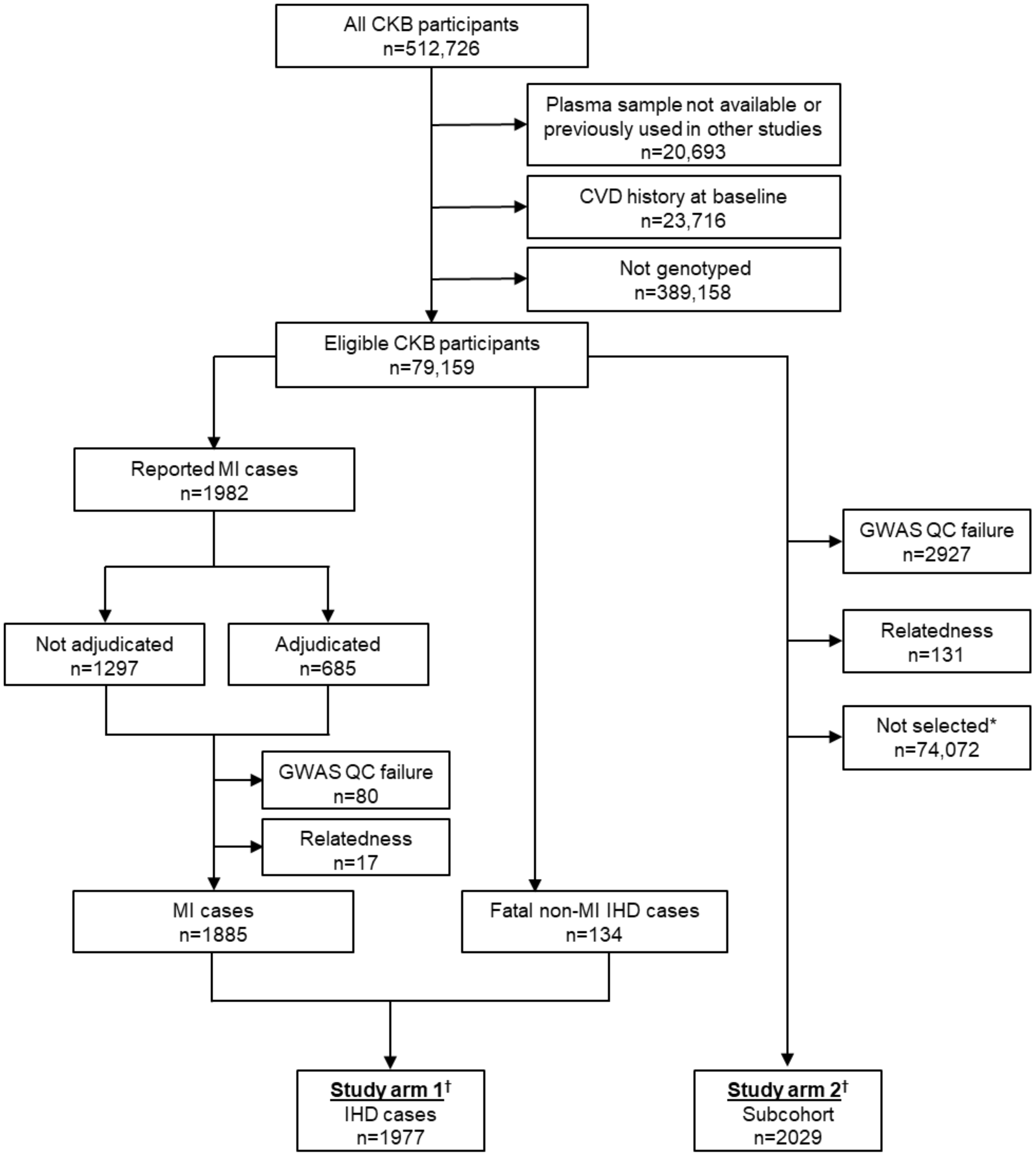

Supplementary Figure 13. Distribution of proteins

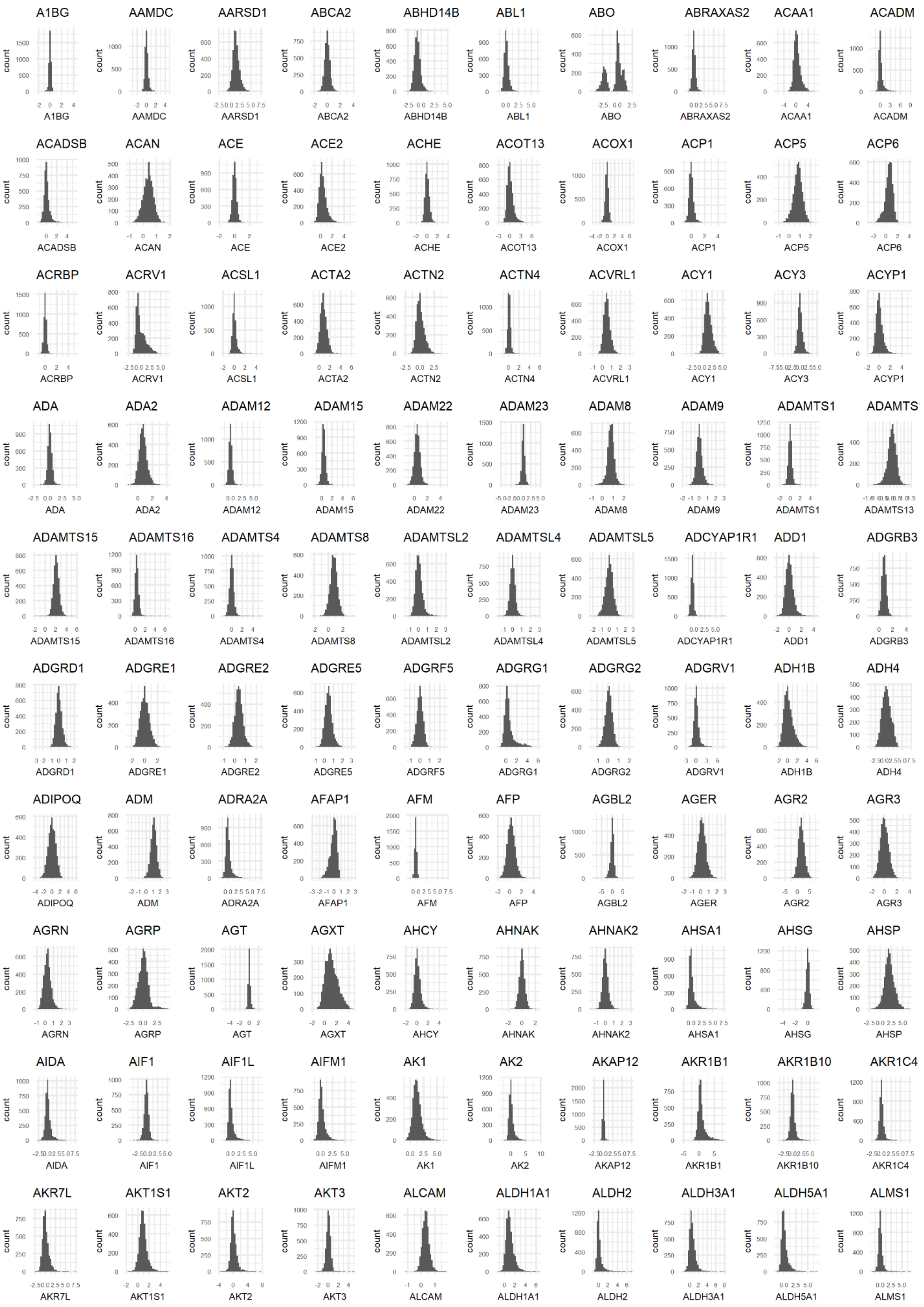

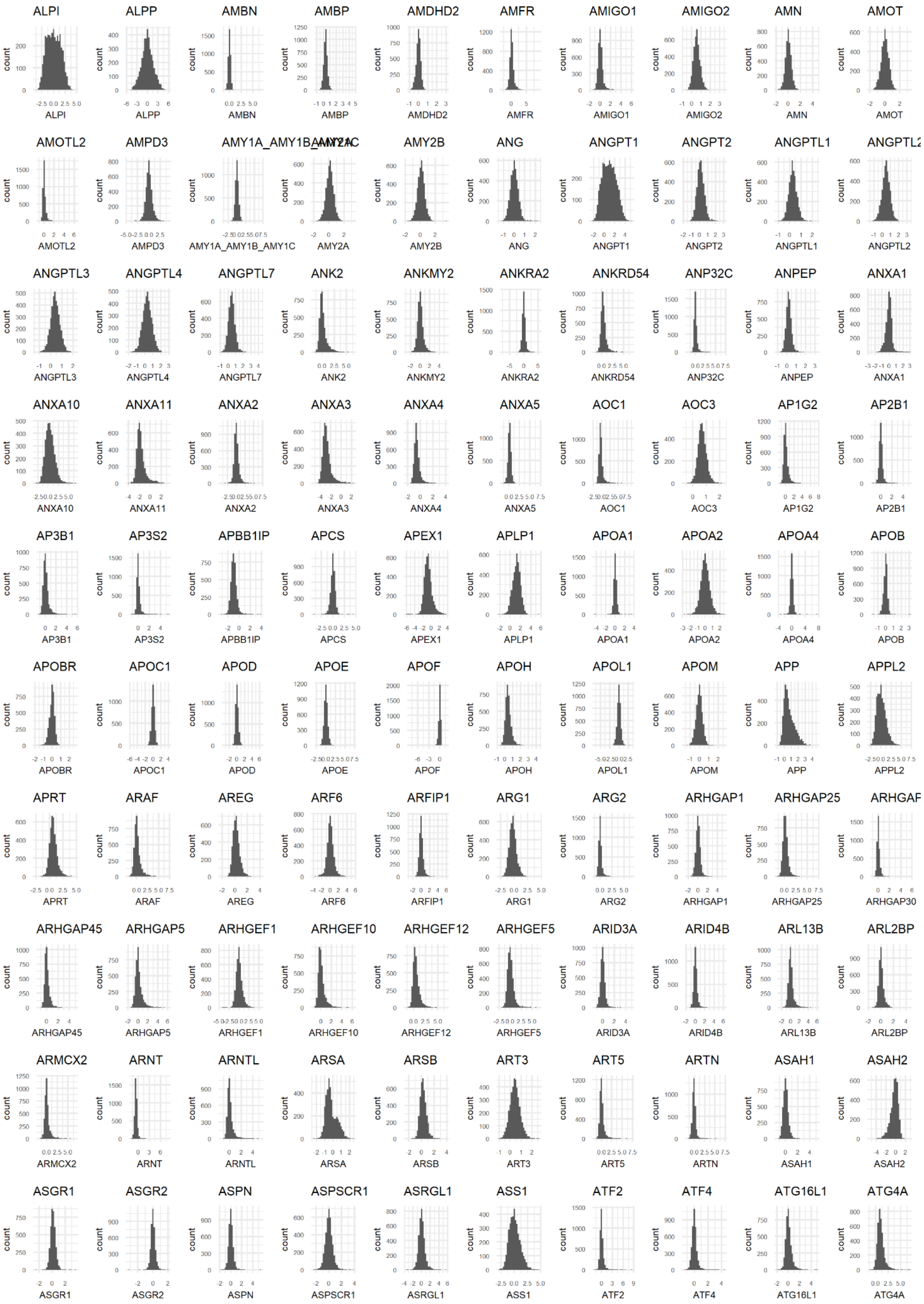
ALPI
ALPP
AMBN
AMBP
AMDHD2
AMFR
AMIGO1
AMIGO2
AMN
AMOT
AMOTL2
AMPD3
AMY1A_AMY1B_AMY1C
AMY2A
AMY2B
ANG
ANGPT1
ANGPT2
ANGPTL1
ANGPTL2
ANGPTL3
ANGPTL4
ANGPTL7
ANK2
ANKMY2
ANKRA2
ANKRD54
ANP32C
ANPEP
ANXA1
ANXA10
ANXA11
ANXA2
ANXA3
ANXA4
ANXA5
AOC1
AOC3
AP1G2
AP2B1
AP3B1
AP3S2
APBB1IP
APCS
APEX1
APLP1
APOA1
APOA2
APOA4
APOB
APOBR
APOC1
APOD
APOE
APOF
APOH
APOL1
APOM
APP
APPL2
APRT
ARAF
AREG
ARF6
ARFIP1
ARG1
ARG2
ARHGAP1
ARHGAP25
ARHGAP30
ARHGAP45
ARHGAP5
ARHGEF1
ARHGEF10
ARHGEF12
ARHGEF5
ARID3A
ARID4B
ARL13B
ARL2BP
ARMCX2
ARNT
ARNTL
ARSA
ARSB
ART3
ART5
ARTN
ASAH1
ASAH2
ASGR1
ASGR2
ASPN
ASPSCR1
ASRGL1
ASS1
ATF2
ATF4
ATG16L1
ATG4A
count

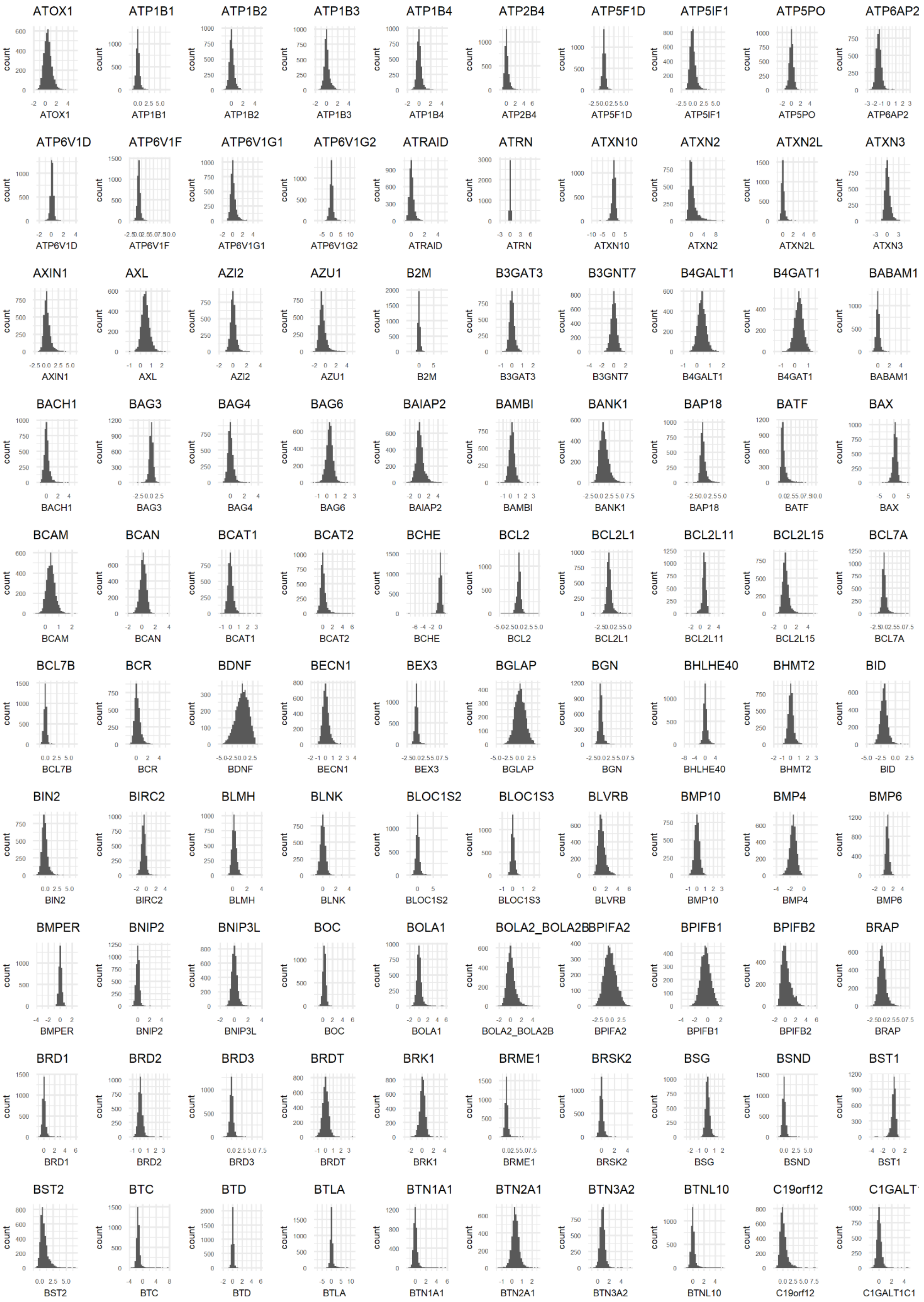
ATOX1
ATP1B1
ATP1B2
ATP1B3
ATP1B4
ATP2B4
ATP5F1D
ATP5IF1
ATP5PO
ATP6AP2
ATP6V1D
ATP6V1F
ATP6V1G1
ATP6V1G2
ATRAID
ATRN
ATXN10
ATXN2
ATXN2L
ATXN3
AXIN1
AXL
AZI2
AZU1
B2M
B3GAT3
B3GNT7
B4GALT1
B4GAT1
BABAM1
BACH1
BAG3
BAG4
BAG6
BAIAP2
BAMBI
BANK1
BAP18
BATF
BAX
BCAM
BCAN
BCAT1
BCAT2
BCHE
BCL2
BCL2L1
BCL2L11
BCL2L15
BCL7A
BCL7B
BCR
BDNF
BECN1
BEX3
BGLAP
BGN
BHLHE40
BHMT2
BID
BIN2
BIRC2
BLMH
BLNK
BLOC1S2
BLOC1S3
BLVRB
BMP10
BMP4
BMP6
BMPER
BNIP2
BNIP3L
BOC
BOLA1
BOLA2_BOLA2B
BPIFA2
BPIFB1
BPIFB2
BRAP
BRD1
BRD2
BRD3
BRDT
BRK1
BRME1
BRSK2
BSG
BSND
BST1
BST2
BTC
BTD
BTLA
BTN1A1
BTN2A1
BTN3A2
BTNL10
C19orf12
C1GALT1C1

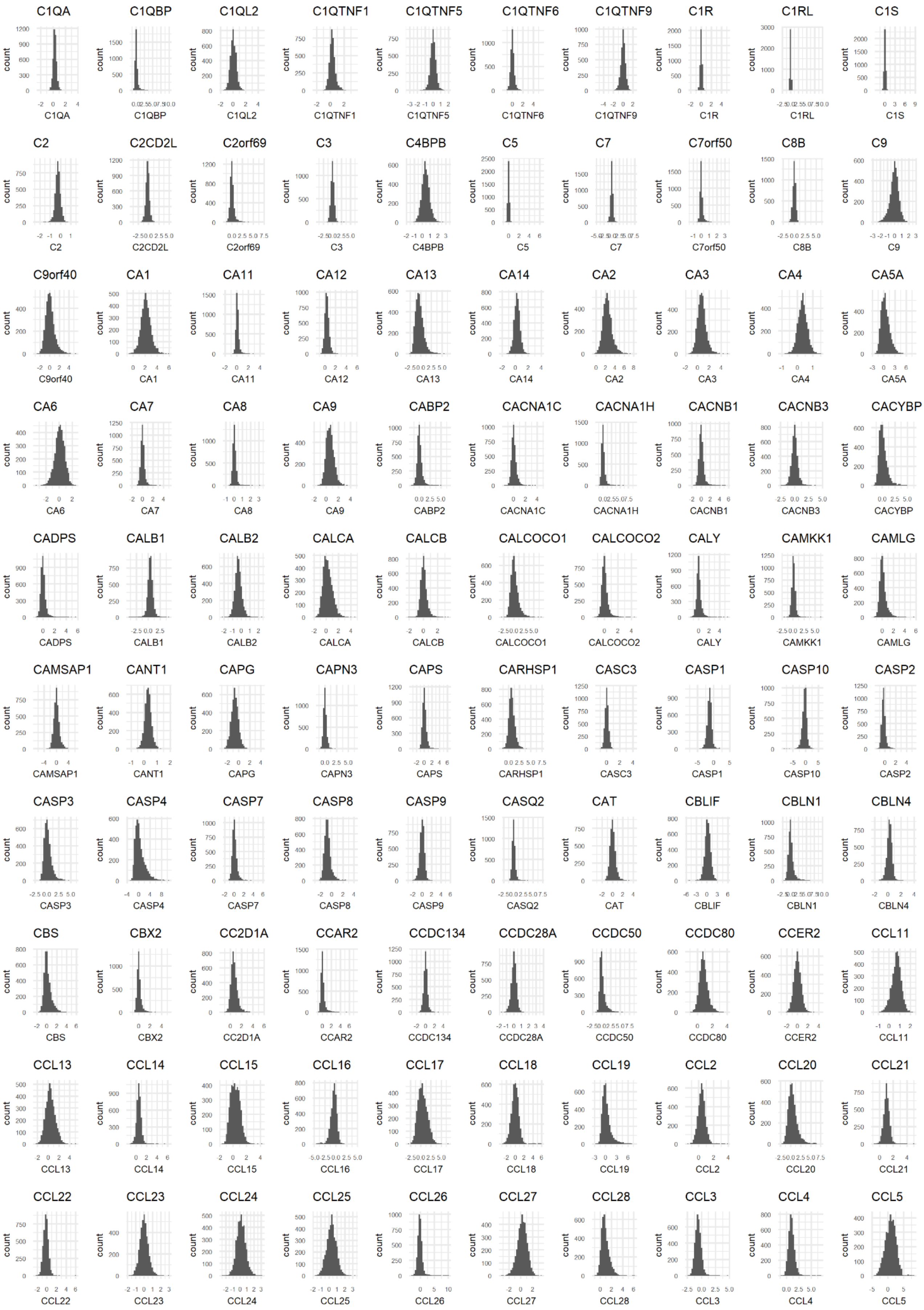
C1QA
C1QBP
C1QL2
C1QTNF1
C1QTNF5
C1QTNF6
C1QTNF9
C1R
C1RL
C1S
C2
C2CD2L
C2orf69
C3
C4BPB
C5
C7
C7orf50
C8B
C9
C9orf40
CA1
CA11
CA12
CA13
CA14
CA2
CA3
CA4
CA5A
CA6
CA7
CA8
CA9
CABP2
CACNA1C
CACNA1H
CACNB1
CACNB3
CACYBP
CADPS
CALB1
CALB2
CALCA
CALCB
CALCOCO1
CALCOCO2
CALY
CAMKK1
CAMLG
CAMSAP1
CANT1
CAPG
CAPN3
CAPS
CARHSP1
CASC3
CASP1
CASP10
CASP2
CASP3
CASP4
CASP7
CASP8
CASP9
CASQ2
CAT
CBLIF
CBLN1
CBLN4
CBS
CBX2
CC2D1A
CCAR2
CCDC134
CCDC28A
CCDC50
CCDC80
CCER2
CCL11
CCL13
CCL14
CCL15
CCL16
CCL17
CCL18
CCL19
CCL2
CCL20
CCL21
CCL22
CCL23
CCL24
CCL25
CCL26
CCL27
CCL28
CCL3
CCL4
CCL5

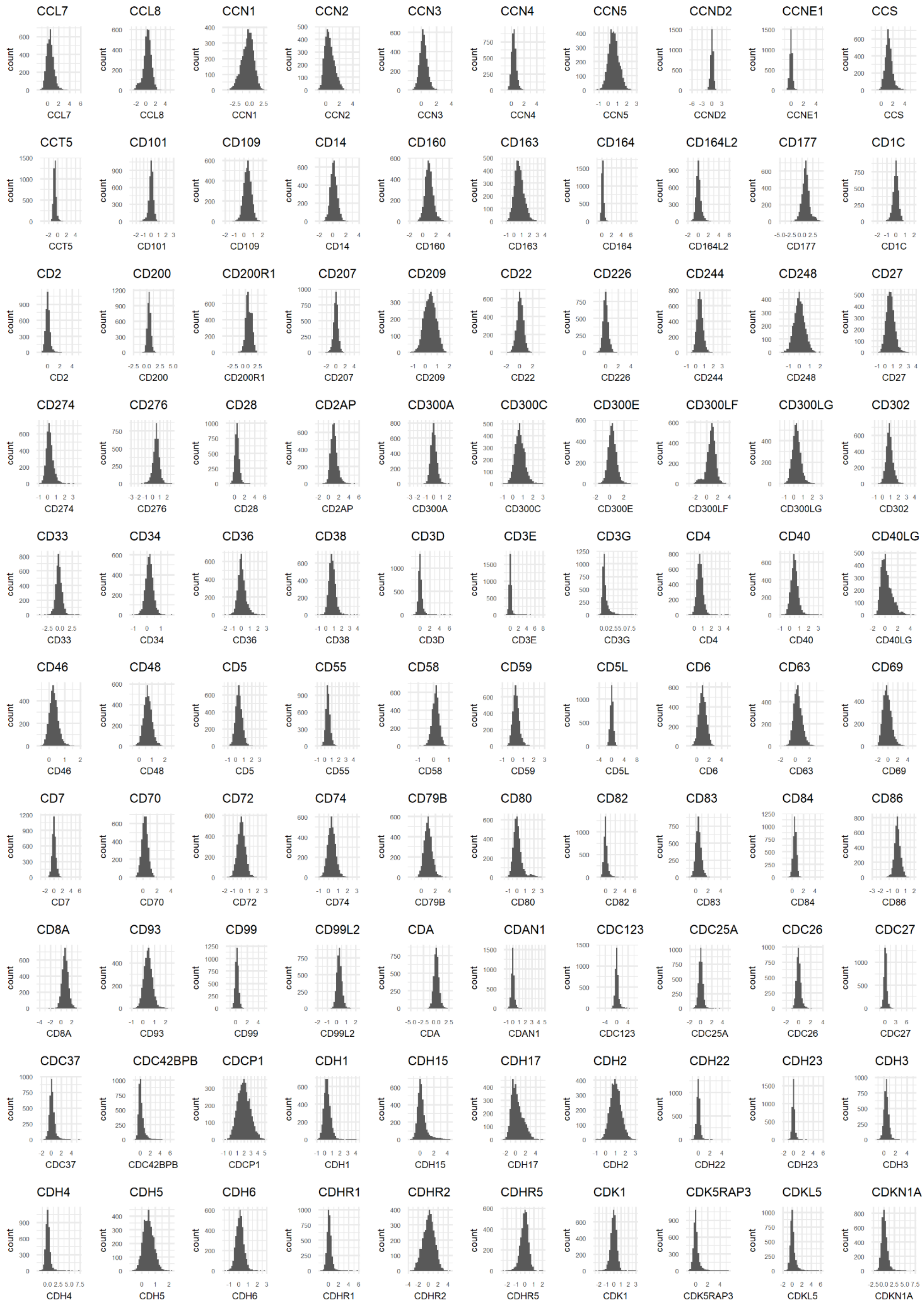
CCL7
CCL8
CCN1
CCN2
CCN3
CCN4
CCN5
CCND2
CCNE1
CCS
CCT5
CD101
CD109
CD14
CD160
CD163
CD164
CD164L2
CD177
CD1C
CD2
CD200
CD200R1
CD207
CD209
CD22
CD226
CD244
CD248
CD27
CD274
CD276
CD28
CD2AP
CD300A
CD300C
CD300E
CD300LF
CD300LG
CD302
CD33
CD34
CD36
CD38
CD3D
CD3E
CD3G
CD4
CD40
CD40LG
CD46
CD48
CD5
CD55
CD58
CD59
CD5L
CD6
CD63
CD69
CD7
CD70
CD72
CD74
CD79B
CD80
CD82
CD83
CD84
CD86
CD8A
CD93
CD99
CD99L2
CDA
CDAN1
CDC123
CDC25A
CDC26
CDC27
CDC37
CDC42BPB
CDCP1
CDH1
CDH15
CDH17
CDH2
CDH22
CDH23
CDH3
CDH4
CDH5
CDH6
CDHR1
CDHR2
CDHR5
CDK1
CDK5RAP3
CDKL5
CDKN1A

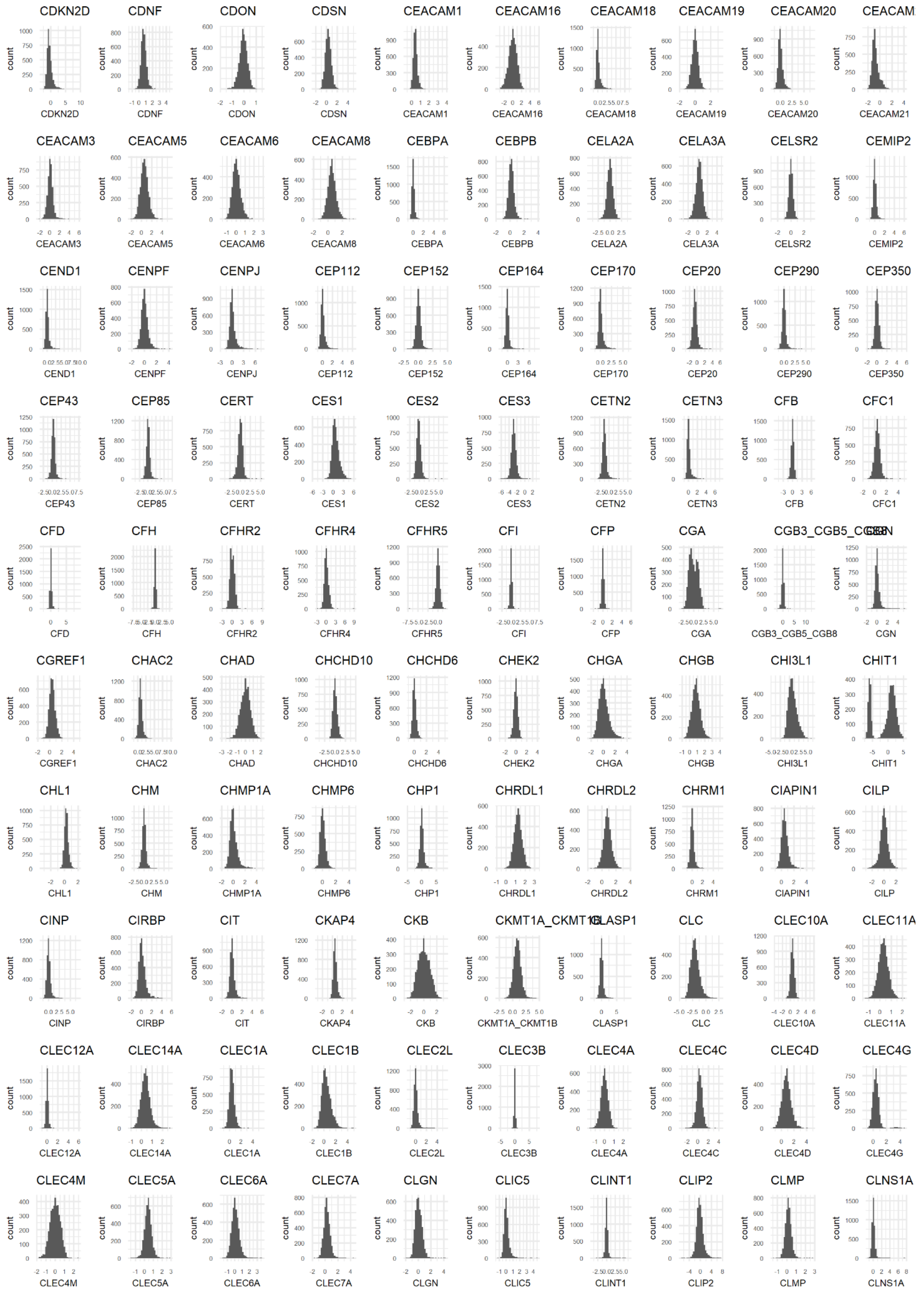
CDKN2D
CDNF
CDON
CDSN
CEACAM1
CEACAM16
CEACAM18
CEACAM19
CEACAM20
CEACAM21
CEACAM3
CEACAM5
CEACAM6
CEACAM8
CEBPA
CEBPB
CELA2A
CELA3A
CELSR2
CEMIP2
CEND1
CENPF
CENPJ
CEP112
CEP152
CEP164
CEP170
CEP20
CEP290
CEP350
CEP43
CEP85
CERT
CES1
CES2
CES3
CETN2
CETN3
CFB
CFC1
CFD
CFH
CFHR2
CFHR4
CFHR5
CFI
CFP
CGA
CGB3_CGB5_CGB8
CGN
CGREF1
CHAC2
CHAD
CHCHD10
CHCHD6
CHEK2
CHGA
CHGB
CHI3L1
CHIT1
CHL1
CHM
CHMP1A
CHMP6
CHP1
CHRDL1
CHRDL2
CHRM1
CIAPIN1
CILP
CINP
CIRBP
CIT
CKAP4
CKB
CKMT1A_CKMT1B
CLASP1
CLC
CLEC10A
CLEC11A
CLEC12A
CLEC14A
CLEC1A
CLEC1B
CLEC2L
CLEC3B
CLEC4A
CLEC4C
CLEC4D
CLEC4G
CLEC4M
CLEC5A
CLEC6A
CLEC7A
CLGN
CLIC5
CLINT1
CLIP2
CLMP
CLNS1A

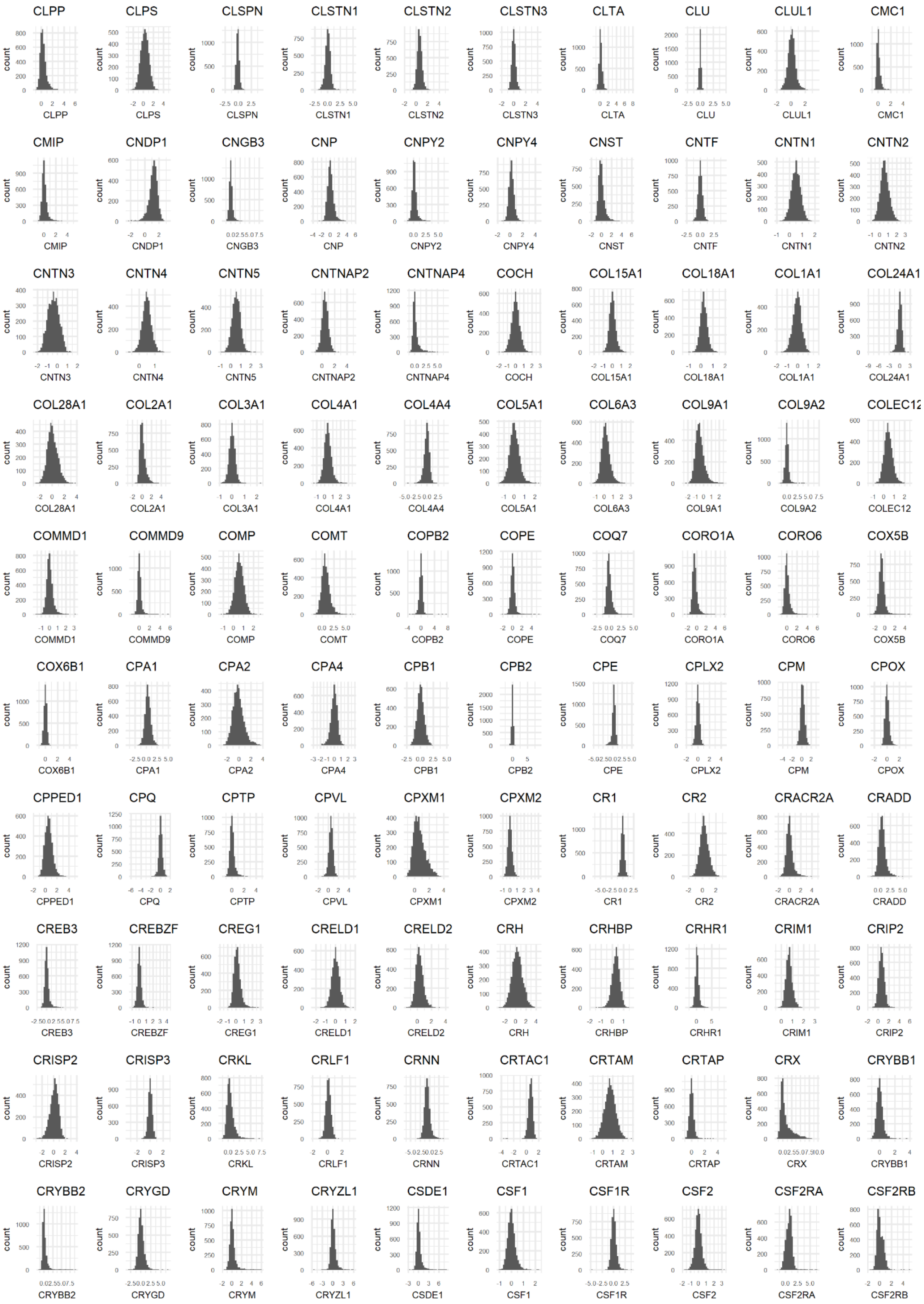
CLPP
CLPS
CLSPN
CLSTN1
CLSTN2
CLSTN3
CLTA
CLU
CLUL1
CMC1
CMIP
CNDP1
CNGB3
CNP
CNPY2
CNPY4
CNST
CNTF
CNTN1
CNTN2
CNTN3
CNTN4
CNTN5
CNTNAP2
CNTNAP4
COCH
COL15A1
COL18A1
COL1A1
COL24A1
COL28A1
COL2A1
COL3A1
COL4A1
COL4A4
COL5A1
COL6A3
COL9A1
COL9A2
COLEC12
COMMD1
COMMD9
COMP
COMT
COPB2
COPE
COQ7
CORO1A
CORO6
COX5B
COX6B1
CPA1
CPA2
CPA4
CPB1
CPB2
CPE
CPLX2
CPM
CPOX
CPPED1
CPQ
CPTP
CPVL
CPXM1
CPXM2
CR1
CR2
CRACR2A
CRADD
CREB3
CREBZF
CREG1
CRELD1
CRELD2
CRH
CRHBP
CRHR1
CRIM1
CRIP2
CRISP2
CRISP3
CRKL
CRLF1
CRNN
CRTAC1
CRTAM
CRTAP
CRX
CRYBB1
CRYBB2
CRYGD
CRYM
CRYZL1
CSDE1
CSF1
CSF1R
CSF2
CSF2RA
CSF2RB
count

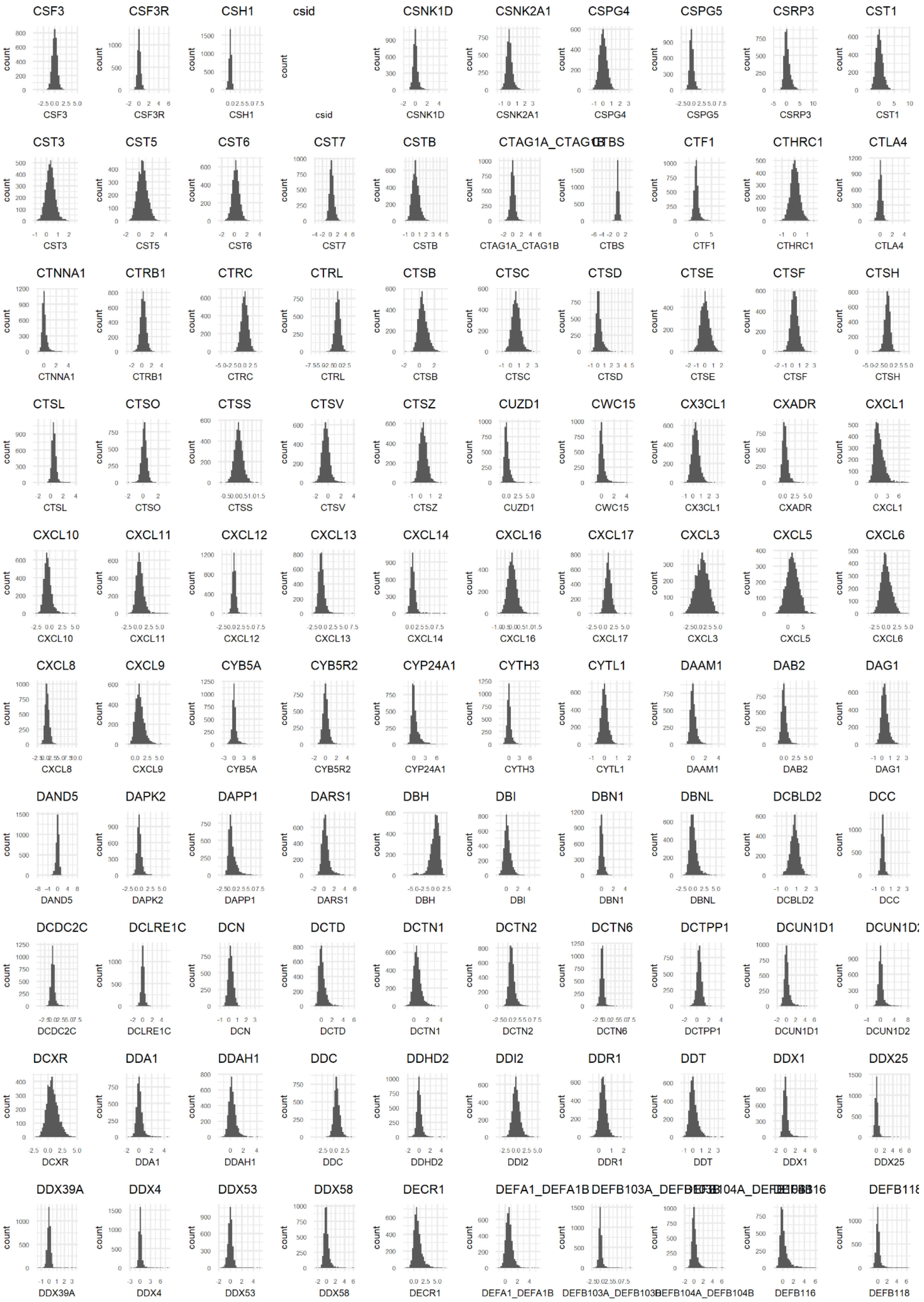
CSF3
CSF3R
CSH1
csid
CSNK1D
CSNK2A1
CSPG4
CSPG5
CSRP3
CST1
CST3
CST5
CST6
CST7
CSTB
CTAG1A_CTAG1B
CTBS
CTF1
CTHRC1
CTLA4
CTNNA1
CTRB1
CTRC
CTRL
CTSB
CTSC
CTSD
CTSE
CTSF
CTSH
CTSL
CTSO
CTSS
CTSV
CTSZ
CUZD1
CWC15
CX3CL1
CXADR
CXCL1
CXCL10
CXCL11
CXCL12
CXCL13
CXCL14
CXCL16
CXCL17
CXCL3
CXCL5
CXCL6
CXCL8
CXCL9
CYB5A
CYB5R2
CYP24A1
CYTH3
CYTL1
DAAM1
DAB2
DAG1
DAND5
DAPK2
DAPP1
DARS1
DBH
DBI
DBN1
DBNL
DCBLD2
DCC
DCDC2C
DCLRE1C
DCN
DCTD
DCTN1
DCTN2
DCTN6
DCTPP1
DCUN1D1
DCUN1D2
DCXR
DDA1
DDAH1
DDC
DDHD2
DDI2
DDR1
DDT
DDX1
DDX25
DDX39A
DDX4
DDX53
DDX58
DECR1
DEFA1_DEFA1B
DEFB103A_DEFB103B
DEFB104A_DEFB104B
DEFB116
DEFB118
count

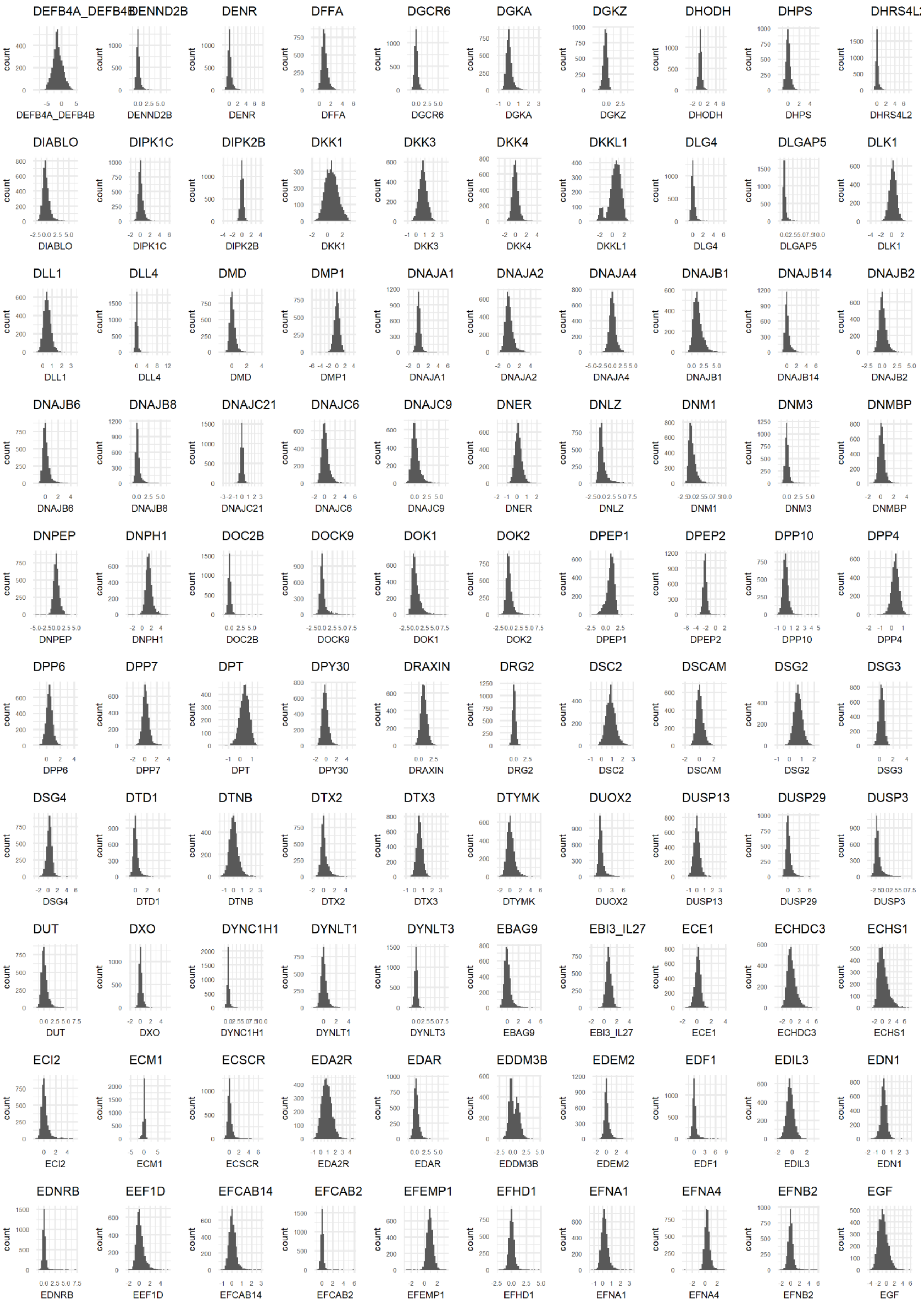
DEFB4A_DEFB4B
DENND2B
DENR
DFFA
DGCR6
DGKA
DGKZ
DHODH
DHPS
DHRS4L2
DIABLO
DIPK1C
DIPK2B
DKK1
DKK3
DKK4
DKKL1
DLG4
DLGAP5
DLK1
DLL1
DLL4
DMD
DMP1
DNAJA1
DNAJA2
DNAJA4
DNAJB1
DNAJB14
DNAJB2
DNAJB6
DNAJB8
DNAJC21
DNAJC6
DNAJC9
DNER
DNLZ
DNM1
DNM3
DNMBP
DNPEP
DNPH1
DOC2B
DOCK9
DOK1
DOK2
DPEP1
DPEP2
DPP10
DPP4
DPP6
DPP7
DPT
DPY30
DRAXIN
DRG2
DSC2
DSCAM
DSG2
DSG3
DSG4
DTD1
DTNB
DTX2
DTX3
DTYMK
DUOX2
DUSP13
DUSP29
DUSP3
DUT
DXO
DYNC1H1
DYNLT1
DYNLT3
EBAG9
EBI3_IL27
ECE1
ECHDC3
ECHS1
ECI2
ECM1
ECSCR
EDA2R
EDAR
EDDM3B
EDEM2
EDF1
EDIL3
EDN1
EDNRB
EEF1D
EFCAB14
EFCAB2
EFEMP1
EFHD1
EFNA1
EFNA4
EFNB2
EGF
count

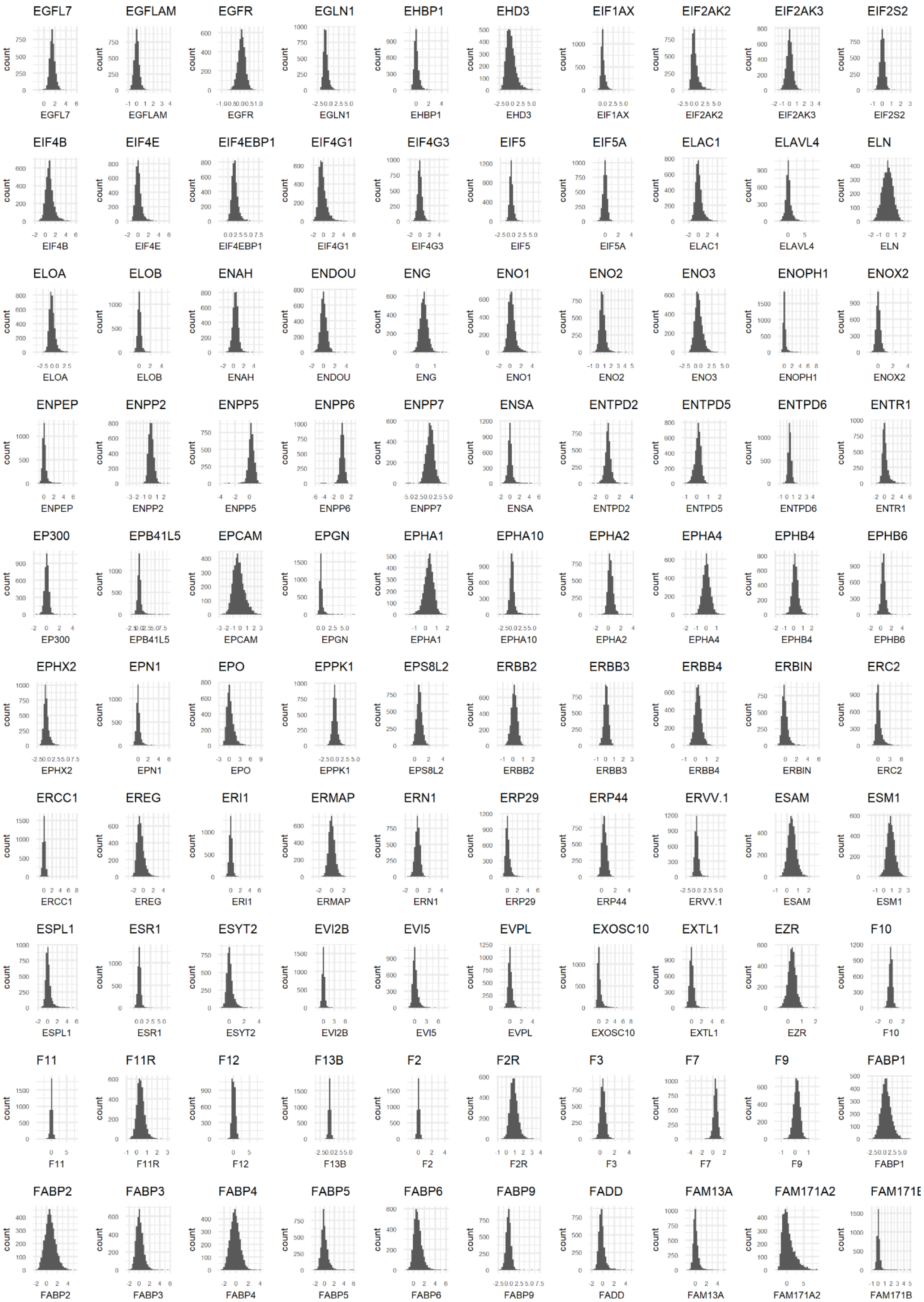
EGFL7 EGFLAM EGFR EGLN1 EHBP1 EHD3 EIF1AX EIF2AK2 EIF2AK3 EIF2S2
EIF4B EIF4E EIF4EBP1 EIF4G1 EIF4G3 EIF5 EIF5A ELAC1 ELAVL4 ELN
ELOA ELOB ENAH ENDOU ENG ENO1 ENO2 ENO3 ENOPH1 ENOX2
ENPEP ENPP2 ENPP5 ENPP6 ENPP7 ENSA ENTPD2 ENTPD5 ENTPD6 ENTR1
EP300 EPB41L5 EPCAM EPGN EPHA1 EPHA10 EPHA2 EPHA4 EPHB4 EPHB6
EPHX2 EPN1 EPO EPPK1 EPS8L2 ERBB2 ERBB3 ERBB4 ERBIN ERC2
ERCC1 EREG ERI1 ERMAP ERN1 ERP29 ERP44 ERVV.1 ESAM ESM1
ESPL1 ESR1 ESYT2 EVI2B EVI5 EVPL EXOSC10 EXTL1 EZR F10
F11 F11R F12 F13B F2 F2R F3 F7 F9 FABP1
FABP2 FABP3 FABP4 FABP5 FABP6 FABP9 FADD FAM13A FAM171A2 FAM171B
count

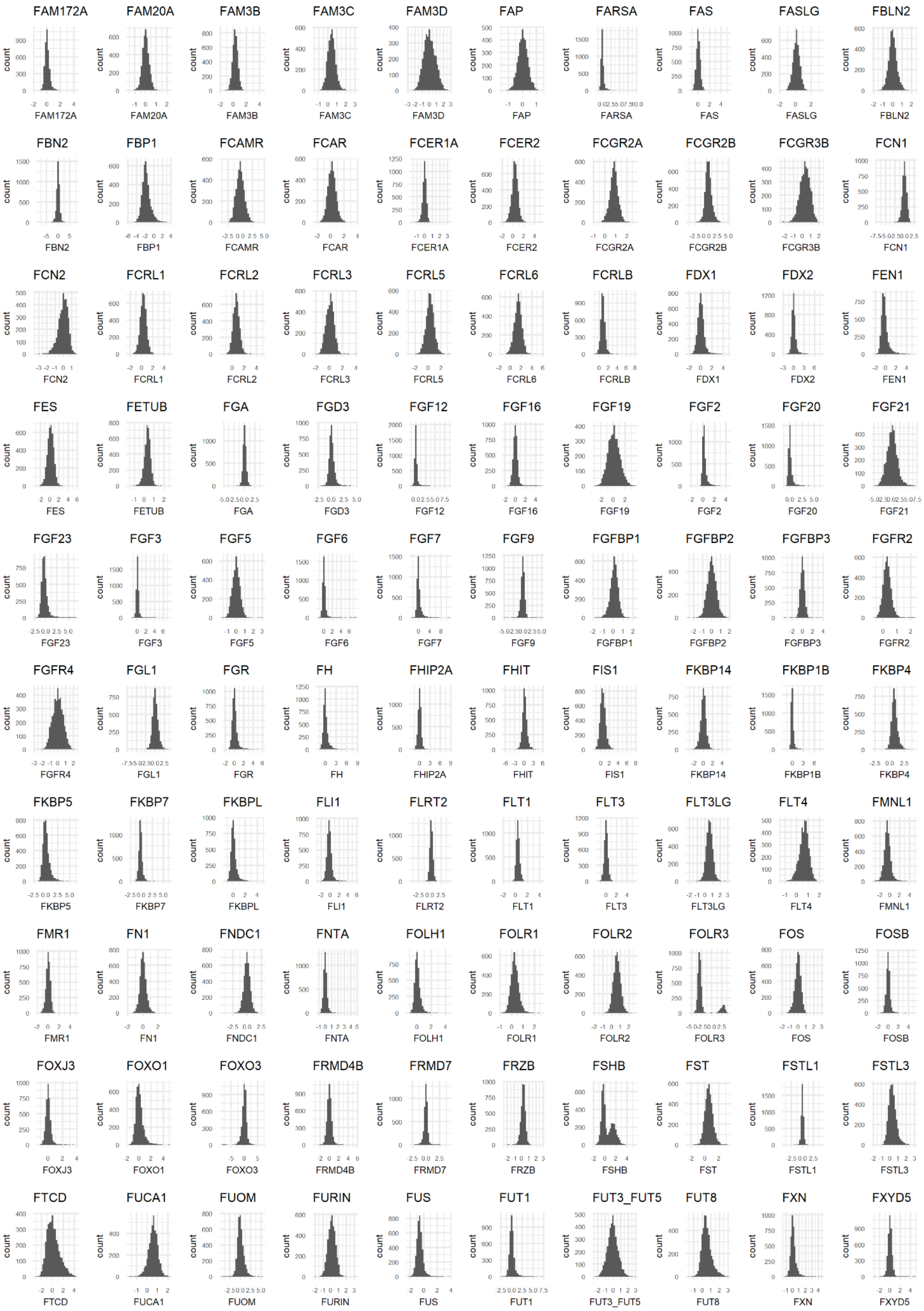
FAM172A
FAM20A
FAM3B
FAM3C
FAM3D
FAP
FARSA
FAS
FASLG
FBLN2
FBN2
FBP1
FCAMR
FCAR
FCER1A
FCER2
FCGR2A
FCGR2B
FCGR3B
FCN1
FCN2
FCRL1
FCRL2
FCRL3
FCRL5
FCRL6
FCRLB
FDX1
FDX2
FEN1
FES
FETUB
FGA
FGD3
FGF12
FGF16
FGF19
FGF2
FGF20
FGF21
FGF23
FGF3
FGF5
FGF6
FGF7
FGF9
FGFBP1
FGFBP2
FGFBP3
FGFR2
FGFR4
FGL1
FGR
FH
FHIP2A
FHIT
FIS1
FKBP14
FKBP1B
FKBP4
FKBP5
FKBP7
FKBPL
FLI1
FLRT2
FLT1
FLT3
FLT3LG
FLT4
FMNL1
FMR1
FN1
FNDC1
FNTA
FOLH1
FOLR1
FOLR2
FOLR3
FOS
FOSB
FOXJ3
FOXO1
FOXO3
FRMD4B
FRMD7
FRZB
FSHB
FST
FSTL1
FSTL3
FTCD
FUCA1
FUOM
FURIN
FUS
FUT1
FUT3_FUT5
FUT8
FXN
FXYD5
count

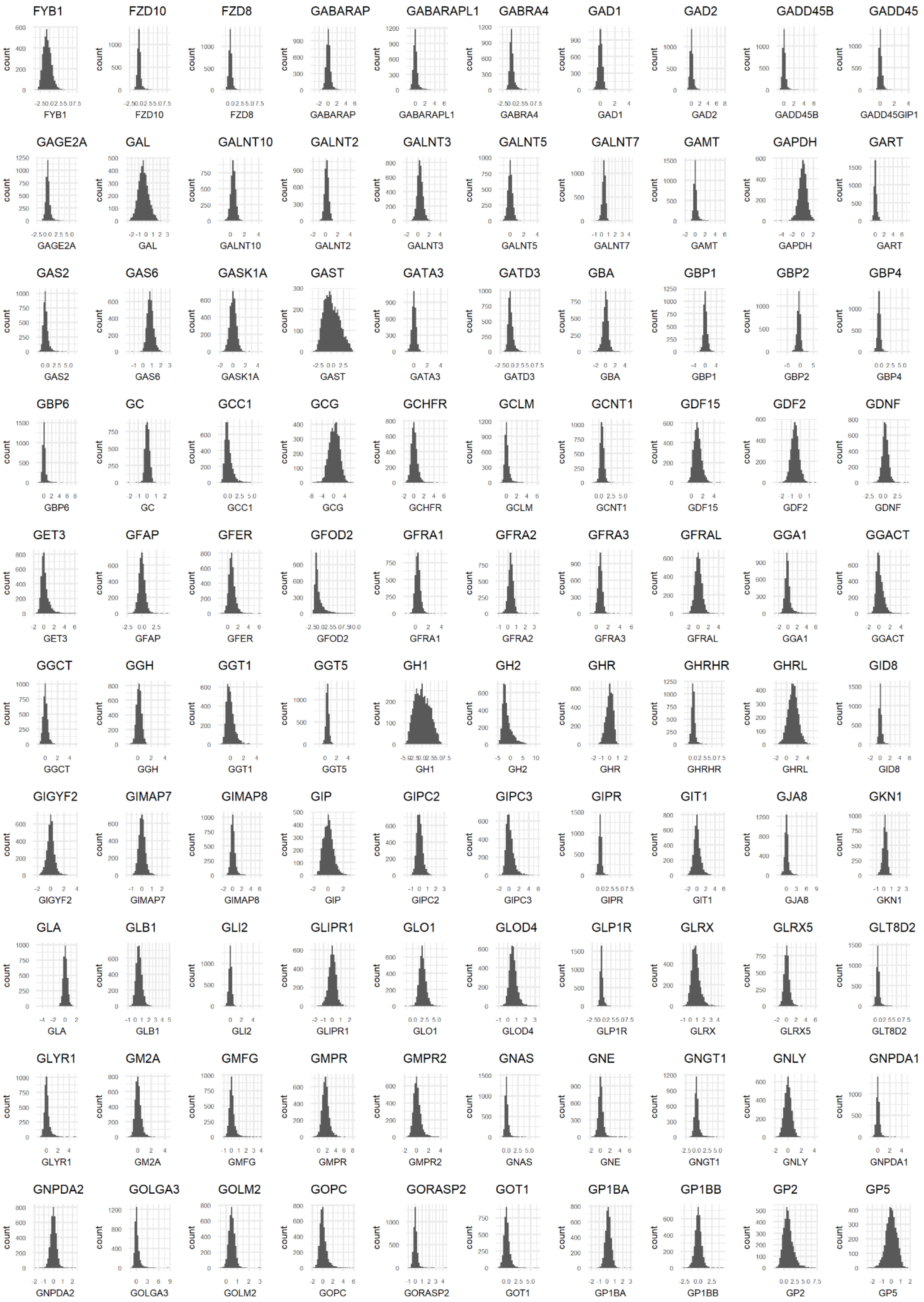
FYB1 FZD10 FZD8 GABARAP GABARAPL1 GABRA4 GAD1 GAD2 GADD45B GADD45
GAGE2A GAL GALNT10 GALNT2 GALNT3 GALNT5 GALNT7 GAMT GAPDH GART
GAS2 GAS6 GASK1A GAST GATA3 GATD3 GBA GBP1 GBP2 GBP4
GBP6 GC GCC1 GCG GCHFR GCLM GCNT1 GDF15 GDF2 GDNF
GET3 GFAP GFER GFOD2 GFRA1 GFRA2 GFRA3 GFRAL GGA1 GGACT
GGCT GGH GGT1 GGT5 GH1 GH2 GHR GHRHR GHRL GID8
GIGYF2 GIMAP7 GIMAP8 GIP GIPC2 GIPC3 GIPR GIT1 GJA8 GKN1
GLA GLB1 GLI2 GLIPR1 GLO1 GLOD4 GLP1R GLRX GLRX5 GLT8D2
GLYR1 GM2A GMFG GMPR GMPR2 GNAS GNE GNGT1 GNLY GNPDA1
GNPDA2 GOLGA3 GOLM2 GOPC GORASP2 GOT1 GP1BA GP1BB GP2 GP5
count
GADD45GIP1

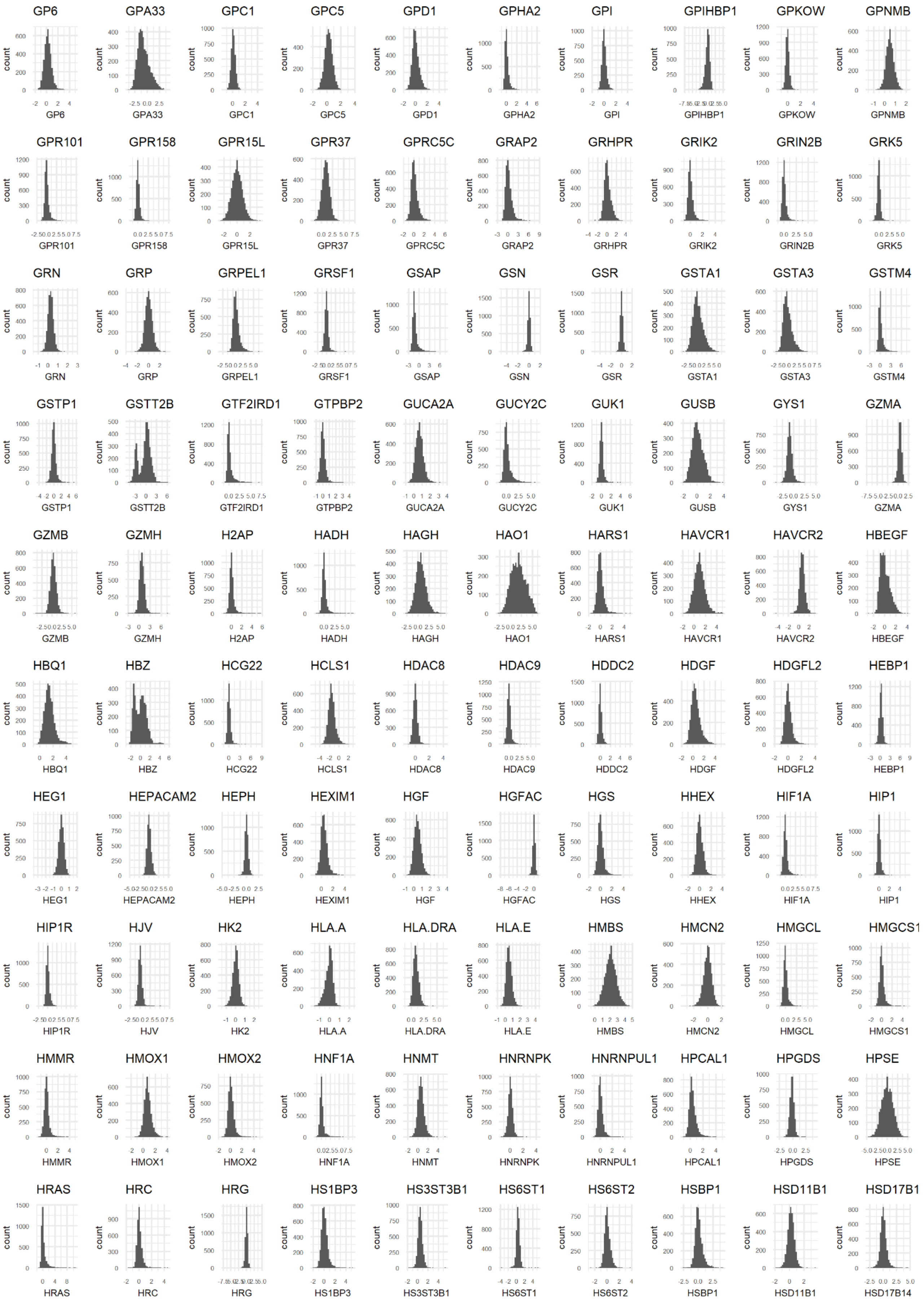
GP6
GPA33
GPC1
GPC5
GPD1
GPHA2
GPI
GPIHBP1
GPKOW
GPNMB
GPR101
GPR158
GPR15L
GPR37
GPRC5C
GRAP2
GRHPR
GRIK2
GRIN2B
GRK5
GRN
GRP
GRPEL1
GRSF1
GSAP
GSN
GSR
GSTA1
GSTA3
GSTM4
GSTP1
GSTT2B
GTF2IRD1
GTPBP2
GUCA2A
GUCY2C
GUK1
GUSB
GYS1
GZMA
GZMB
GZMH
H2AP
HADH
HAGH
HAO1
HARS1
HAVCR1
HAVCR2
HBEGF
HBQ1
HBZ
HCG22
HCLS1
HDAC8
HDAC9
HDDC2
HDGF
HDGFL2
HEBP1
HEG1
HEPACAM2
HEPH
HEXIM1
HGF
HGFAC
HGS
HHEX
HIF1A
HIP1
HIP1R
HJV
HK2
HLA.A
HLA.DRA
HLA.E
HMBS
HMCN2
HMGCL
HMGCS1
HMMR
HMOX1
HMOX2
HNF1A
HNMT
HNRNPK
HNRNPUL1
HPCAL1
HPGDS
HPSE
HRAS
HRC
HRG
HS1BP3
HS3ST3B1
HS6ST1
HS6ST2
HSBP1
HSD11B1
HSD17B1
count

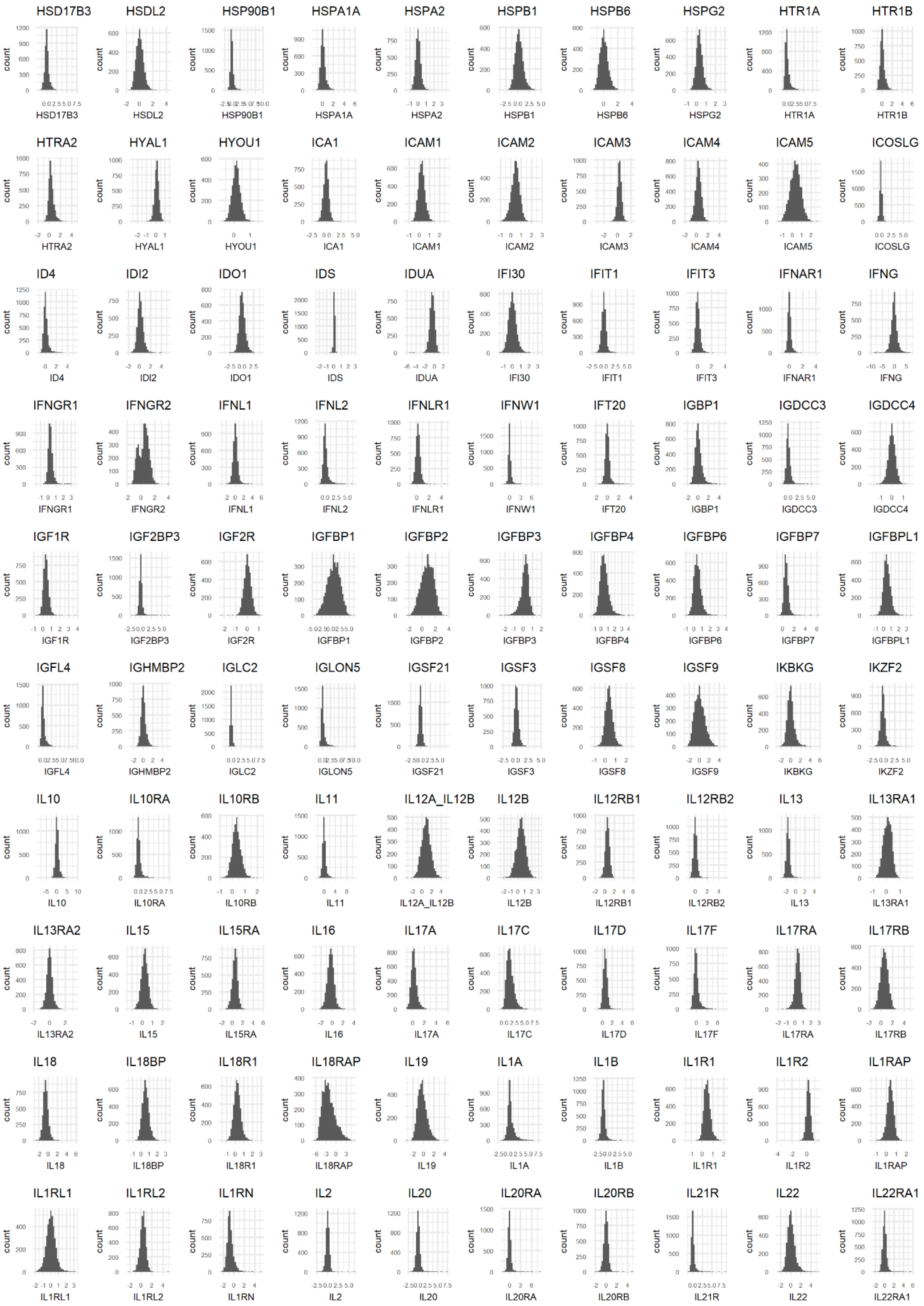
HSD17B3
HSDL2
HSP90B1
HSPA1A
HSPA2
HSPB1
HSPB6
HSPG2
HTR1A
HTR1B
HTRA2
HYAL1
HYOU1
ICA1
ICAM1
ICAM2
ICAM3
ICAM4
ICAM5
ICOSLG
ID4
IDI2
IDO1
IDS
IDUA
IFI30
IFIT1
IFIT3
IFNAR1
IFNG
IFNGR1
IFNGR2
IFNL1
IFNL2
IFNLR1
IFNW1
IFT20
IGBP1
IGDCC3
IGDCC4
IGF1R
IGF2BP3
IGF2R
IGFBP1
IGFBP2
IGFBP3
IGFBP4
IGFBP6
IGFBP7
IGFBPL1
IGFL4
IGHMBP2
IGLC2
IGLON5
IGSF21
IGSF3
IGSF8
IGSF9
IKBKG
IKZF2
IL10
IL10RA
IL10RB
IL11
IL12A_IL12B
IL12B
IL12RB1
IL12RB2
IL13
IL13RA1
IL13RA2
IL15
IL15RA
IL16
IL17A
IL17C
IL17D
IL17F
IL17RA
IL17RB
IL18
IL18BP
IL18R1
IL18RAP
IL19
IL1A
IL1B
IL1R1
IL1R2
IL1RAP
IL1RL1
IL1RL2
IL1RN
IL2
IL20
IL20RA
IL20RB
IL21R
IL22
IL22RA1
count

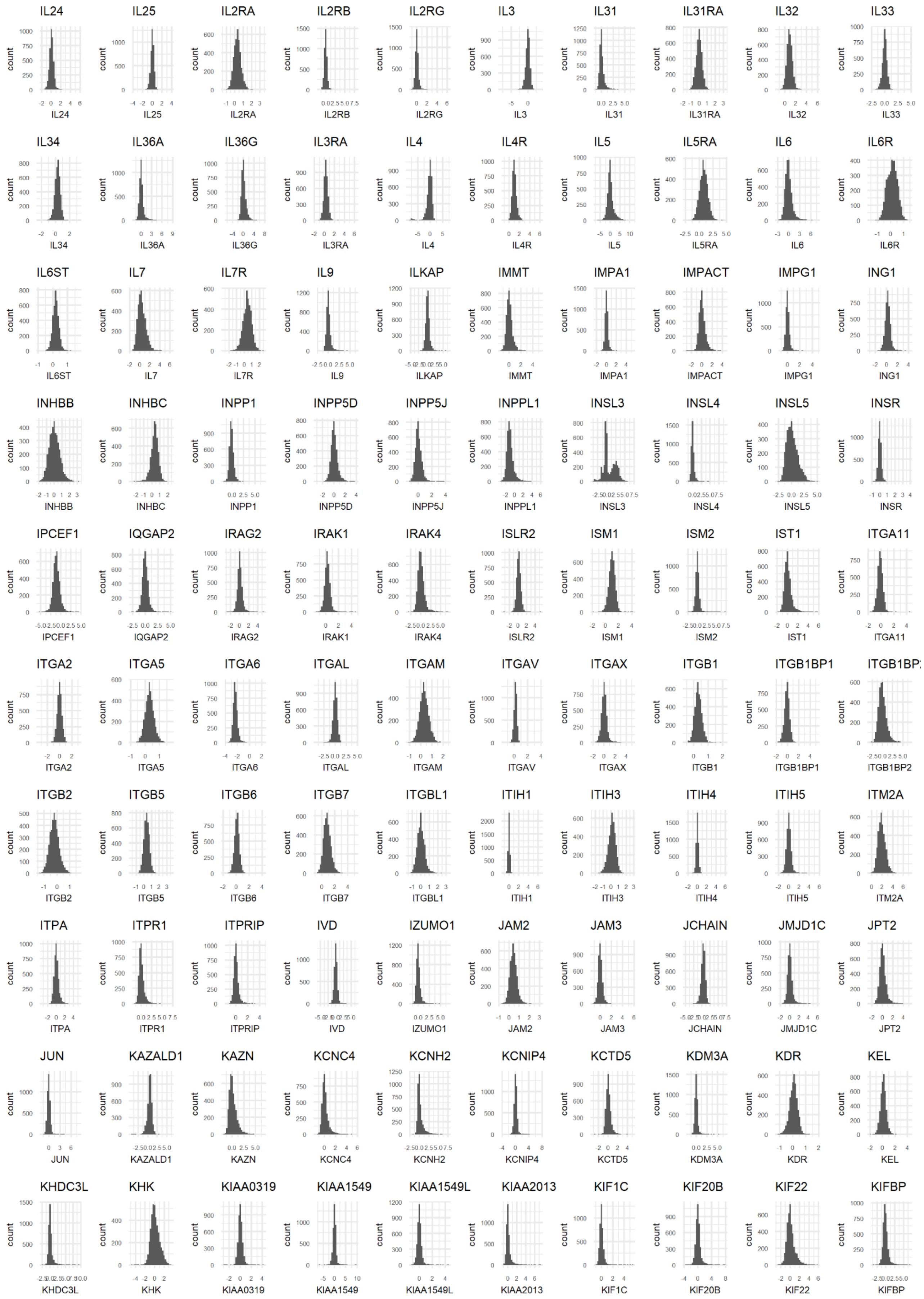
IL24
IL25
IL2RA
IL2RB
IL2RG
IL3
IL31
IL31RA
IL32
IL33
IL34
IL36A
IL36G
IL3RA
IL4
IL4R
IL5
IL5RA
IL6
IL6R
IL6ST
IL7
IL7R
IL9
ILKAP
IMMT
IMPA1
IMPACT
IMPG1
ING1
INHBB
INHBC
INPP1
INPP5D
INPP5J
INPPL1
INSL3
INSL4
INSL5
INSR
IPCEF1
IQGAP2
IRAG2
IRAK1
IRAK4
ISLR2
ISM1
ISM2
IST1
ITGA11
ITGA2
ITGA5
ITGA6
ITGAL
ITGAM
ITGAV
ITGAX
ITGB1
ITGB1BP1
ITGB1BP2
ITGB2
ITGB5
ITGB6
ITGB7
ITGBL1
ITIH1
ITIH3
ITIH4
ITIH5
ITM2A
ITPA
ITPR1
ITPRIP
IVD
IZUMO1
JAM2
JAM3
JCHAIN
JMJD1C
JPT2
JUN
KAZALD1
KAZN
KCNC4
KCNH2
KCNIP4
KCTD5
KDM3A
KDR
KEL
KHDC3L
KHK
KIAA0319
KIAA1549
KIAA1549L
KIAA2013
KIF1C
KIF20B
KIF22
KIFBP
count

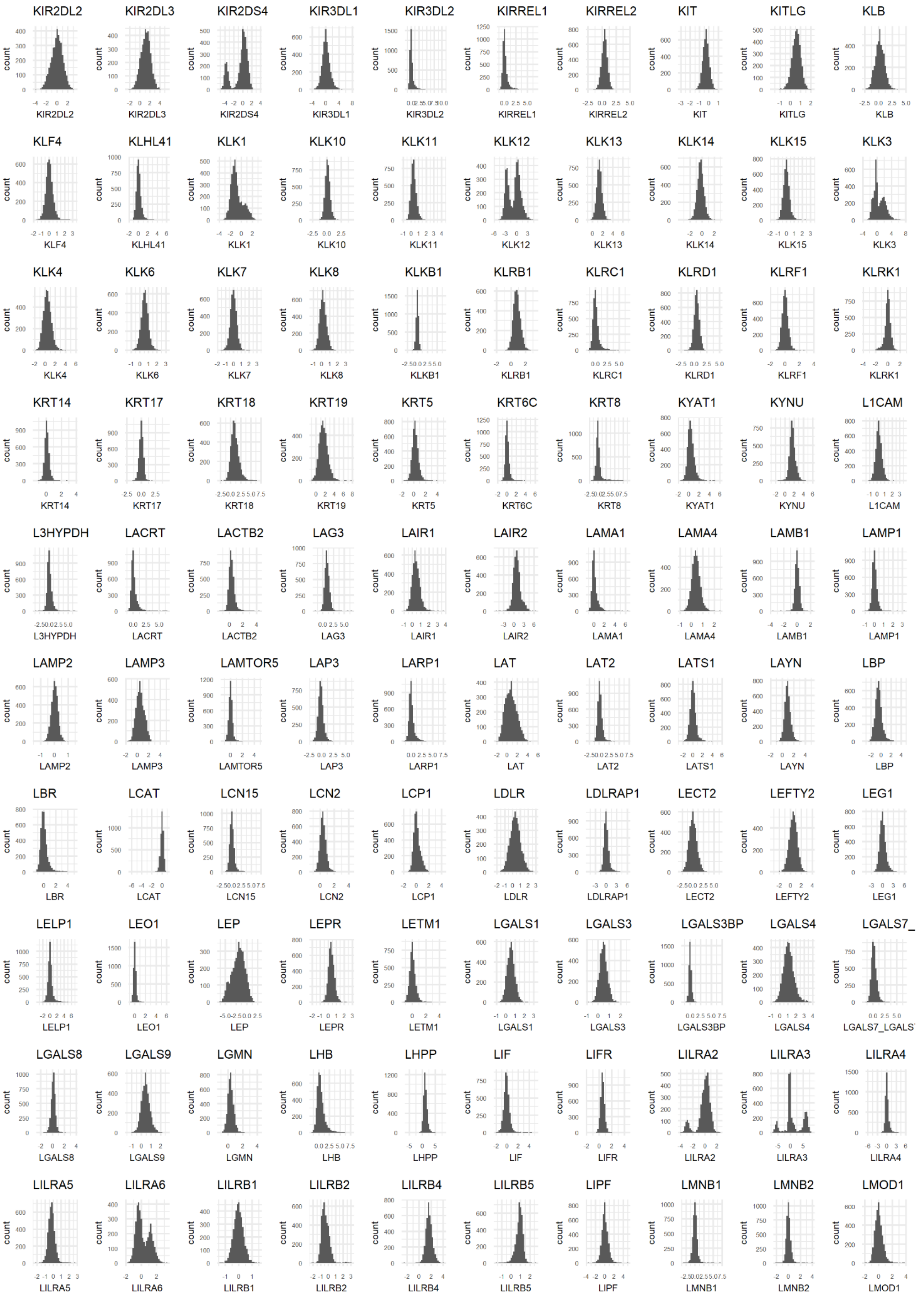
KIR2DL2
KIR2DL3
KIR2DS4
KIR3DL1
KIR3DL2
KIRREL1
KIRREL2
KIT
KITLG
KLB
KLF4
KLHL41
KLK1
KLK10
KLK11
KLK12
KLK13
KLK14
KLK15
KLK3
KLK4
KLK6
KLK7
KLK8
KLKB1
KLRB1
KLRC1
KLRD1
KLRF1
KLRK1
KRT14
KRT17
KRT18
KRT19
KRT5
KRT6C
KRT8
KYAT1
KYNU
L1CAM
L3HYPDH
LACRT
LACTB2
LAG3
LAIR1
LAIR2
LAMA1
LAMA4
LAMB1
LAMP1
LAMP2
LAMP3
LAMTOR5
LAP3
LARP1
LAT
LAT2
LATS1
LAYN
LBP
LBR
LCAT
LCN15
LCN2
LCP1
LDLR
LDLRAP1
LECT2
LEFTY2
LEG1
LELP1
LEO1
LEP
LEPR
LETM1
LGALS1
LGALS3
LGALS3BP
LGALS4
LGALS7_
LGALS8
LGALS9
LGMN
LHB
LHPP
LIF
LIFR
LILRA2
LILRA3
LILRA4
LILRA5
LILRA6
LILRB1
LILRB2
LILRB4
LILRB5
LIPF
LMNB1
LMNB2
LMOD1
count

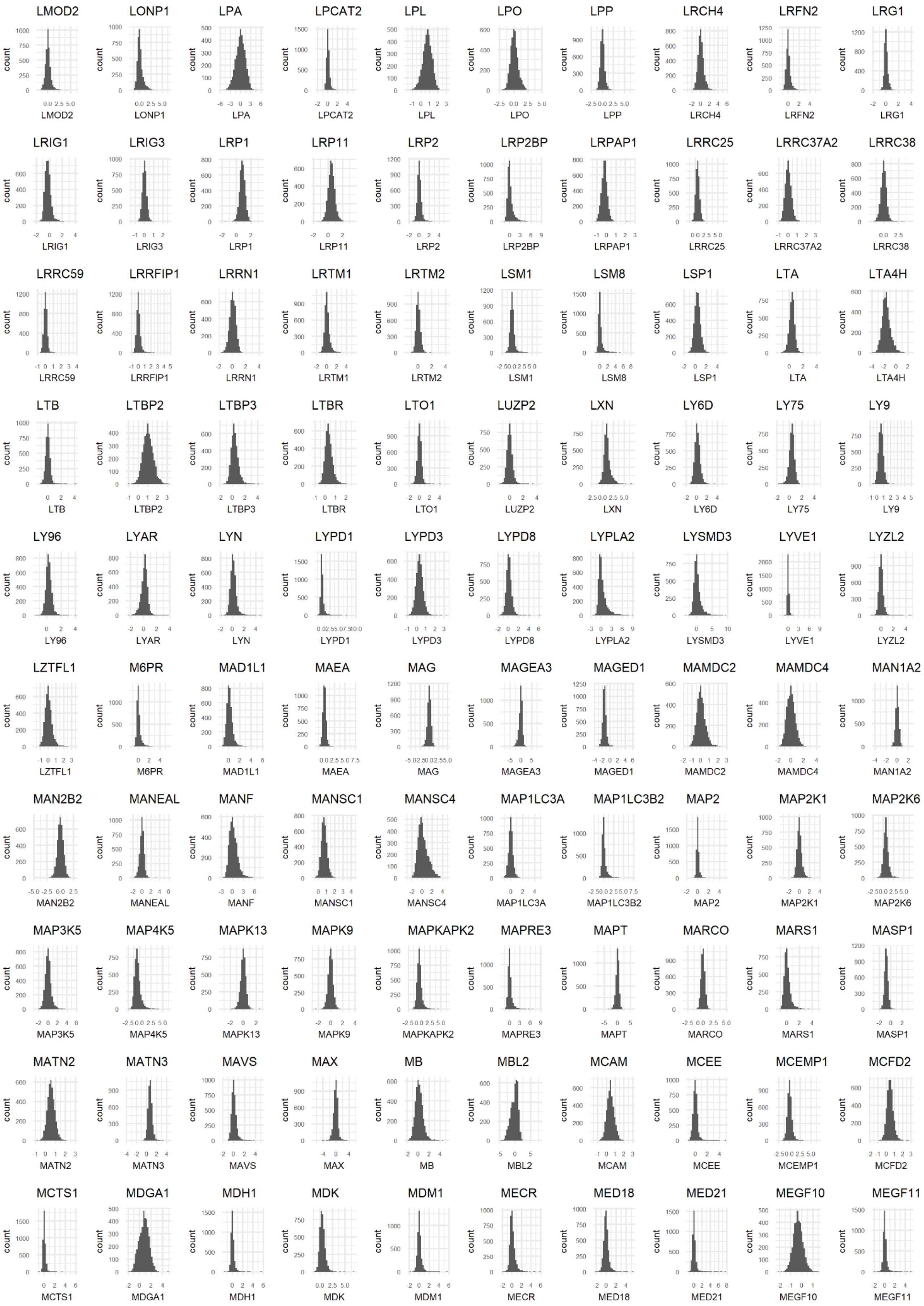
LMOD2
LONP1
LPA
LPCAT2
LPL
LPO
LPP
LRCH4
LRFN2
LRG1
LRIG1
LRIG3
LRP1
LRP11
LRP2
LRP2BP
LRPAP1
LRRC25
LRRC37A2
LRRC38
LRRC59
LRRFIP1
LRRN1
LRTM1
LRTM2
LSM1
LSM8
LSP1
LTA
LTA4H
LTB
LTBP2
LTBP3
LTBR
LTO1
LUZP2
LXN
LY6D
LY75
LY9
LY96
LYAR
LYN
LYPD1
LYPD3
LYPD8
LYPLA2
LYSMD3
LYVE1
LYZL2
LZTFL1
M6PR
MAD1L1
MAEA
MAG
MAGEA3
MAGED1
MAMDC2
MAMDC4
MAN1A2
MAN2B2
MANEAL
MANF
MANSC1
MANSC4
MAP1LC3A
MAP1LC3B2
MAP2
MAP2K1
MAP2K6
MAP3K5
MAP4K5
MAPK13
MAPK9
MAPKAPK2
MAPRE3
MAPT
MARCO
MARS1
MASP1
MATN2
MATN3
MAVS
MAX
MB
MBL2
MCAM
MCEE
MCEMP1
MCFD2
MCTS1
MDGA1
MDH1
MDK
MDM1
MECR
MED18
MED21
MEGF10
MEGF11
count

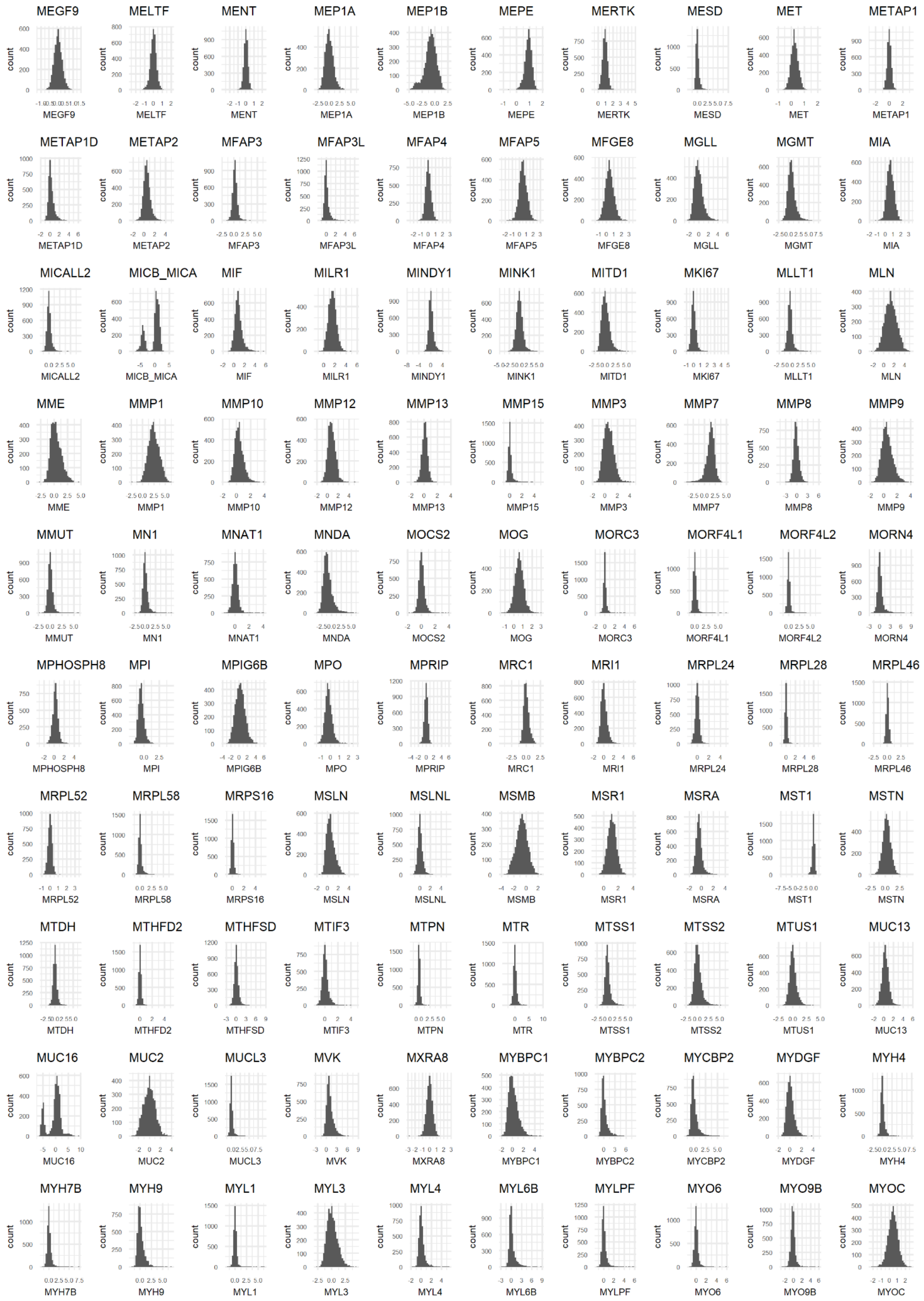
MEGF9 MELTF MENT MEP1A MEP1B MEPE MERTK MESD MET METAP1
METAP1D METAP2 MFAP3 MFAP3L MFAP4 MFAP5 MFGE8 MGLL MGMT MIA
MICALL2 MICB_MICA MIF MILR1 MINDY1 MINK1 MITD1 MKI67 MLLT1 MLN
MME MMP1 MMP10 MMP12 MMP13 MMP15 MMP3 MMP7 MMP8 MMP9
MMUT MN1 MNAT1 MNDA MOCS2 MOG MORC3 MORF4L1 MORF4L2 MORN4
MPHOSPH8 MPI MPIG6B MPO MPRIP MRC1 MRI1 MRPL24 MRPL28 MRPL46
MRPL52 MRPL58 MRPS16 MSLN MSLNL MSMB MSR1 MSRA MST1 MSTN
MTDH MTHFD2 MTHFSD MTIF3 MTPN MTR MTSS1 MTSS2 MTUS1 MUC13
MUC16 MUC2 MUCL3 MVK MXRA8 MYBPC1 MYBPC2 MYCBP2 MYDGF MYH4
MYH7B MYH9 MYL1 MYL3 MYL4 MYL6B MYLPF MYO6 MYO9B MYOC
count

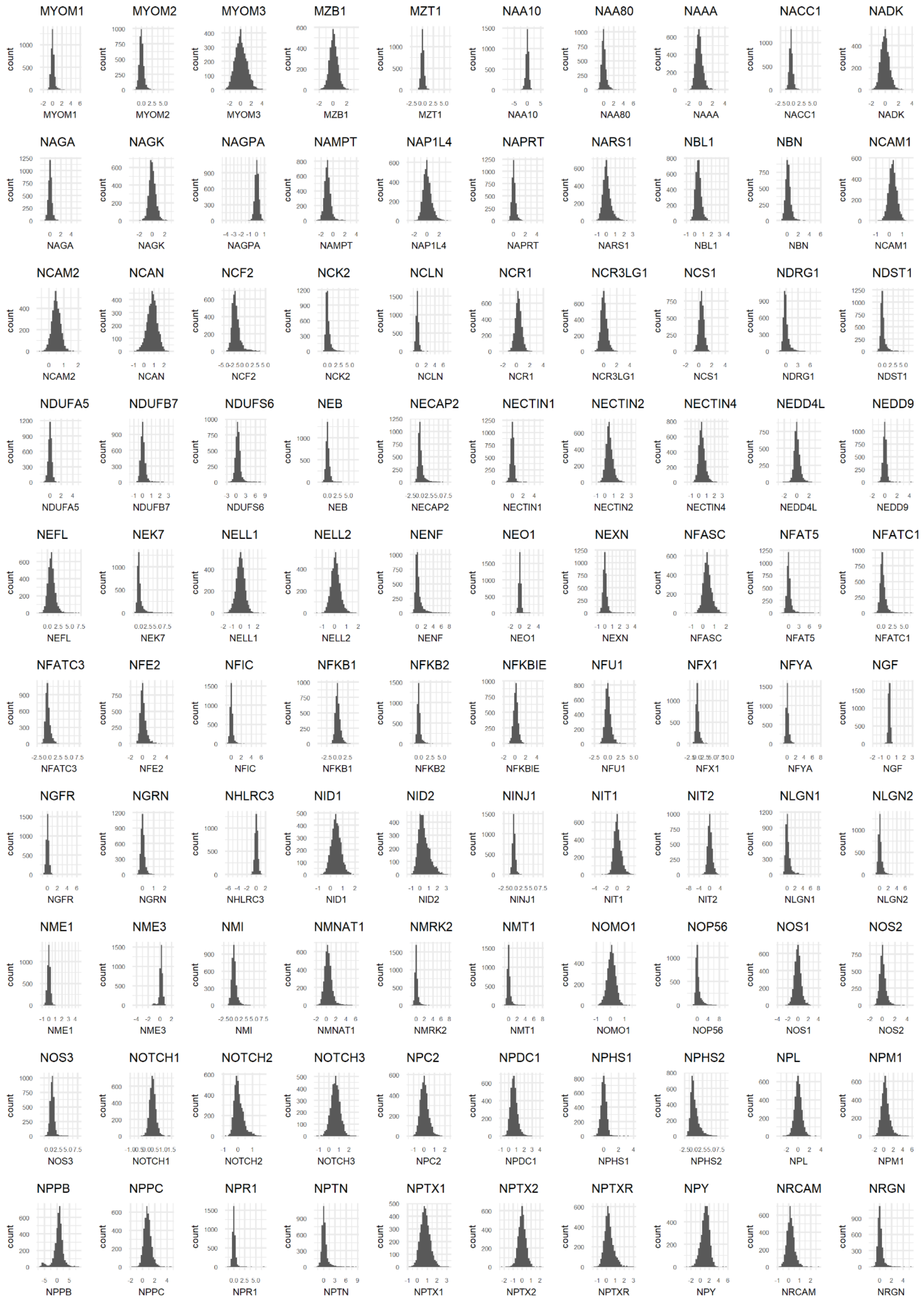
MYOM1
MYOM2
MYOM3
MZB1
MZT1
NAA10
NAA80
NAAA
NACC1
NADK
NAGA
NAGK
NAGPA
NAMPT
NAP1L4
NAPRT
NARS1
NBL1
NBN
NCAM1
NCAM2
NCAN
NCF2
NCK2
NCLN
NCR1
NCR3LG1
NCS1
NDRG1
NDST1
NDUFA5
NDUFB7
NDUFS6
NEB
NECAP2
NECTIN1
NECTIN2
NECTIN4
NEDD4L
NEDD9
NEFL
NEK7
NELL1
NELL2
NENF
NEO1
NEXN
NFASC
NFAT5
NFATC1
NFATC3
NFE2
NFIC
NFKB1
NFKB2
NFKBIE
NFU1
NFX1
NFYA
NGF
NGFR
NGRN
NHLRC3
NID1
NID2
NINJ1
NIT1
NIT2
NLGN1
NLGN2
NME1
NME3
NMI
NMNAT1
NMRK2
NMT1
NOMO1
NOP56
NOS1
NOS2
NOS3
NOTCH1
NOTCH2
NOTCH3
NPC2
NPDC1
NPHS1
NPHS2
NPL
NPM1
NPPB
NPPC
NPR1
NPTN
NPTX1
NPTX2
NPTXR
NPY
NRCAM
NRGN

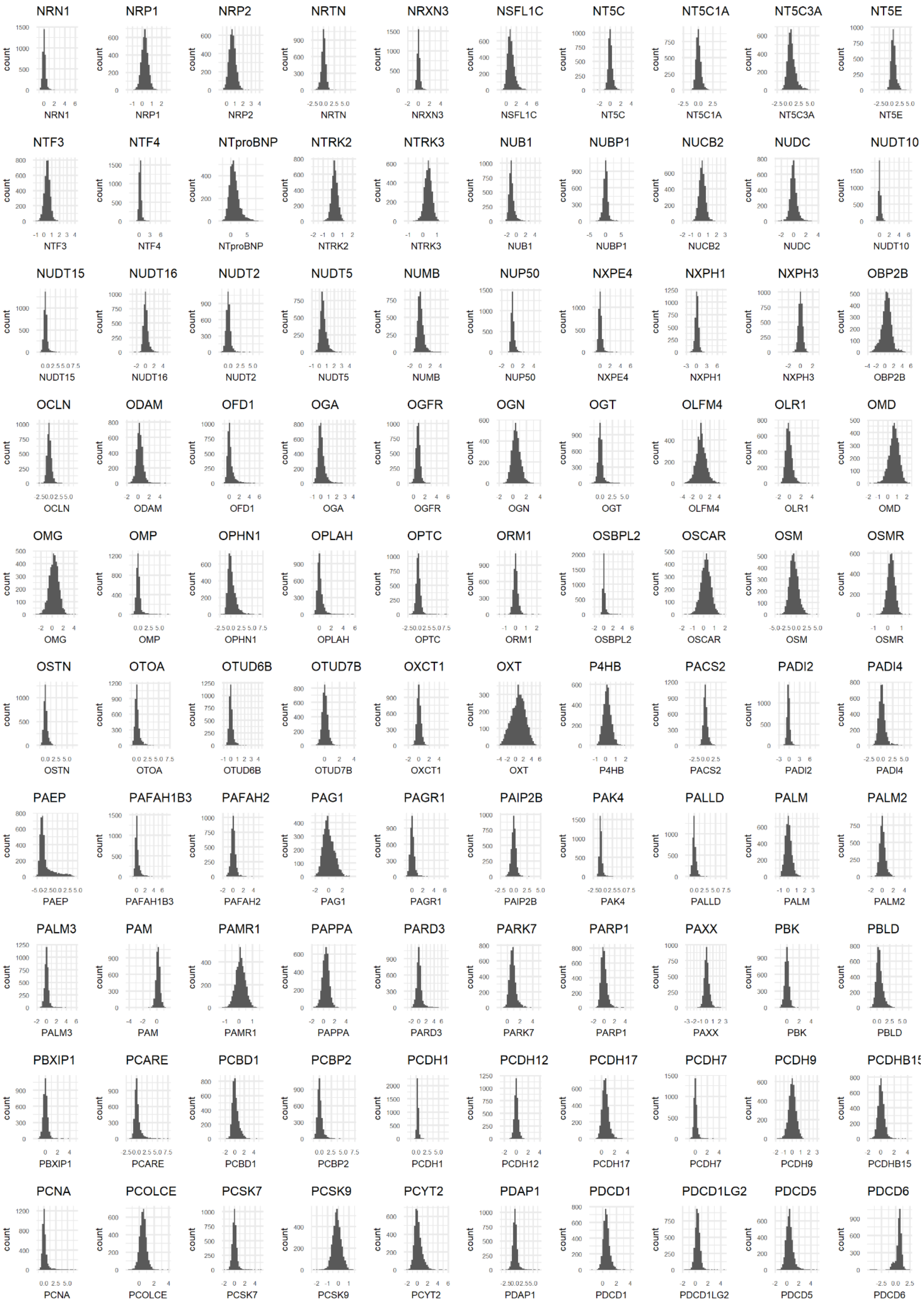
NRN1
NRP1
NRP2
NRTN
NRXN3
NSFL1C
NT5C
NT5C1A
NT5C3A
NT5E
NTF3
NTF4
NTproBNP
NTRK2
NTRK3
NUB1
NUBP1
NUCB2
NUDC
NUDT10
NUDT15
NUDT16
NUDT2
NUDT5
NUMB
NUP50
NXPE4
NXPH1
NXPH3
OBP2B
OCLN
ODAM
OFD1
OGA
OGFR
OGN
OGT
OLFM4
OLR1
OMD
OMG
OMP
OPHN1
OPLAH
OPTC
ORM1
OSBPL2
OSCAR
OSM
OSMR
OSTN
OTOA
OTUD6B
OTUD7B
OXCT1
OXT
P4HB
PACS2
PADI2
PADI4
PAEP
PAFAH1B3
PAFAH2
PAG1
PAGR1
PAIP2B
PAK4
PALLD
PALM
PALM2
PALM3
PAM
PAMR1
PAPPA
PARD3
PARK7
PARP1
PAXX
PBK
PBLD
PBXIP1
PCARE
PCBD1
PCBP2
PCDH1
PCDH12
PCDH17
PCDH7
PCDH9
PCDHB15
PCNA
PCOLCE
PCSK7
PCSK9
PCYT2
PDAP1
PDCD1
PDCD1LG2
PDCD5
PDCD6

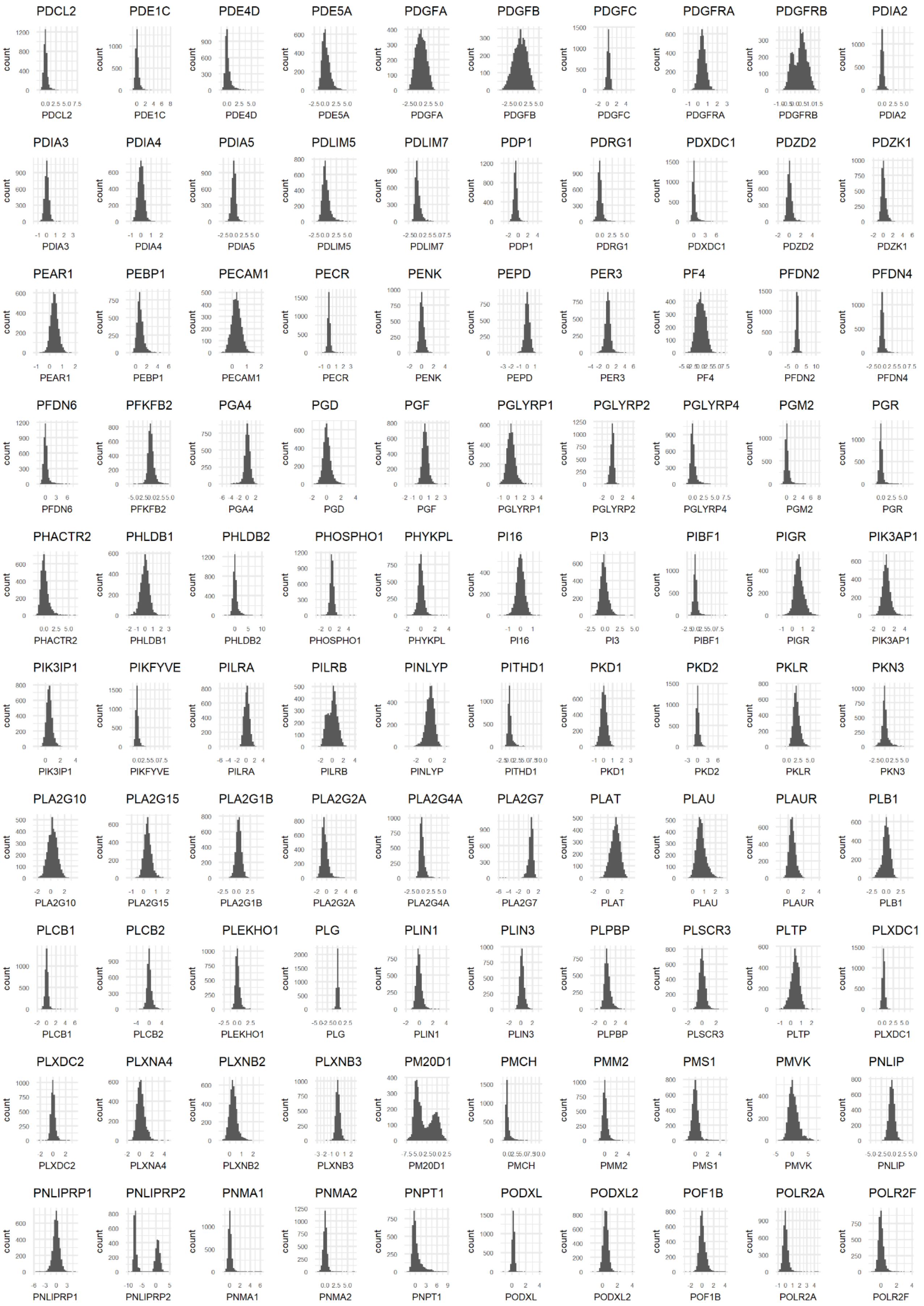
PDCL2
PDE1C
PDE4D
PDE5A
PDGFA
PDGFB
PDGFC
PDGFRA
PDGFRB
PDIA2
PDIA3
PDIA4
PDIA5
PDLIM5
PDLIM7
PDP1
PDRG1
PDXDC1
PDZD2
PDZK1
PEAR1
PEBP1
PECAM1
PECR
PENK
PEPD
PER3
PF4
PFDN2
PFDN4
PFDN6
PFKFB2
PGA4
PGD
PGF
PGLYRP1
PGLYRP2
PGLYRP4
PGM2
PGR
PHACTR2
PHLDB1
PHLDB2
PHOSPHO1
PHYKPL
PI16
PI3
PIBF1
PIGR
PIK3AP1
PIK3IP1
PIKFYVE
PILRA
PILRB
PINLYP
PITHD1
PKD1
PKD2
PKLR
PKN3
PLA2G10
PLA2G15
PLA2G1B
PLA2G2A
PLA2G4A
PLA2G7
PLAT
PLAU
PLAUR
PLB1
PLCB1
PLCB2
PLEKHO1
PLG
PLIN1
PLIN3
PLPBP
PLSCR3
PLTP
PLXDC1
PLXDC2
PLXNA4
PLXNB2
PLXNB3
PM20D1
PMCH
PMM2
PMS1
PMVK
PNLIP
PNLIPRP1
PNLIPRP2
PNMA1
PNMA2
PNPT1
PODXL
PODXL2
POF1B
POLR2A
POLR2F

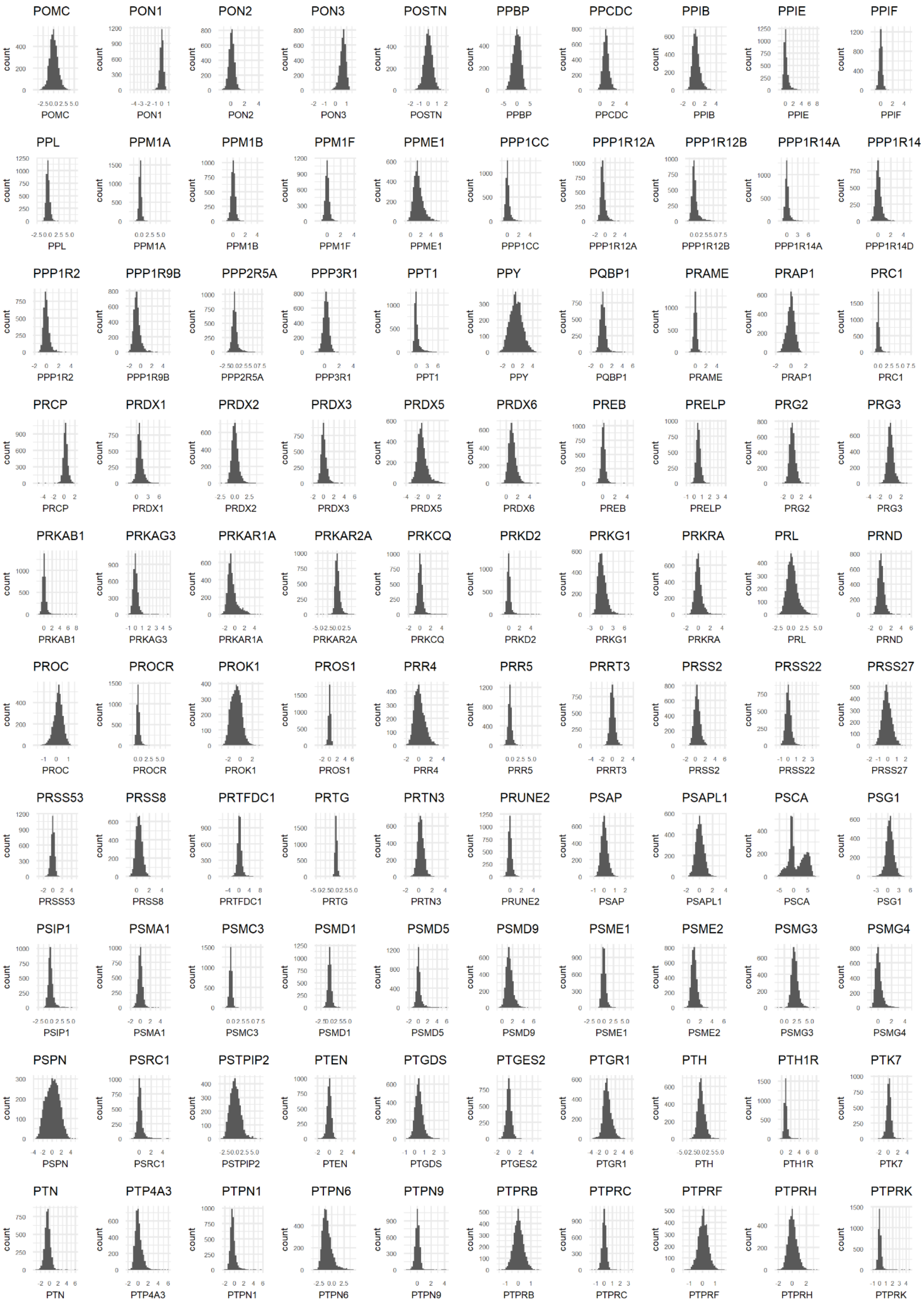
POMC
PON1
PON2
PON3
POSTN
PPBP
PPCDC
PPIB
PPIE
PPIF
PPL
PPM1A
PPM1B
PPM1F
PPME1
PPP1CC
PPP1R12A
PPP1R12B
PPP1R14A
PPP1R14
PPP1R14D
PPP1R2
PPP1R9B
PPP2R5A
PPP3R1
PPT1
PPY
PQBP1
PRAME
PRAP1
PRC1
PRCP
PRDX1
PRDX2
PRDX3
PRDX5
PRDX6
PREB
PRELP
PRG2
PRG3
PRKAB1
PRKAG3
PRKAR1A
PRKAR2A
PRKCQ
PRKD2
PRKG1
PRKRA
PRL
PRND
PROC
PROCR
PROK1
PROS1
PRR4
PRR5
PRRT3
PRSS2
PRSS22
PRSS27
PRSS53
PRSS8
PRTFDC1
PRTG
PRTN3
PRUNE2
PSAP
PSAPL1
PSCA
PSG1
PSIP1
PSMA1
PSMC3
PSMD1
PSMD5
PSMD9
PSME1
PSME2
PSMG3
PSMG4
PSPN
PSRC1
PSTPIP2
PTEN
PTGDS
PTGES2
PTGR1
PTH
PTH1R
PTK7
PTN
PTP4A3
PTPN1
PTPN6
PTPN9
PTPRB
PTPRC
PTPRF
PTPRH
PTPRK
count

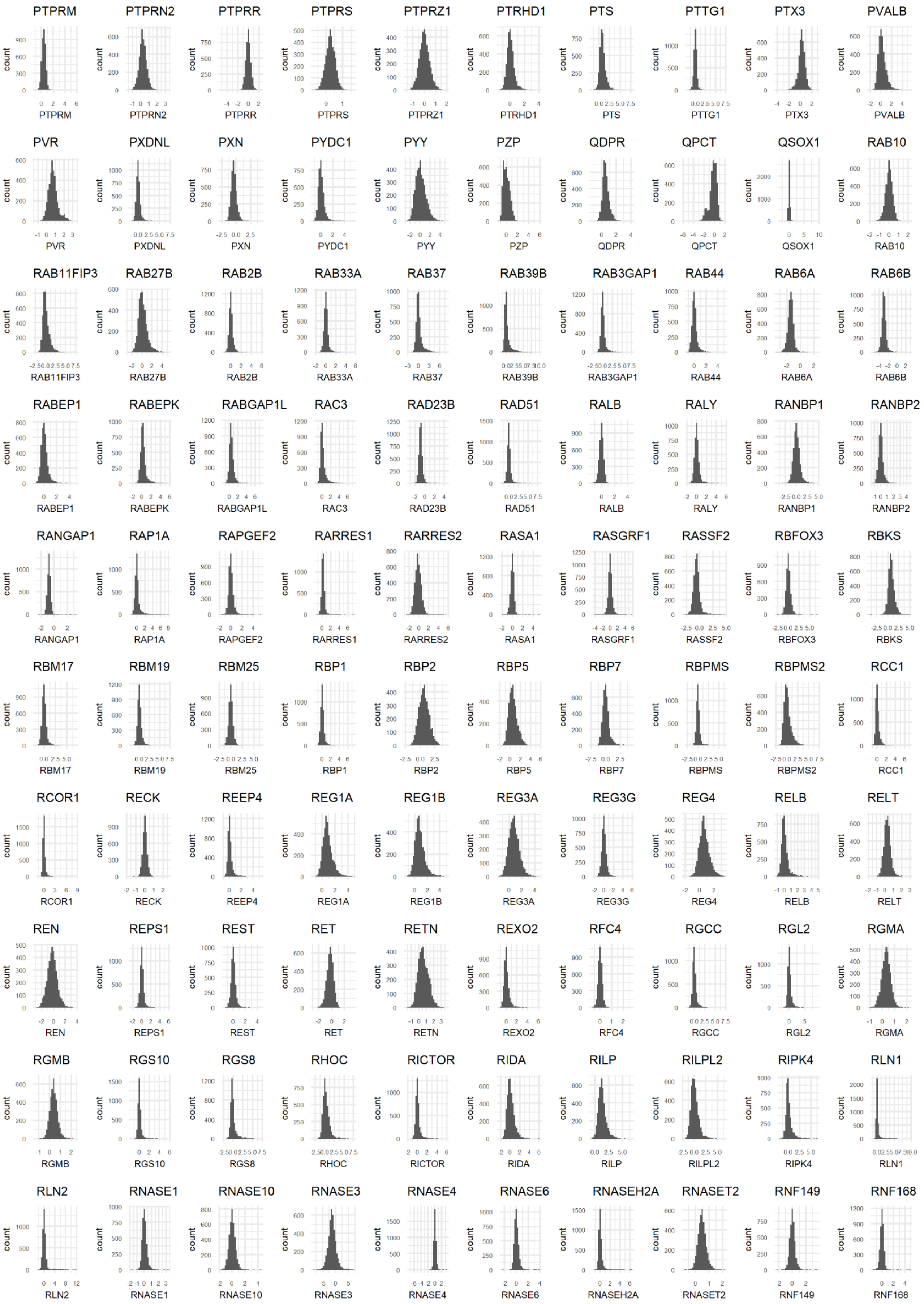
PTPRM PTPRN2 PTPRR PTPRS PTPRZ1 PTRHD1 PTS PTTG1 PTX3 PVALB
PVR PXDNL PXN PYDC1 PYY PZP QDPR QPCT QSOX1 RAB10
RAB11FIP3 RAB27B RAB2B RAB33A RAB37 RAB39B RAB3GAP1 RAB44 RAB6A RAB6B
RABEP1 RABEPK RABGAP1L RAC3 RAD23B RAD51 RALB RALY RANBP1 RANBP2
RANGAP1 RAP1A RAPGEF2 RARRES1 RARRES2 RASA1 RASGRF1 RASSF2 RBFOX3 RBKS
RBM17 RBM19 RBM25 RBP1 RBP2 RBP5 RBP7 RBPMS RBPMS2 RCC1
RCOR1 RECK REEP4 REG1A REG1B REG3A REG3G REG4 RELB RELT
REN REPS1 REST RET RETN REXO2 RFC4 RGCC RGL2 RGMA
RGMB RGS10 RGS8 RHOC RICTOR RIDA RILP RILPL2 RIPK4 RLN1
RLN2 RNASE1 RNASE10 RNASE3 RNASE4 RNASE6 RNASEH2A RNASET2 RNF149 RNF168

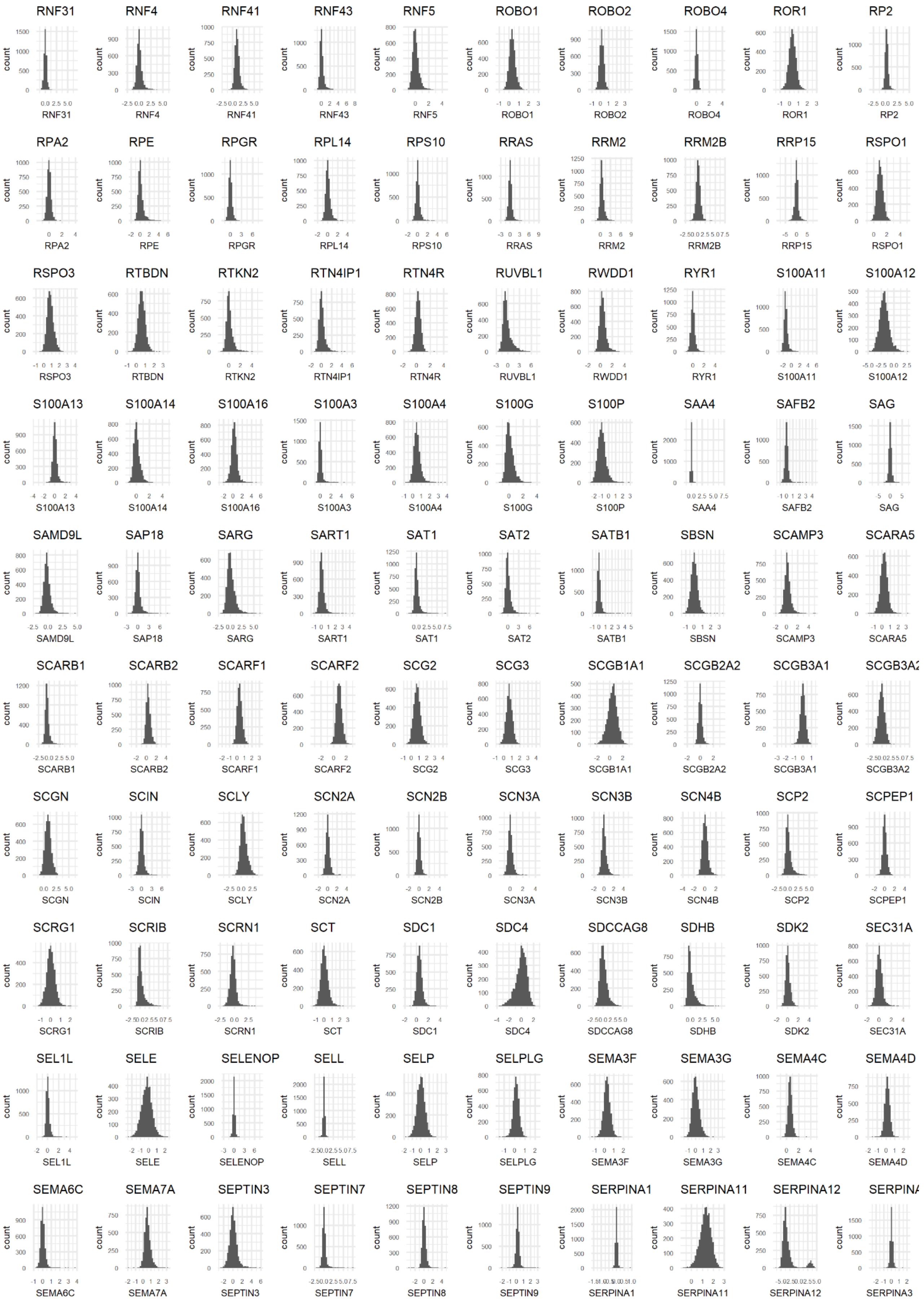
RNF31 RNF4 RNF41 RNF43 RNF5 ROBO1 ROBO2 ROBO4 ROR1 RP2
RPA2 RPE RPGR RPL14 RPS10 RRAS RRM2 RRM2B RRP15 RSPO1
RSPO3 RTBDN RTKN2 RTN4IP1 RTN4R RUVBL1 RWDD1 RYR1 S100A11 S100A12
S100A13 S100A14 S100A16 S100A3 S100A4 S100G S100P SAA4 SAFB2 SAG
SAMD9L SAP18 SARG SART1 SAT1 SAT2 SATB1 SBSN SCAMP3 SCARA5
SCARB1 SCARB2 SCARF1 SCARF2 SCG2 SCG3 SCGB1A1 SCGB2A2 SCGB3A1 SCGB3A2
SCGN SCIN SCLY SCN2A SCN2B SCN3A SCN3B SCN4B SCP2 SCPEP1
SCRG1 SCRIB SCRN1 SCT SDC1 SDC4 SDCCAG8 SDHB SDK2 SEC31A
SEL1L SELE SELENOP SELL SELP SELPLG SEMA3F SEMA3G SEMA4C SEMA4D
SEMA6C SEMA7A SEPTIN3 SEPTIN7 SEPTIN8 SEPTIN9 SERPINA1 SERPINA11 SERPINA12 SERPINA3
count

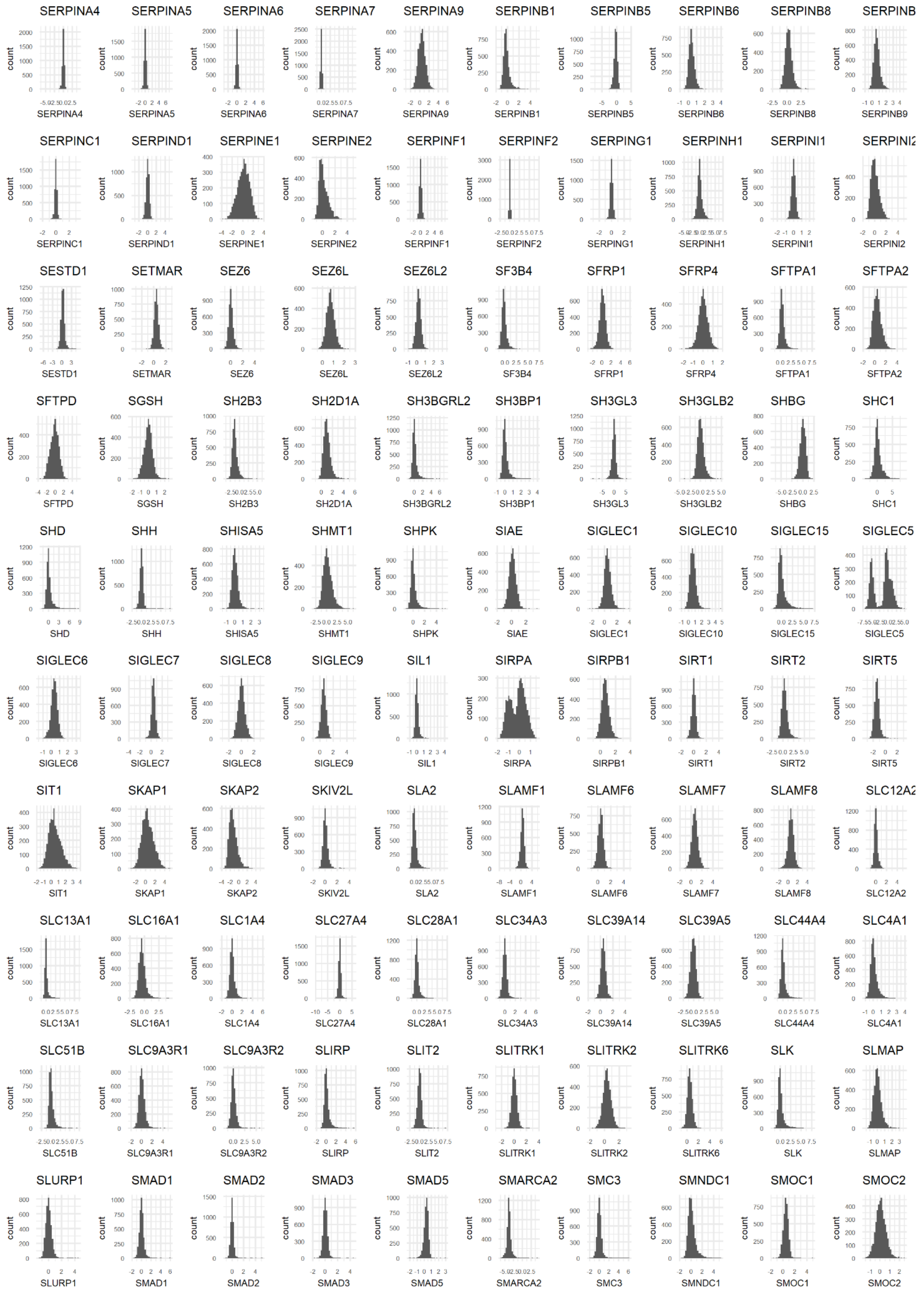
SERPINA4 SERPINA5 SERPINA6 SERPINA7 SERPINA9 SERPINB1 SERPINB5 SERPINB6 SERPINB8 SERPINB9
SERPINC1 SERPIND1 SERPINE1 SERPINE2 SERPINF1 SERPINF2 SERPING1 SERPINH1 SERPINI1 SERPINI2
SESTD1 SETMAR SEZ6 SEZ6L SEZ6L2 SF3B4 SFRP1 SFRP4 SFTPA1 SFTPA2
SFTPD SGSH SH2B3 SH2D1A SH3BGRL2 SH3BP1 SH3GL3 SH3GLB2 SHBG SHC1
SHD SHH SHISA5 SHMT1 SHPK SIAE SIGLEC1 SIGLEC10 SIGLEC15 SIGLEC5
SIGLEC6 SIGLEC7 SIGLEC8 SIGLEC9 SIL1 SIRPA SIRPB1 SIRT1 SIRT2 SIRT5
SIT1 SKAP1 SKAP2 SKIV2L SLA2 SLAMF1 SLAMF6 SLAMF7 SLAMF8 SLC12A2
SLC13A1 SLC16A1 SLC1A4 SLC27A4 SLC28A1 SLC34A3 SLC39A14 SLC39A5 SLC44A4 SLC4A1
SLC51B SLC9A3R1 SLC9A3R2 SLIRP SLIT2 SLITRK1 SLITRK2 SLITRK6 SLK SLMAP
SLURP1 SMAD1 SMAD2 SMAD3 SMAD5 SMARCA2 SMC3 SMNDC1 SMOC1 SMOC2
count

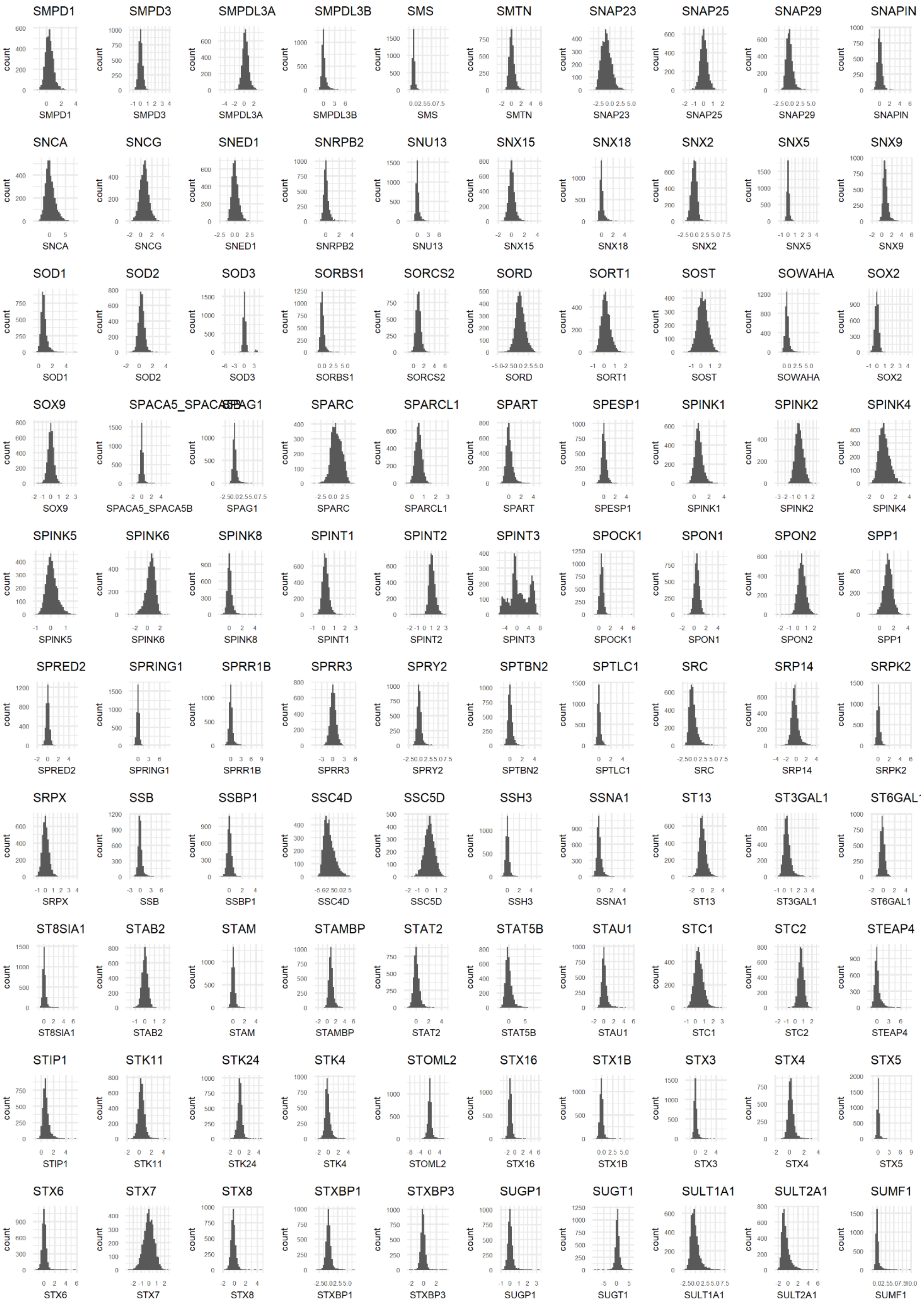
SMPD1
SMPD3
SMPDL3A
SMPDL3B
SMS
SMTN
SNAP23
SNAP25
SNAP29
SNAPIN
SNCA
SNCG
SNED1
SNRPB2
SNU13
SNX15
SNX18
SNX2
SNX5
SNX9
SOD1
SOD2
SOD3
SORBS1
SORCS2
SORD
SORT1
SOST
SOWAHA
SOX2
SOX9
SPACA5_SPACA5B
SPAG1
SPARC
SPARCL1
SPART
SPESP1
SPINK1
SPINK2
SPINK4
SPINK5
SPINK6
SPINK8
SPINT1
SPINT2
SPINT3
SPOCK1
SPON1
SPON2
SPP1
SPRED2
SPRING1
SPRR1B
SPRR3
SPRY2
SPTBN2
SPTLC1
SRC
SRP14
SRPK2
SRPX
SSB
SSBP1
SSC4D
SSC5D
SSH3
SSNA1
ST13
ST3GAL1
ST6GAL1
ST8SIA1
STAB2
STAM
STAMBP
STAT2
STAT5B
STAU1
STC1
STC2
STEAP4
STIP1
STK11
STK24
STK4
STOML2
STX16
STX1B
STX3
STX4
STX5
STX6
STX7
STX8
STXBP1
STXBP3
SUGP1
SUGT1
SULT1A1
SULT2A1
SUMF1
count

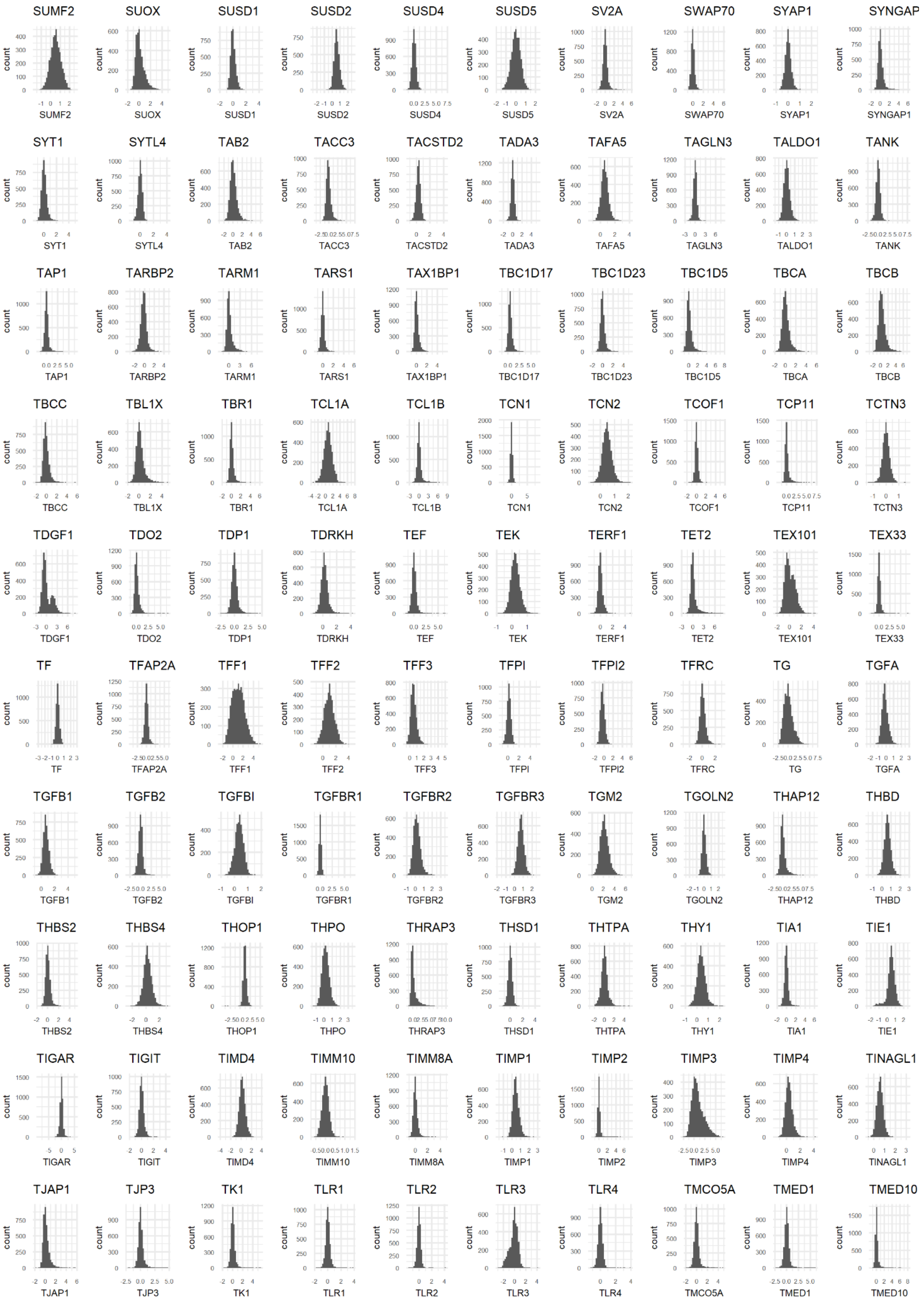
SUMF2
SUOX
SUSD1
SUSD2
SUSD4
SUSD5
SV2A
SWAP70
SYAP1
SYNGAP
SYT1
SYTL4
TAB2
TACC3
TACSTD2
TADA3
TAFA5
TAGLN3
TALDO1
TANK
TAP1
TARBP2
TARM1
TARS1
TAX1BP1
TBC1D17
TBC1D23
TBC1D5
TBCA
TBCB
TBCC
TBL1X
TBR1
TCL1A
TCL1B
TCN1
TCN2
TCOF1
TCP11
TCTN3
TDGF1
TDO2
TDP1
TDRKH
TEF
TEK
TERF1
TET2
TEX101
TEX33
TF
TFAP2A
TFF1
TFF2
TFF3
TFPI
TFPI2
TFRC
TG
TGFA
TGFB1
TGFB2
TGFBI
TGFBR1
TGFBR2
TGFBR3
TGM2
TGOLN2
THAP12
THBD
THBS2
THBS4
THOP1
THPO
THRAP3
THSD1
THTPA
THY1
TIA1
TIE1
TIGAR
TIGIT
TIMD4
TIMM10
TIMM8A
TIMP1
TIMP2
TIMP3
TIMP4
TINAGL1
TJAP1
TJP3
TK1
TLR1
TLR2
TLR3
TLR4
TMCO5A
TMED1
TMED10

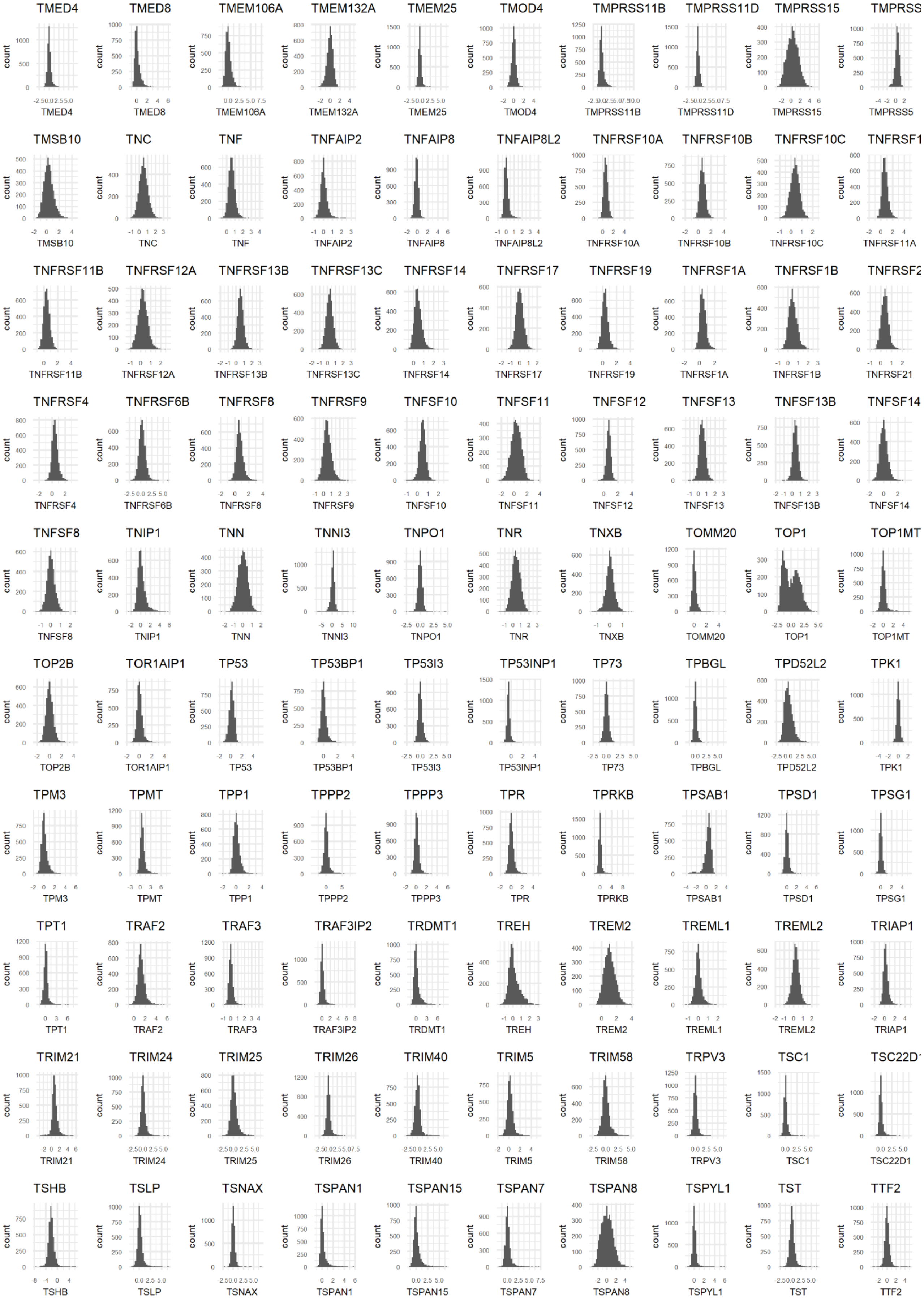
TMED4
TMED8
TMEM106A
TMEM132A
TMEM25
TMOD4
TMPRSS11B
TMPRSS11D
TMPRSS15
TMPRSS5
TMSB10
TNC
TNF
TNFAIP2
TNFAIP8
TNFAIP8L2
TNFRSF10A
TNFRSF10B
TNFRSF10C
TNFRSF11A
TNFRSF11B
TNFRSF12A
TNFRSF13B
TNFRSF13C
TNFRSF14
TNFRSF17
TNFRSF19
TNFRSF1A
TNFRSF1B
TNFRSF21
TNFRSF4
TNFRSF6B
TNFRSF8
TNFRSF9
TNFSF10
TNFSF11
TNFSF12
TNFSF13
TNFSF13B
TNFSF14
TNFSF8
TNIP1
TNN
TNNI3
TNPO1
TNR
TNXB
TOMM20
TOP1
TOP1MT
TOP2B
TOR1AIP1
TP53
TP53BP1
TP53I3
TP53INP1
TP73
TPBGL
TPD52L2
TPK1
TPM3
TPMT
TPP1
TPPP2
TPPP3
TPR
TPRKB
TPSAB1
TPSD1
TPSG1
TPT1
TRAF2
TRAF3
TRAF3IP2
TRDMT1
TREH
TREM2
TREML1
TREML2
TRIAP1
TRIM21
TRIM24
TRIM25
TRIM26
TRIM40
TRIM5
TRIM58
TRPV3
TSC1
TSC22D1
TSHB
TSLP
TSNAX
TSPAN1
TSPAN15
TSPAN7
TSPAN8
TSPYL1
TST
TTF2
count

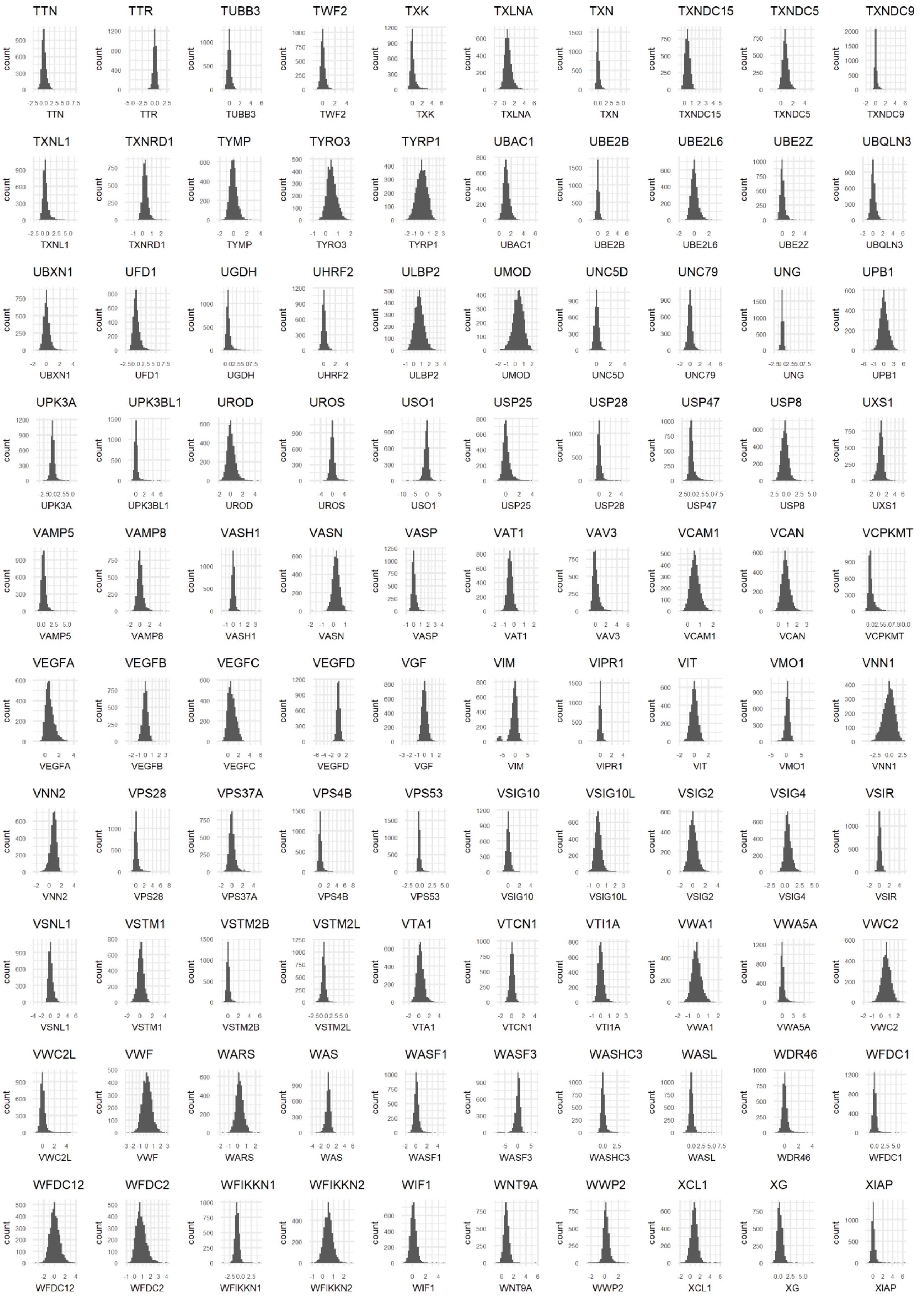
TTN TTR TUBB3 TWF2 TXK TXLNA TXN TXNDC15 TXNDC5 TXNDC9
TXNL1 TXNRD1 TYMP TYRO3 TYRP1 UBAC1 UBE2B UBE2L6 UBE2Z UBQLN3
UBXN1 UFD1 UGDH UHRF2 ULBP2 UMOD UNC5D UNC79 UNG UPB1
UPK3A UPK3BL1 UROD UROS USO1 USP25 USP28 USP47 USP8 UXS1
VAMP5 VAMP8 VASH1 VASN VASP VAT1 VAV3 VCAM1 VCAN VCPKMT
VEGFA VEGFB VEGFC VEGFD VGF VIM VIPR1 VIT VMO1 VNN1
VNN2 VPS28 VPS37A VPS4B VPS53 VSIG10 VSIG10L VSIG2 VSIG4 VSIR
VSNL1 VSTM1 VSTM2B VSTM2L VTA1 VTCN1 VTI1A VWA1 VWA5A VWC2
VWC2L VWF WARS WAS WASF1 WASF3 WASHC3 WASL WDR46 WFDC1
WFDC12 WFDC2 WFIKKN1 WFIKKN2 WIF1 WNT9A WWP2 XCL1 XG XIAP
count

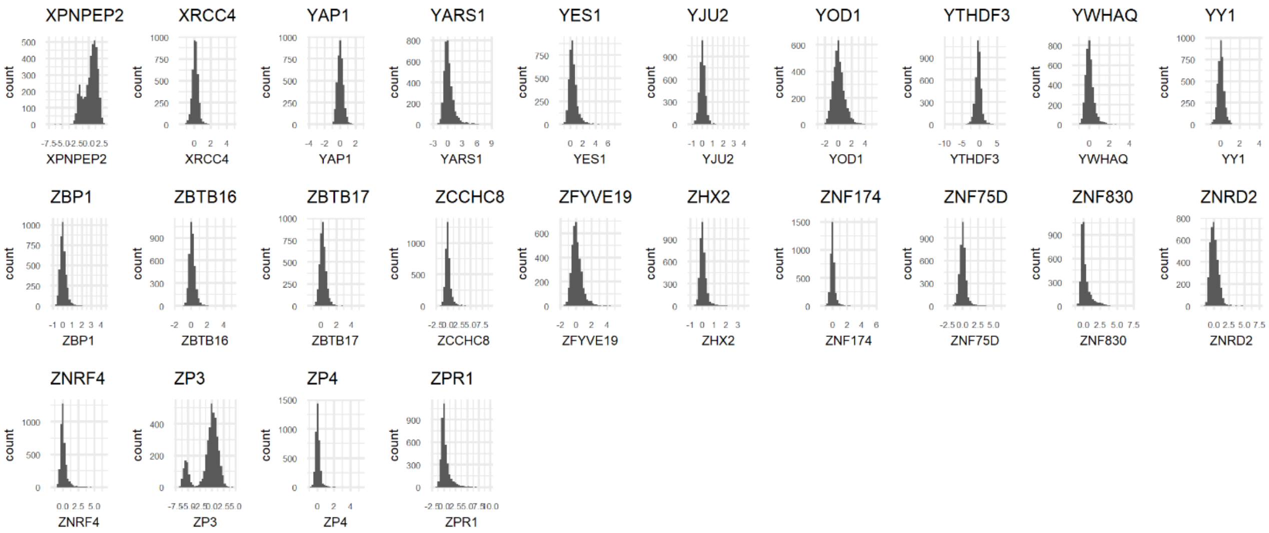
XPNPEP2
XRCC4
YAP1
YARS1
YES1
YJU2
YOD1
YTHDF3
YWHAQ
YY1
ZBP1
ZBTB16
ZBTB17
ZCCHC8
ZFYVE19
ZHX2
ZNF174
ZNF75D
ZNF830
ZNRD2
ZNRF4
ZP3
ZP4
ZPR1
count
