# Supplementary material for: Proteo-genomic analyses in relatively lean Chinese adults identify proteins and pathways that affect general and central adiposity levels
Source: Commun Biol. 2024 Oct 15;7:1327. doi: 10.1038/s42003-024-06984-y (PMC11480319; doi:10.1038/s42003-024-06984-y)
Supplement: Supplementary file 3 — Description of Additional Supplementary File [file 42003_2024_6984_MOESM3_ESM.pdf]

**Description Of Additional Supplementary Files**

**File name:** Supplementary Data 1
**Description:** Difference in concentrations of 2944 proteins associated with 1-SD higher BMI, BF%, WC and WHR in conventional and genetic analyses

**File name**: Supplementary Data 2
**Description:** MR sensitivity analyses

**File name:** Supplementary Data 3
**Description:** List of significantly enriched biological process terms of adiposity-related proteins

**File name:** Supplementary Data 4
**Description:** PheWAS associations of cis-pQTL for proteins showing causal relationship with general and central adiposity in adiposity-to-protein direction after Bonferroni correction

**File name:** Supplementary Data 5
**Description**: List of 2944 proteins quantified by the Olink Explore I and Olink Explore II assay
