# Supplementary material for: Proteo-genomic analyses in relatively lean Chinese adults identify proteins and pathways that affect general and central adiposity levels
Source: Commun Biol. 2024 Oct 15;7:1327. doi: 10.1038/s42003-024-06984-y (PMC11480319; doi:10.1038/s42003-024-06984-y)
Supplement: Supplementary file 9 — Reporting summary [file 42003_2024_6984_MOESM9_ESM.pdf]

nature portfolio

Corresponding author(s): Zhengming Chen

Last updated by author(s): Jul 10, 2024

Reporting Summary

Nature Portfolio wishes to improve the reproducibility of the work that we publish. This form provides structure for consistency and transparency in reporting. For further information on Nature Portfolio policies, see our Editorial Policies and the Editorial Policy Checklist.

Statistics

For all statistical analyses, confirm that the following items are present in the figure legend, table legend, main text, or Methods section.

n/a	Confirmed	
☐	☒	The exact sample size (*n*) for each experimental group/condition, given as a discrete number and unit of measurement
☐	☒	A statement on whether measurements were taken from distinct samples or whether the same sample was measured repeatedly
☐	☒	The statistical test(s) used AND whether they are one- or two-sided<br>*Only common tests should be described solely by name; describe more complex techniques in the Methods section.*
☐	☒	A description of all covariates tested
☐	☒	A description of any assumptions or corrections, such as tests of normality and adjustment for multiple comparisons
☐	☒	A full description of the statistical parameters including central tendency (e.g. means) or other basic estimates (e.g. regression coefficient) AND variation (e.g. standard deviation) or associated estimates of uncertainty (e.g. confidence intervals)
☐	☒	For null hypothesis testing, the test statistic (e.g. *F*, *t*, *r*) with confidence intervals, effect sizes, degrees of freedom and *P* value noted<br>*Give P values as exact values whenever suitable.*
☒	☐	For Bayesian analysis, information on the choice of priors and Markov chain Monte Carlo settings
☒	☐	For hierarchical and complex designs, identification of the appropriate level for tests and full reporting of outcomes
☐	☒	Estimates of effect sizes (e.g. Cohen's *d*, Pearson's *r*), indicating how they were calculated

Our web collection on statistics for biologists contains articles on many of the points above.

Software and code

Policy information about availability of computer code

Data collection	A range of bespoke CKB IT systems was used for data collection (see https://www.ckbiobank.org/study-resources/management-systems)
Data analysis	Observational and genetic analyses were conducted using R (version 4.1.2; https://www.r-project.org/), employing the 'Epi', 'tidyverse', 'ggplot2', and 'ckbplotr' packages, as well as custom code. For MR sensitivity analyses, we used the publicly available MR and MR-PRESSO R packages. For GO and KEGG enrichment analyses, we utilized clusterProfiler (v4.2.2). Fine-mapping was performed using the Sum of Single Effects model (SuSiE; susieR v0.12.16 R package). PhenoScanner (v2) was employed to investigate associations of cis-pQTLs with a range of phenotypes.

For manuscripts utilizing custom algorithms or software that are central to the research but not yet described in published literature, software must be made available to editors and reviewers. We strongly encourage code deposition in a community repository (e.g. GitHub). See the Nature Portfolio guidelines for submitting code & software for further information.

Data

Policy information about availability of data

All manuscripts must include a data availability statement. This statement should provide the following information, where applicable:

- Accession codes, unique identifiers, or web links for publicly available datasets
- A description of any restrictions on data availability
- For clinical datasets or third party data, please ensure that the statement adheres to our policy

The China Kadoorie Biobank (CKB) is a global resource for the investigation of lifestyle, environmental, blood biochemical and genetic factors as determinants of common diseases. CKB is committed to making the data available to the wider scientific community after a period of exclusive use by study investigators (see CKB data sharing policy at http://www.ckbiobank.org/site/Data+Access). The proteomics summary data (e.g. protein pQTLs) will be made available through a newly developed CKB PheWeb in due course .

Research involving human participants, their data, or biological material

Policy information about studies with human participants or human data. See also policy information about sex, gender (identity/presentation), and sexual orientation and race, ethnicity and racism.

Reporting on sex and gender	The CKB collects information on the biological sex of participants based on self-reports. Main analyses were conducted on both female and male participants combined. Sex-specific analyses are reported in the supplementary material; however, due to the small sample size, our ability to conduct further detailed analyses, particularly MR analyses, is limited.
Reporting on race, ethnicity, or other socially relevant groupings	All participants of CKB were of Chinese ethnicity based on self report.
Population characteristics	CKB recruitment is described in Chen et al., PMID: 22158673.
Recruitment	CKB recruitment is described in Chen et al., PMID: 22158673.
Ethics oversight	The China Kadoorie Biobank (CKB) complies with all required ethical standards for medical research on human subjects. Ethical approval was obtained from the Ethical Review Committee of the Chinese Centre for Disease Control and Prevention (Beijing, China, 005/2004) and the Oxford Tropical Research Ethics Committee, University of Oxford (UK, 025-04). All participants provided written informed consent.

Note that full information on the approval of the study protocol must also be provided in the manuscript.

Field-specific reporting

Please select the one below that is the best fit for your research. If you are not sure, read the appropriate sections before making your selection.

☒ Life sciences ☐ Behavioural & social sciences ☐ Ecological, evolutionary & environmental sciences

For a reference copy of the document with all sections, see nature.com/documents/nr-reporting-summary-flat.pdf

Life sciences study design

All studies must disclose on these points even when the disclosure is negative.

Sample size	The present analysis involved a case-subcohort study of 3,977 unrelated participants (1,951 incident IHD cases and 2,026 subcohort participants) from China Kadoorie Biobank.
Data exclusions	For the main analyses we did not exclude any participants. In a sensitivity analyses we excluded 1,951 IHD cases.
Replication	Over 90% of our findings were successfully replicated using data from the UK Biobank, which also includes protein data on the same Olink platform.
Randomization	Randomization was not directly relevant to this study design.
Blinding	The data provided to researchers did not contain any personally identifiable variables i.e. datasets were anonymized with uniquely encrypted participant identifiers.

Reporting for specific materials, systems and methods

We require information from authors about some types of materials, experimental systems and methods used in many studies. Here, indicate whether each material, system or method listed is relevant to your study. If you are not sure if a list item applies to your research, read the appropriate section before selecting a response.

Materials & experimental systems

n/a	Involved in the study
☒	☐ Antibodies
☒	☐ Eukaryotic cell lines
☒	☐ Palaeontology and archaeology
☒	☐ Animals and other organisms
☒	☐ Clinical data
☒	☐ Dual use research of concern
☒	☐ Plants

Methods

n/a	Involved in the study
☒	☐ ChIP-seq
☒	☐ Flow cytometry
☒	☐ MRI-based neuroimaging

Plants

Seed stocks	N/A
Novel plant genotypes	N/A
Authentication	N/A
